# The equity implications of exposure to industrial livestock operations

**DOI:** 10.1371/journal.pone.0342552

**Published:** 2026-02-19

**Authors:** Kay Jowers, Yu Ma, Christopher Timmins

**Affiliations:** 1 Kenan Institute for Ethics, Duke University, Durham, North Carolina, United States of America; 2 Environmental Sciences Division, Oak Ridge National Laboratory, Oak Ridge, Tennessee, United States of America; 3 Wisconsin School of Business, University of Wisconsin–Madison, Madison, Wisconsin, United States of America; University of South Carolina, UNITED STATES OF AMERICA

## Abstract

Concentrated animal feeding operations, particularly hog and poultry farms, have expanded rapidly in North Carolina in recent decades. The air and water pollution they generate cause many environmental and health problems for local communities [40 CFR sec. 122.23(b); US Government Accountability Office (2008)]. Combining farm-level data with address- and household-level data on race and income in North Carolina, we non-parametrically describe exposure to hog and poultry CAFOs by race-income characteristics. We further examine the differences in exposure by household water sources – private wells versus community water systems. Results show strong evidence of disproportionate exposure for low-income Hispanic relative to white households. This exposure gap falls with income, but the gap between African American and white households rises with income, particularly in the case of hogs. These gaps in exposure are larger for those households dependent upon private wells.

## Introduction

Concentrated animal feeding operations (CAFOs) are livestock production facilities where large numbers of animals are raised in confined spaces. Unlike traditional farming, where animals are typically grazed in an open pasture or other appropriate space, CAFOs keep animals in densely-packed indoor spaces where their movement is restricted. Systems are designed to collect and dispose of large quantities of waste. Prompted not only by industry changes in the 1980s and 1990s aimed at increasing the efficiency of livestock farming but also significantly influenced by shifts in the tobacco industry, the swine industry in North Carolina (NC) has undergone rapid growth as farmers sought to diversify into livestock, notably swine production [1]. This change led NC to become the third-largest hog-producing state in the nation, trailing only behind Iowa and Minnesota [2–4]. Notably, NC imposed a moratorium on new hog CAFOs in 1997 and made it permanent in 2007 [5], reflecting growing recognition of their environmental and community impacts.

The state is also a top poultry producer in the United States (US), with no similar moratorium on new poultry operations [6]. According to the NC Department of Environmental Quality (DEQ), poultry production increased significantly in eastern NC between 1992 and 2014, with some river basins experiencing nearly 400 percent growth in the number of birds [7]. Consequently, Eastern NC now hosts a high concentration of both swine and poultry CAFOs. These operations are often situated closer to residential communities than in other states with comparable CAFO densities, raising concerns about their proximity and potential environmental and health impacts on these communities [8–10] (see SI.File on “Environmental and health impacts of CAFOs.” for a detailed discussion on the impacts of CAFOs).

Historically, the issue of CAFOs in North Carolina has been a subject of extensive litigation, reflecting ongoing conflicts between environmental concerns and agricultural practices. Notably, high-profile lawsuits against major operators like Smithfield even resulted in a multi-year effort to identify technologies that would make significant upgrades to waste management systems [11]. In recent years, North Carolina has witnessed significant legal and policy developments concerning CAFOs. Notably, a series of lawsuits culminated in substantial damages being awarded to neighbors of CAFOs, citing the adverse impacts of these operations on local communities [12]. These legal actions underscored the growing concerns about the environmental and health effects of CAFOs on nearby residents [13]. In response to these developments, the North Carolina General Assembly amended the state’s Right to Farm Act to expand protections for CAFOs against such lawsuits. This legislative change, detailed in [14], reflects the ongoing tension between agricultural interests and community welfare in the state. The evolving legal landscape highlights the complex challenges in balancing the economic benefits of CAFOs with their environmental and social impacts on nearby residents.

We examine the relationship between race and income and exposure to CAFOs. Our study targets 43 counties in Eastern North Carolina, home to about 96% of the state’s hog farms and 38.2% of poultry farms, to analyze the co-occurrence of hogs and poultry. This selection provides an optimal region for observing the co-existence of both types of CAFOs, despite poultry sites being more evenly dispersed statewide. In studies of the siting of hog farms, researchers looking at the relationship between the presence of a hog CAFO and the race-based and class-based characteristics of nearby residents have found the operations are disproportionately located in areas with higher levels of poverty [15,16] and higher proportions of people of color (e.g., Black, Hispanic, and Native American populations) [4,15–17]. However, that literature has faced some limitations. First, previous studies have had to rely on Census demographic data to measure the relationship between hog exposure and race [4,16,18] *or* income [16,18]. Importantly, data limitations prevented analysis of how disproportionate exposure varies by race ***and*** income at a very local level. Using a novel micro-data source, we add this dimension to our analysis.

Second, the relationship between race and exposure to poultry CAFOs has largely been missing in previous studies. We collect information on hog and poultry farms and separately describe exposure to both. We also run a series of bivariate probit models to examine the likelihood of multiple overlapping exposures — i.e., the joint probability of being exposed to neither, either, or both types of CAFOs.

Finally, because water contamination is considered as an important exposure route (see SI.File on “Environmental and health impacts of CAFOs.” for a detailed discussion on the different waste management systems of hogs and poultry and their associated environmental and health impacts), we investigate how exposure patterns vary by water source — i.e., community water systems versus private wells (see Materials and methods). Public water systems typically have treatment processes to mitigate contamination, while private wells do not benefit from such protections (EPA). Our motivation is to not only to account for the varying risks of contamination but also to provide a more nuanced understanding of how these risks intersect with socioeconomic factors. By analyzing households with private wells and those with community water systems separately, we aim to uncover any disparities in exposure and potential health risks that might be obscured in a more aggregated analysis.

## Results

Previous research has documented inequity in exposure to CAFO’s along the lines of race and poverty [4,16]. We begin by confirming these inequities using our data pertaining to 2014 (see Materials and methods), considering exposure defined simply as the number of animals — either hog steady state live weight (SSLW) or poultry bird count — within a buffer of 3, 4 or 5 km. In particular, we are interested in how exposure varies for race, income, and water sources. Household race is defined as white, Black, or Hispanic based on the name and geographic location of the household. We separately demonstrate the inequities in exposure for hog and poultry farms in our analyses.

**Fig 1:**
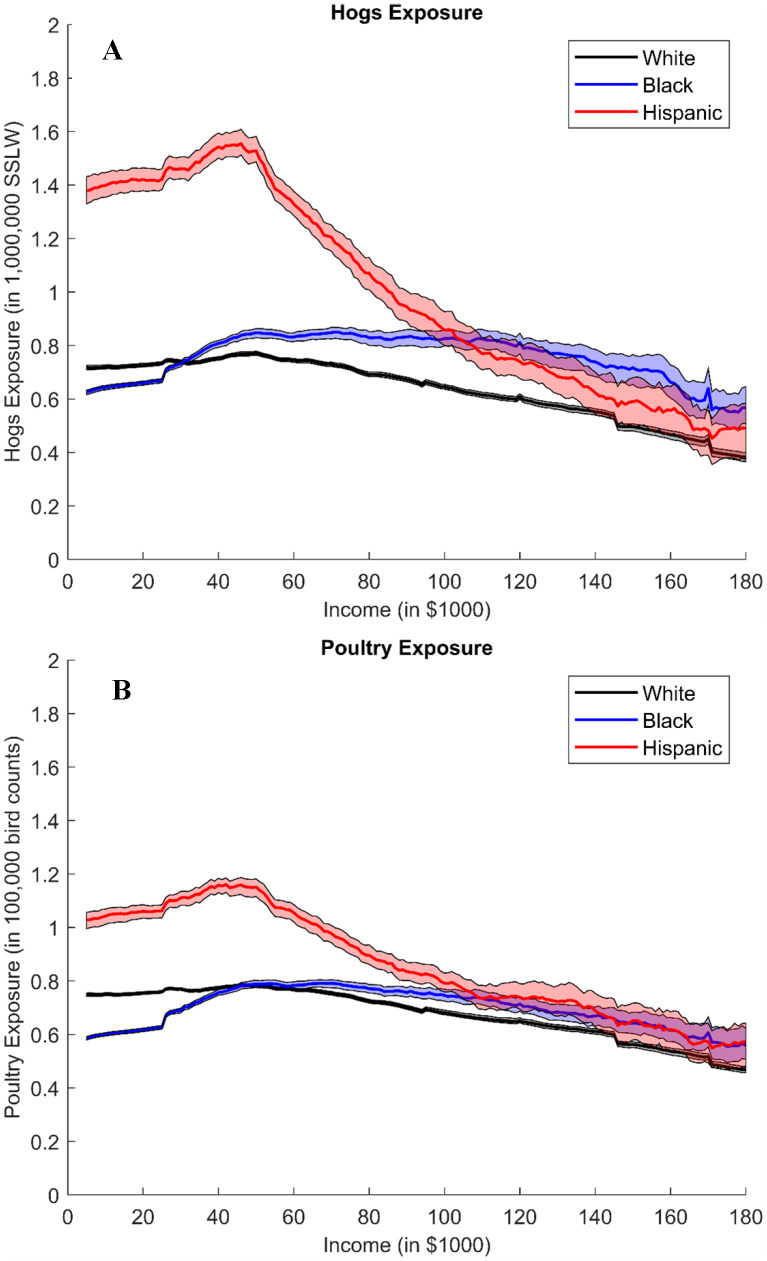
5km Hogs and Poultry Exposure, by Race and Income. The two panels show the 5 km hog/poultry exposure distribution for all households. The solid line shows the exposure, and shaded area shows the confidence interval. Exposure plots for owners/renters (see Figs S7-S9 in S1 File) and 3 km and 4 km buffers (see Figs S5 and S6 in S1 File) are in supplementary information. **(A): Hog Exposure, by Race and Income**. We use animal farm data (provided by NC Department of Environmental Quality) and household demographic data (InfoUSA) to calculate the aggregated SSLW (in 1,000,000) within household’s 5 km buffer and corresponding 95% confidence interval at each income level for each race (white, Black, and Hispanic). **(B): Poultry Exposure, by Race and Income**. We use animal farm data (provided by Environmental Working Group) and household demographic data (InfoUSA) to calculate the aggregated bird counts (in 100,000) within household’s 5 km buffer and corresponding 95% confidence interval at each income level for each race (white, Black, and Hispanic).

We plot the actual CAFO exposure at each income level for each race and bootstrap the corresponding 95% confidence intervals. To smooth the plots, we use a moving average calculation to average the points at nearby income levels for each income. The graphs in Fig 1 describe 5 km exposures; plots for 3 km and 4 km are in the supplementary information (see Figs S5 and S6 in S1 File). The two panels compare CAFO exposures across race groups plotted separately by hogs and poultry for all households. The graphs suggest minorities, especially Hispanic households, face higher hog and poultry exposure than whites. For example, the exposures could go up to 1,400,000 SSLW and 110,000 bird counts for Hispanic households with income below $40,000, while only 800,000 SSLW and 80,000 bird counts for low-income whites. For both Hispanic and white households, the exposures decrease linearly and uniformly with income. However, for Black residents, the relationship between income and exposure does not follow a linear or uniformly decreasing trend. We observe that the disparity initially increases until an income level of about $60,000, then appears to level off between $60,000 and $130,000. Interestingly, then it escalates to its highest level at around $160,000, before eventually declining.

Because of the possible difference between owners and renters, we also plot separately for owners and renters. The exposure patterns are similar when we compare owners and renters, and the graphs are in the supplementary information (see Figs S7-S9 in S1 File). We conduct additional analysis (see Fig S25 in S1 File) by focusing on the top agricultural-producing counties in livestock farm cash receipts [19]. The top ten counties are Duplin, Sampson, Bladen, Union, Robeson, Wilkes, Wayne, Anson, Randolph, and Bertie. Results suggest the exposure is higher for those living in major agricultural-producing counties.

**Fig 2:**
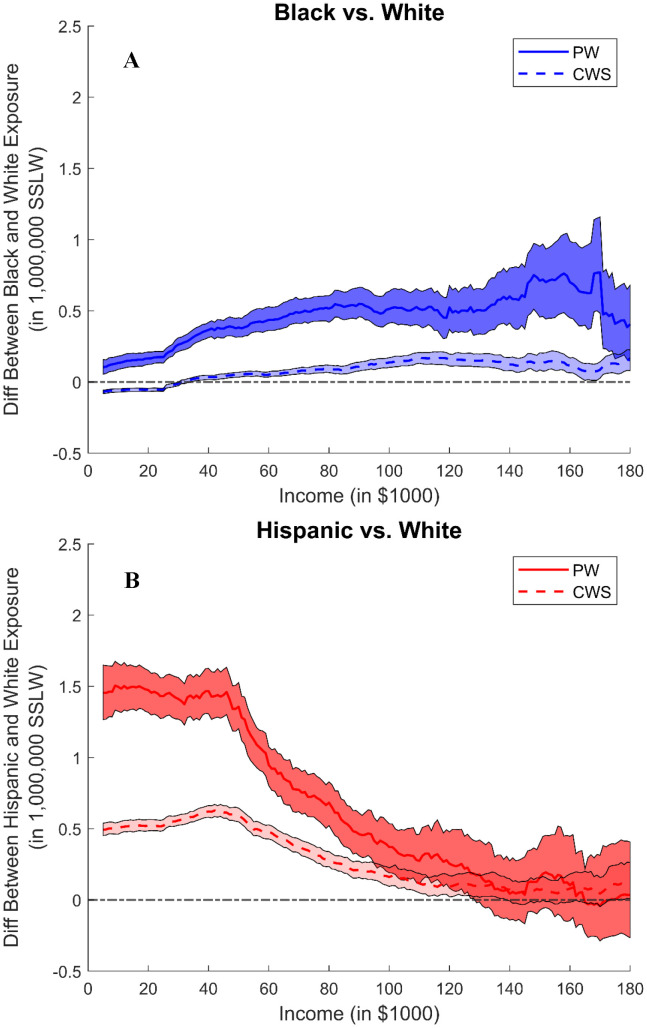
5km Hogs Exposure Race Difference, Private Well vs Community Water System. The two panels show the 5 km hog exposure difference (in 1,000,000 SSLW) between minority group and white for all households. Exposure difference plots for owners/renters (see Figs S19-S21 in S1 File) and 3 km and 4 km buffers (see Figs S16 and S17 in S1 File) are in supplementary information. **(A): 5 km Hog Exposure Race Difference between Black and White Households, Private Well vs Community Water System**. The line shows the hog exposure differences between Black and white residents, where the solid and dashed lines show the exposure differences for households dependent upon private wells versus community water systems. The shaded area shows the corresponding confidence interval. A positive value indicates the exposure is higher for Black residents, compared to white residents. **(B): 5 km Hog Exposure Race Difference between Hispanic and White Households, Private Well vs Community Water System**. The line shows the hog exposure differences between Hispanic and white residents, where the solid and dashed lines show the exposure differences for households dependent upon private wells versus community water systems. The shaded area shows the corresponding confidence interval. A positive value indicates the exposure is higher for Hispanic residents, compared to white residents.

**Fig 3:**
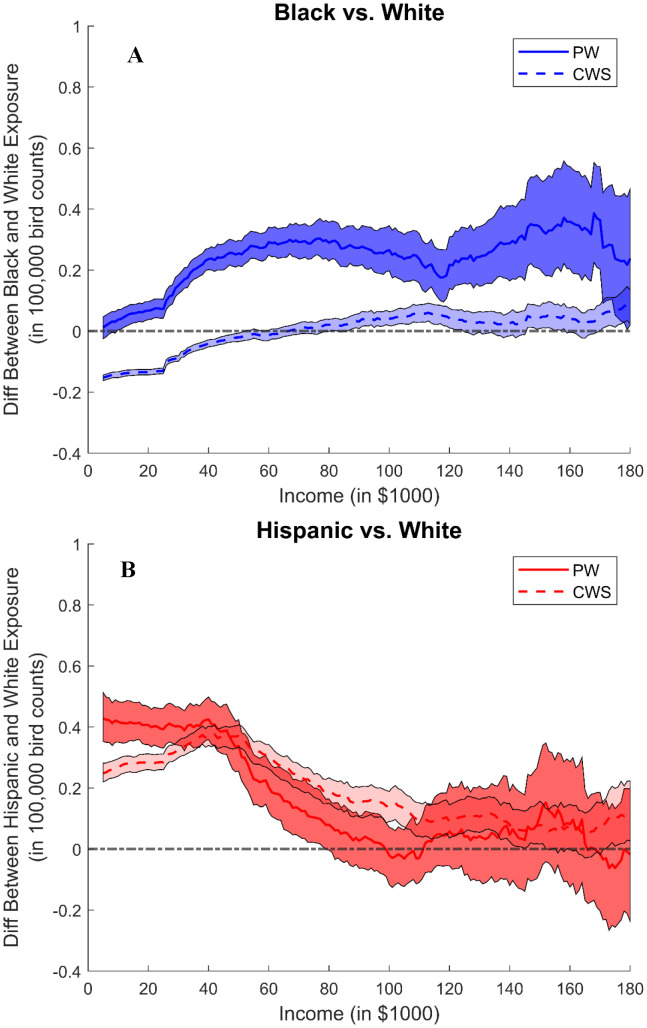
5km Poultry Exposure Race Difference, Private Well vs Community Water System. The two panels show the 5 km poultry exposure difference (in 100,000 bird counts) between minority group and white for all households. Exposure difference plots for owners/renters (see Figs S22-S24 in S1 File) and 3 km and 4 km buffers (see Figs S16 and S17 in S1 File) are in supplementary information. **(A): 5 km Poultry Exposure Race Difference between Black and White Households, Private Well vs Community Water System**. The line shows the poultry exposure differences between Black and white residents, where the solid and dashed lines show the exposure differences for households dependent upon private wells versus community water systems. The shaded area shows the corresponding confidence interval. A positive value indicates the exposure is higher for Black residents, compared to white residents. **(B): 5 km Poultry Exposure Race Difference between Hispanic and White Households, Private Well vs Community Water System**. The line shows the poultry exposure differences between Hispanic and white residents, where the solid and dashed lines show the exposure differences for households dependent upon private wells versus community water systems. The shaded area shows the corresponding confidence interval. A positive value indicates the exposure is higher for Hispanic residents, compared to white residents.

In Figs 2 and 3, we plot relative hog exposures — i.e., the difference in the hog (Fig 2) and poultry (Fig 3) exposure between each minority group and white residents and specifically, we show how these differences vary across drinking water sources. These plots suggest similar relative exposure patterns with respect to race and income. Compared across water sources, similar patterns hold for both water types, but the differences are larger for households dependent upon private wells. These exposure patterns show that increasing income does not help Black residents in avoiding CAFO exposure, suggesting that uneven distributional effects are more related to race instead of class. Specifically, Fig 2A shows that the differences in the hog exposure between Black and white households are around 500,000 SSLW for households with private wells and around 100,000 SSLW for those with community water systems, and such exposure differences are similar across income. Fig 2B shows that the differences between Hispanic and white households are larger for low-income households, especially for households with private wells. For example, for households with income below $40,000, the exposure differences could go up to 1,500,000 SSLW for households with private wells, while only 500,000 SSLW for those with community water systems. In Fig 3A, the differences in poultry exposure between Black and white residents are around 30,000 bird counts for those with private wells and close to 0 for those with community water systems. When comparing between Hispanic and white residents (Fig 3B), the differences are slightly larger for residents with private wells and the differences are between 20,000 and 40,000 bird counts for low-income households. Such exposure patterns are even more significant in major agricultural-producing counties (see Figs S26 and S27 in S1 File).

**Fig 4:**
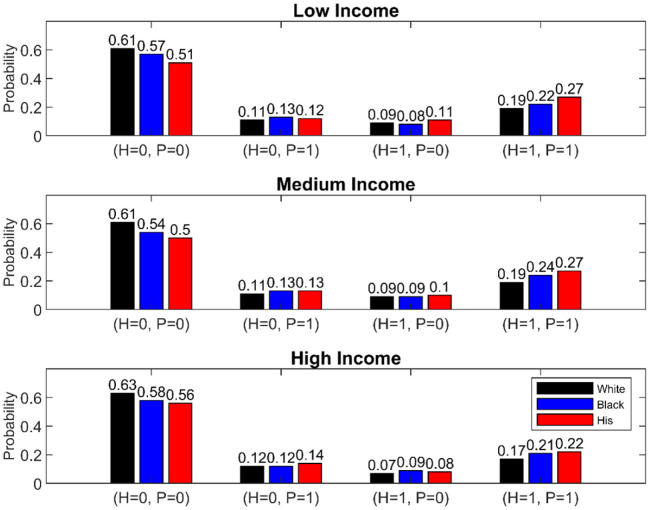
Predicted Probability (Hogs, Poultry), 5 km. Four predicted join probabilities from bivariate probit models: P(Hogs=0, Poultry=0), P(Hogs=0, Poultry=1), P(Hogs=1, Poultry=0), and P(Hogs=1, Poultry=1). In the bivariate probit model, we define outcome as 1 if the CAFO exposure is positive and 0 otherwise. The independent variables are race by income dummies. The low-income group includes households with income below $35,000, median income group includes household with income between $35,000 and $100,00, and high-income group includes households with income above $100,000.

We then conduct a bivariate probit model and examine the joint probability of CAFO exposure — i.e., being exposed to neither, either, or both hog and poultry operations (see Materials and methods). The predicted joint probabilities for 5 km analyses are plotted in Fig 4 and results for 3 km and 4 km analyses are in the supplementary information (see Tables S5 and S6 in S1 File). The results show that compared to being exposed to a single CAFO type (i.e., being exposed only to hogs or only to poultry), all race groups have higher probabilities of being exposed to both hogs and poultry, but such probabilities are higher for Black and Hispanic residents. For example, the probability of being exposed only to hogs, or only to poultry is around 0.1 across race and income groups, but the probabilities of being exposed to both hogs and poultry increase to 0.19, 0.22, and 0.27 for white, Black, and Hispanic residents. We conduct additional analyses testing equality of predicted probabilities (see Tables S8-S10 in S1 File). The tests compare whether the coefficients of minorities are larger than white and compare within income groups. Results show minorities are statistically more likely to be exposed to both hogs and poultry and that probabilities are larger for Hispanics compared to Black residents.

## Discussion

We built a data set that includes detailed information on the location of both swine and poultry CAFOs combined with household-level demographics and water source information. Our study is descriptive in nature and documents racial and income disparities in CAFO exposure. Though it does not investigate the causal aspects of the disparities in CAFO exposure, we document racial disparities in exposure that cannot be attributed to class-based characteristics. Our results are consistent with previous studies examining the relationship between race, class, and exposure to hog CAFOs in eastern North Carolina, which also found that minorities and low-income people are more likely to be exposed to hogs. Given that eastern NC also has a high concentration of poultry CAFOs, we conduct additional analyses of these operations and find similar exposure patterns.

With the detailed demographic micro-data, we are able to examine how race interacts with income in determining exposure to the harms of CAFOs. Since income could create constraints on opportunities for health care and nutritional options, the disparities in exposure across income could further amplify the health impacts of the disparities in CAFO exposure. Our results show that while increasing income helps Hispanic and white residents avoid exposure, it does not similarly help Black residents, suggesting that inequitable exposure for this group may be driven by factors associated with race instead of class. In particular, the observed exposure pattern for Black residents (as detailed in Results) is markedly different from what we observe in white and Hispanic populations, where increased income is more consistently correlated with reduced exposure.

Crucially, the absolute fall in exposure levels for Black residents at higher income brackets is not the most significant finding. Rather, it is the rate of this decline compared to white residents that demands attention. For Black residents, the decrease in exposure is slower than that experienced by their white counterparts. This indicates that the benefits of increasing income to mitigate exposure are not equally distributed across racial lines. In contrast, Hispanic residents, similar to white residents, seem to benefit more consistently from increased income in terms of reduced exposure. This suggests that while income elevation might be an effective strategy to reduce exposure disparities for Hispanic and white populations, it does not address the underlying issues that contribute to the persistent disparities faced by Black residents. Therefore, our analysis suggests that the inequitable exposure experienced by Black residents may be more deeply rooted in factors associated with race than class. This distinction is critical because it implies that policies aimed solely at economic upliftment may be insufficient to address the racial disparities in exposure.

Nonetheless, our study could be expanded in the following ways. First, according to U.S. Census, Native Americans make up a large portion of population in some areas of eastern NC, such as 41% in Robeson County. However, with the current micro-level demographic data and race imputation method [20], we are not able to investigate any environmental and equity problems associated with Native Americans in NC. These Native Americans may be assigned in incorrect racial categories and our results would be biased. According to ACS 2012–2016, there are three counties in NC with more than 10% Native American: Robeson, Scotland, and Swain. We drop East NC counties (Robeson and Scotland) as a robustness check. Around 73,000 households live in those 2 counties in our data sample. Results (see Figs S28-S30 in S1 File) and conclusions are similar compared to the main specifications but the exposure gaps between Hispanics and white households are slightly larger (around 500,000 SSLW and 20,000 bird counts) for households with private wells and income between $0 and $40,000.

Second, we rely on data that provide the location of CAFOs themselves, but we do not have data on the location of sprayfields used by the farms for waste application. Sprayfields may be an important additional route of exposure for households (see SI.File on “Environmental and health impacts of CAFOs.”) and may impose additional water contamination concerns to local communities. Another limitation is our measures of exposure which are based on proximity but do not account for wind direction. In future work, it would be interesting to collect detailed weather information and examine whether households living downwind of CAFOs face higher exposure. Because air pollution exposures are affected by weather patterns, households with CAFOs located upwind or downwind may experience differential health exposures and odor levels. For example, one study [21] found the negative effects are largest for houses located downwind of the operations. These limitations associated with sprayfields and wind direction could lead to measurement error in our exposure measures, creating attenuation bias that would bias our measures of exposure differences toward zero.

Additionally, recent developments in utilizing biogas, supported by initiatives like the Inflation Reduction Act, offer potential for reducing some externalities associated with CAFO lagoons. While this technology is relatively new, its adoption could significantly impact the environmental footprint of these operations, including the creation of externalities in biogas pipelines [22]. Finally, while a moratorium on hog CAFO construction has been in place since 1997, poultry CAFOs have continued to be built. Further research could therefore explore the relative importance of facility siting versus residential sorting in determining observed disparities in exposure for these operations in particular. This could help inform regulatory and policy strategies for redressing historically disproportionate exposures by race as well as inform future siting decision criteria to better address environmental injustices related to CAFOs. Another similar concern is that some hog farms may exit after 2014, and it would be interesting to see how such dynamics could affect exposure patterns in future work.

## Materials and methods

This section describes the data sources and methods used to generate the data analyzed in this paper. We analyze data on the location of hog farms provided by the North Carolina Department of Environmental Quality (DEQ) and the location of poultry farms provided by the Environmental Working Group and Waterkeeper Alliance. We accessed the hog farm data on 22/01/2020. Because there is a moratorium on hog farm entry (since 1997) and the hog farm data provide permit information, the hog farm dataset provides location information in 2014, which is the year we studied. The hog farm data provide information on the location, animal counts, number of lagoons, and growth phase of the hogs (e.g., “wean to feeder’‘ and “feeder to finish’‘) of each farm. Different growth phases of hogs have varying impacts on the environment, primarily due to differences in waste production. Smaller, younger hogs produce less waste compared to larger, older hogs. Simply using animal counts to estimate environmental impact can be misleading, as it does not account for the varying waste production across different growth stages. Steady state live weight (SSLW) takes into account both the number of animals and their respective weights, which correlate more directly with the amount of waste produced. Therefore, it provides a more comprehensive and accurate measure of potential environmental impact, particularly regarding feces and urine production. Because CAFOs are generally permitted with “allowable counts” of animals, we assume that the permitted SSLW for hog farms equals the average weight of a hog at the final permitted growth stage (i.e., “finished” weight in a feeder-to-finish operation) multiplied by the allowable count in a permit. So, the SSLW for a feeder-to-finish farm with an allowable count of 3,000 (which would meet the regulatory definition of a “large CAFO” because it has more than 2,500 swine weighing over 55 pounds) where the average finish weight of a hog is 135 pounds would be calculated as follows:


$$\begin{array}{c} \text{3000 }hogs\times \text{ 135}\frac{pounds}{hogs}=\text{ 405},\text{000 }pounds \end{array}\;$$ (1)

This number represents the total potential weight of the hog population on the farm and is a key metric in comparing the potential environmental impact of hog farms that focus on differing growth stages.

Poultry farm data are accessed under an agreement with Environmental Working Group (EWG) and were collected in 2014. We accessed the poultry farm data on 07/06/2017. We refer anyone who is interested in these data to EWG (https://www.ewg.org) for access request. The data include information on the location of poultry feeding operations as well as estimated bird counts for each site [23]. We use total bird counts as a summary measure of pollution produced by poultry farms.

Demographic data describing household residents come from the InfoUSA Residential Historical Files maintained by Data Axle and were accessed under a purchase agreement with Duke University. Data are available for researchers who purchase the data or otherwise meet the criteria for access. We accessed the data on 25/09/2020 and used demographic information from 2014. We include the physical address, household race, and household income in the analysis. We use the R package “wru” to impute individual race/ethnicity. The package applies Bayes’ Rule and uses the individual’s last name and geo-location (i.e., census block and the census data were accessed on 25/09/2020) to compute the posterior probability of each racial category (see SI.File on “ InfoUSA demographic data.” for definitions of race categories) of any given household. Each individual in our study is assigned to the race with the highest probability. The methods that we used to impute the race/ethnicity are described in [20]. This study was approved by the Duke Campus IRB (Protocol # 2020−0384 and 2025−0024, “Neighborhood Exposure and Proximity to CAFOs”) and UW Madison IRB (Protocol # 2025−0138). Informed consent is not applicable in our study as the collections of the InfoUSA dataset did not involve interaction with any participants.

The water service area boundaries for 532 community water suppliers in North Carolina are maintained by DEQ and were updated in 2019 by a team of students at Duke University [24]. We relied on spatial boundaries data provided by the Duke team and the data were accessed on 16/05/2020. The analytic dataset was created on 25/09/2020, when the water system data and demographic data became available. While some datasets used in this study had been accessed for prior research projects, they were not assembled into the analytic dataset for this study until 25/09/2020.

To calculate the exposure of households to CAFOs, we draw a buffer around each house and calculate the aggregated SSLW and total bird counts within that buffer. Combing demographic data with CAFO information provides an exposure picture in 2014. For all of the analyses, hog exposure is reported in units of 1 million SSLW and poultry exposure is reported in units of 100,000 bird counts. These units are similar sizes to large CAFOs. Because studies show significant correlation between exposure to CAFOs and negative health results at 3 km — e.g., increased stress, respiratory symptoms, and acute elevation of blood pressure — and since households can smell odor from hog farms that are located up to 5 km away [4,25–27], we conduct our analyses over a range of distance buffers from 3 km to 5 km. Because farms are concentrated in the eastern part of the state — an area with more low-income and people of color communities than other parts of the state — this is where we focus our analysis. Because of siting prohibitions, fewer farms are located near urban areas and urban households are systematically exposed to lower numbers of CAFOs. We therefore exclude households in the interiors of urban areas (see SI.File on “ Study area.”) in all of our analyses.

Because of the interaction of hogs and poultry, some racial groups may face higher welfare costs if they are more likely to be exposed to both hogs and poultry. To examine such disparities, we calculate the joint probability of CAFO exposure — i.e., being exposed to neither, either, or both hog and poultry operations. We allow exposure to differ for groups defined by both race and income. In particular, each household is placed into one of three race groups — low, medium and high. The low-income group is composed of households with income below $35,000 and high-income group are households with income above $100,000. We run the following bivariate probit model:


$$\begin{array}{c} \begin{array}{c} {Hogs}_{i}=\beta_{\text{0}}+\beta_{\text{1}}X_{i}+\varepsilon_{i}\\ {Poultry}_{i}=\beta_{\text{2}}+\beta_{\text{3}}X_{i}+\varepsilon_{i} \end{array} \end{array}$$ (2)

where $X_{i}$ are race by income indicator variables. Because race and income may be correlated with urban status — in particular, we find that low-income Black households are more likely to live in urban areas, ignoring urban status may therefore lead to an omitted variables problem. We include an urban area dummy to control for households who live on the peripheries of urban areas. The bivariate probit model specifies the outcomes as:


$$\begin{array}{c} Y=\left\{ \begin{array}{c} \text{1}\;if\;CAFO\;Exposure>\text{0}\\ \text{0}\;if\;CAFO\;Exposure=\text{0} \end{array}\right.\; \end{array}$$ (3)

We then calculate four joint probabilities of being exposed to hogs and/or poultry: exposure to neither hogs nor poultry: P(Hogs=0,Poultry=0), exposure to either poultry or hogs: P(Hogs=0,Poultry=1) and P(Hogs=1,Poultry=0), and exposure to both hogs and poultry: P(Hogs=1,Poultry=1).

## Supporting information

S1 File Supplementary information for “The equity implications of exposure to industrial livestock operations”. This file includes supplementary text, Figs S1-S30, and Tables S1-S10.
(DOCX)
