# Supplementary material for: The equity implications of exposure to industrial livestock operations
Source: PLoS One. 2026 Feb 19;21(2):e0342552. doi: 10.1371/journal.pone.0342552 (PMC12919807; doi:10.1371/journal.pone.0342552)
Supplement: S1 File — This file includes supplementary text, Figs S1-S30, and Tables S1-S10. (DOCX) [file pone.0342552.s001.docx]

# Supporting Information for

The equity implications of exposure to industrial livestock operations

Author list:

Kay Jowers

Yu Ma

Christopher Timmins

Corresponding author: Yu Ma

Email: [*may3@ornl.gov*](mailto:may3@ornl.gov)

**This file includes:**

Supplementary text

Figures S1 to S30

Tables S1 to S10

SI References

**Supporting Information**

## **Environmental and health impacts of CAFOs.**

CAFOs detrimentally impact the environment as well as the health and well-being of those who live nearby. For example, CAFOs could affect surface water quality and increasing intensity could increase the levels of nutrients in the surface water [29]. Such a high concentration of animals in eastern NC is of concern because of the amount of waste generated and the responsibilities placed on growers who are already operating at tight margins set by the integrators [1]. Swine manure is collected within pits beneath the building holding animals and is flushed regularly into open-air lagoons. The waste contains contaminants including nutrients, pathogens, heavy metals, and biochemical oxygen-demanding materials. The liquid waste is then ground-applied as fertilizer for crops onto “sprayfields” [2]. When CAFOs exist in close proximity to one another, the concentration of sprayfields can overwhelm the capacity of the land to treat the nutrients and other biological contaminants, such as fecal coliform, in the waste, causing those substances to leach into groundwater or run off [3][2] into nearby rivers, streams, or adjacent properties. In addition, lagoon storage systems that are not properly lined or maintained can leach into groundwater or contaminate surface waters during extreme weather events that lead to accidental releases and catastrophic ruptures [4][5]. Even the gradual migration of water nutrients from facilities to surface water can cause excessive growth of phytoplankton, which can lead to a decrease in the dissolved oxygen in nearby rivers and streams, resulting in fish kills [5].

These operations also emit air pollutants and odor [6][7][8][9][10]. Hog CAFOs emit hydrogen sulfide ($H_{2}S$), endotoxins, and particulate matter (${PM}_{10}$, ${PM}_{2.5}$), all of which are known to impact people's health [11][8][10]. Hog CAFOs also emit greenhouse gases, such as nitrous oxide ($N_{2}O$) and methane, that contribute directly to climate damage [7].

Poultry operations collect waste as dry litter, mix it with straw or other materials, and then land apply it to nearby fields or compost it [12]. Because the waste is dry, the poultry farms are not required to apply for water waste management permits and the industry is largely unregulated. Despite the dry litter system, poultry farms are associated with many of the same environmental impacts as hog farms [13][14][15][16][17].

[18] found communities located near hog CAFOs have worse health outcomes, such as higher rates of all-cause mortality, infant mortality, mortality from anemia, kidney disease, and higher rates of emergency department visits and hospital admissions. Air pollutants are found to be correlated with physical symptoms, such as difficulty in breathing, sore throat, chest tightness, and nausea [8]. The airborne dust particles and fungi emitted by hog CAFOs can lodge deep within lung tissue [4][11]. The odors generated by CAFOs can cause increases in acute blood pressure and contribute to development of chronic hypertension [10]. These waste management practices can lead infectious agents to mutate and create novel disease strains, increase the resistance to antibiotics, and infect susceptible host populations [4][5]). Living next to an industrial farming facility can also have an impact on overall well-being. People living near hog farms find themselves less frequently able to open their windows or go outside [19][6] and are more likely to report feeling stress [6].

The concentration of hog farming in the eastern area of North Carolina, where many communities of color and low-income populations reside, raises environmental justice concerns. [20] found that a higher proportion of Blacks, Hispanics, and Native Americans live within 3 miles of an industrial hog operations when compared to non-Hispanic whites. Another study [3] found the hog farming facilities are highly concentrated in areas with higher dependence on wells as water sources and higher poverty level. Research also shows that the communities in close proximity to high concentrations of CAFOs also suffer from low wages, lack of access to medical care, and poor nutritional options [3].

Environmental justice refers to social inequalities in the distribution of natural hazards (e.g. hurricanes and floods) and examines intra-ethnic diversity in patterns of environmental injustice [21][22].

## **Regulating the industry.**

North Carolina temporarily halted the construction and/or expansion of hog CAFOs in 1997 following catastrophic failures of lagoons during 1996's Hurricane Fran and the release of studies indicating the concentration of farms in poor communities of color [23]. Over the next several years, more studies of the impacts were published [24][3][19] and another disastrous hurricane, Hurricane Floyd, caused flooding in eastern NC that ruptured lagoons and killed many hogs. To avoid litigation, Smithfield Foods, the largest integrator in the state, entered into an agreement with the state in which the company would finance research on the impacts of their practices, research to identify waste management systems that were “environmentally superior” to the lagoon and sprayfield system, and the implementation within three years of any system that is found environmentally superior to the lagoon and sprayfield system and is economically feasible (NC Attorney [25]). The environmental performance standards laid out in the Agreement, which expires in 2025, were incorporated into state law in 2007 when the NC General Assembly permanently prohibited any new or expanded hog CAFOs based on the lagoon and sprayfield system (NC Gen Stat. 143-215.10I).

## **NC community water system data.**

The community water system data is managed by the NC Department of Environmental Quality (DEQ) Division of Water Resources. A team of students at Duke University digitized community water systems (CWS) maps in partnership with NC DEQ (see S1 Fig). The goal of this effort was to digitize all CWSs that are either publicly owned or serving over 3,000 people by using the maps provided by CWS and Census TIGER lines of boundaries of incorporated municipalities. 70% of CWSs provide maps to digitize. For the 17% of CWSs that are municipal systems but have no maps to digitize, the team adapted the boundaries of the incorporated municipalities. 13% of CWSs are not included in the final data because they neither provided a map nor are they municipal systems. The final data contains service area boundaries for 532 CWSs in NC [26].


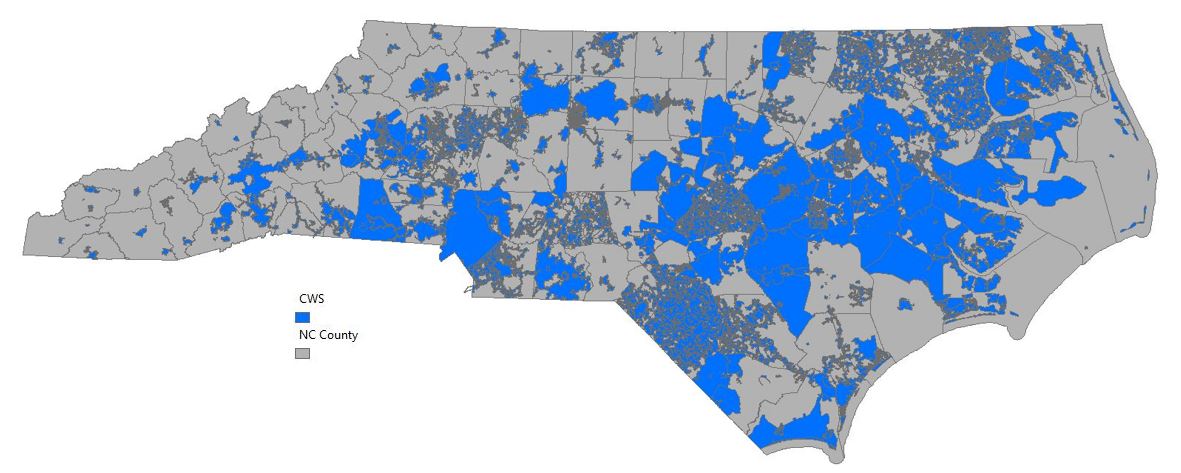


**S1 Fig. NC Community Water System Map.**

Digitized community water systems (CWS) maps in partnership with NC DEQ. Gray areas are NC counties. Blue regions are areas served by community water systems and the rest of areas are on private wells. The map of NC counties is obtained from US Census Bureau (<https://www.census.gov/en.html>) (public domain).

## **InfoUSA demographic data.**

Demographic data describing household residents come from the InfoUSA Residential Historical Files, which are maintained by Data Axle and were accessed under a purchase agreement with Duke University. These data include household income and race of the household head. We exclude households located in the coastal area, defined as those who live within 5 miles of the eastern NC coastlines. Our data show almost 99% coastal households are white. We also exclude households in urban areas in the analyses. We include the physical address, household race, and household income in the analysis.

We use R package “wru” to impute individual race/ethnicity. The package utilizes Bayes’ Rule and uses individual surname and geo-location (e.g., census block) to compute the posterior probability of each racial category for any given individual. The package implements methods described in [27]. The method used Florida voter files for race prediction. According to Florida voter files, “Hispanic” refers to individuals who identify as having origins from Spanish-speaking countries or cultures. By law, Florida asks voters to identify their race/ethnicity. According to the race codes listed in Florida Division of Elections, Hispanics can include Black and white households and there are separate categories for “white, not Hispanic” and “Black, not Hispanic”. Therefore, based on the race code, the definitions are mutually exclusive.

S1 Table shows the summary statistics for race, income, and household water source for InfoUSA households in eastern NC. S2 Table shows the summary statistics for CAFO exposure: aggregated hogs and poultry exposure within 5km and percentage of households being exposed to hogs and poultry within 3/4/5/km buffer. All analyses are separated into all households, households on private wells, and households on community water. In S3 Table, we provide additional summary descriptives by focusing on households with positive exposure to hogs or poultry and showing the total number of observations, categorized by race, income, and water supply source.

**S1 Table. InfoUSA Summary Statistics - Race and Income**

|  | All Households | Private Wells | Community Water System |
| --- | --- | --- | --- |
| Race and Income |  |  |  |
| White | 0.74 (0.44) | 0.80 (0.40) | 0.73 (0.45) |
| Black | 0.22 (0.42) | 0.16 (0.37) | 0.24 (0.42) |
| Hispanic | 0.04 (0.19) | 0.04 (0.20) | 0.04 (0.19) |
| Income | 59999 (55721) | 61977 (53006) | 59568 (56287) |
| Owner | 0.65 (0.48) | 0.72 (0.45) | 0.64 (0.48) |
| Race by Income |  |  |  |
| White Low Income | 0.26 (0.44) | 0.27 (0.44) | 0.26 (0.44) |
| White Median Income | 0.33 (0.47) | 0.37 (0.48) | 0.32 (0.47) |
| White High Income | 0.16 (0.36) | 0.16 (0.37) | 0.15 (0.36) |
| Black Low Income | 0.14 (0.34) | 0.09 (0.28) | 0.15 (0.36) |
| Black Median Income | 0.069 (0.25) | 0.058 (0.23) | 0.071 (0.26) |
| Black High Income | 0.016 (0.12) | 0.014 (0.12) | 0.016 (0.13) |
| Hispanic Low Income | 0.018 (0.13) | 0.018 (0.13) | 0.017 (0.13) |
| Hispanic Median Income | 0.015 (0.12) | 0.018 (0.13) | 0.014 (0.12) |
| Hispanic High Income | 0.005 (0.07) | 0.006 (0.07) | 0.005 (0.07) |
| N | 1057216 | 189394 | 867822 |

Demographic data describing household residents come from the InfoUSA Residential Historical Files and were accessed under a use agreement with Duke University. Race and owner categories are calculated in percentage and income category is calculated in mean (with standard deviation). The low-income group includes households with income below $35,000, median income group includes household with income between $35,000 and $100,00, and high-income group includes households with income above $100,000.

**S2 Table. InfoUSA Summary Statistics - Exposure**

|  | All Households | Private Wells | Community Water System |
| --- | --- | --- | --- |
| Hogs within 5km | 0.22 (0.79) | 0.39 (1.12) | 0.18 (0.69) |
| Poultry within 5km | 0.23 (0.72) | 0.36 (0.94) | 0.20 (0.66) |
| % having hogs within 3km | 0.16 (0.37) | 0.25 (0.44) | 0.14 (0.35) |
| % having hogs within 4km | 0.23 (0.42) | 0.34 (0.47) | 0.20 (0.40) |
| % having hogs within 5km | 0.29 (0.45) | 0.41 (0.49) | 0.26 (0.44) |
| % having poultry within 3km | 0.18 (0.39) | 0.24 (0.43) | 0.17 (0.38) |
| % having poultry within 4km | 0.25 (0.43) | 0.33 (0.47) | 0.23 (0.42) |
| % having poultry within 5km | 0.32 (0.46) | 0.40 (0.49) | 0.30 (0.46) |

CAFO Exposure Summary. The summary statistics use same data as the 3/4/5km exposure regression. Data exclude households located in the coastal and urban areas. Exposure adjusted by large CAFO: 1 million SSLW, 100,000 total bird count.

**S3 Table. InfoUSA Summary Statistics - Households with 5km CAFOs**

|  | Having Hogs Within 5km | Having Poultry Within 5km |
| --- | --- | --- |
| Race and Income | | |
| White | 0.73 (0.44) | 0.72 (0.45) |
| Black | 0.22 (0.42) | 0.24 (0.42) |
| Hispanic | 0.05 (0.21) | 0.05 (0.21) |
| Income | 55782 (50795) | 57470 (49305) |
| Owner | 0.70 (0.46) | 0.70 (0.46) |
| Race by Income | | |
| White Low Income | 0.27 (0.44) | 0.25 (0.44) |
| White Median Income | 0.34 (0.47) | 0.33 (0.47) |
| White High Income | 0.12 (0.33) | 0.14 (0.34) |
| Black Low Income | 0.13 (0.34) | 0.14 (0.35) |
| Black Median Income | 0.08 (0.27) | 0.08 (0.27) |
| Black High Income | 0.02 (0.12) | 0.02 (0.12) |
| Hispanic Low Income | 0.02 (0.15) | 0.02 (0.14) |
| Hispanic Median Income | 0.02 (0.14) | 0.02 (0.14) |
| Hispanic High Income | 0.0045 (0.07) | 0.0049 (0.07) |
| % Private Wells | 0.25 (0.43) | 0.23 (0.42) |
| N | 305480 | 333919 |

Demographic data describing household residents come from the InfoUSA Residential Historical Files and were accessed under a use agreement with Duke University. This table focuses on households with hogs or poultry within 5km. Race and owner categories are calculated in percentage and income category is calculated in mean (with standard deviation). The low-income group includes households with income below $35,000, median income group includes household with income between $35,000 and $100,00, and high-income group includes households with income above $100,000.

## **Study area.**

Because farms are concentrated in the eastern part of the state -- an area with more low-income and POC communities than other parts of the state -- this is where we focus our analysis. Figs S2 and S3 show the distributions of hog and poultry farms in eastern NC. The area is decomposed into 1,855 block groups, where the darker areas indicate block groups with a higher percentage of people of color (POC). We use the same POC definition as used in [20] -- i.e., all people excluding non-Hispanic whites.


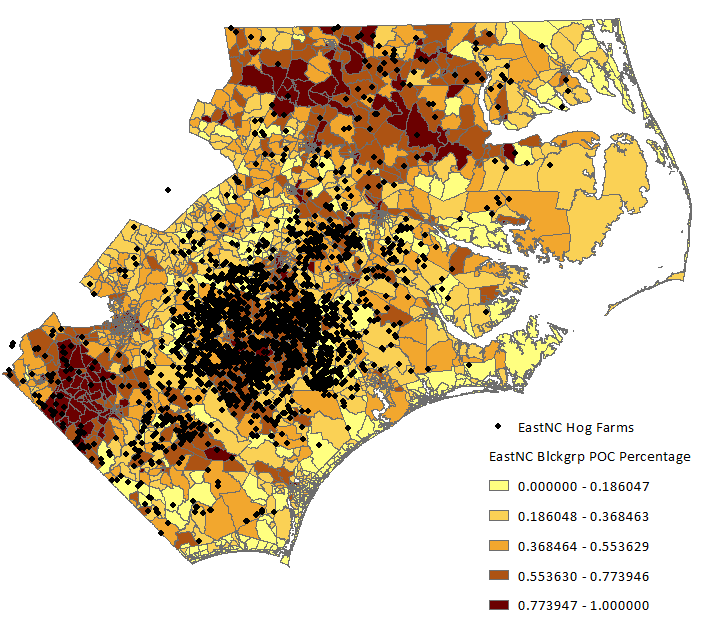


**S2 Fig. Hog Farm Locations.**

Locations of hog farms (provided by NC Department of Environmental Quality) in eastern NC. The area is decomposed into 1,855 block groups, where the darker areas are those with a higher percentage of people of color. The map of NC block groups is obtained from US Census Bureau (<https://www.census.gov/en.html>) (public domain).


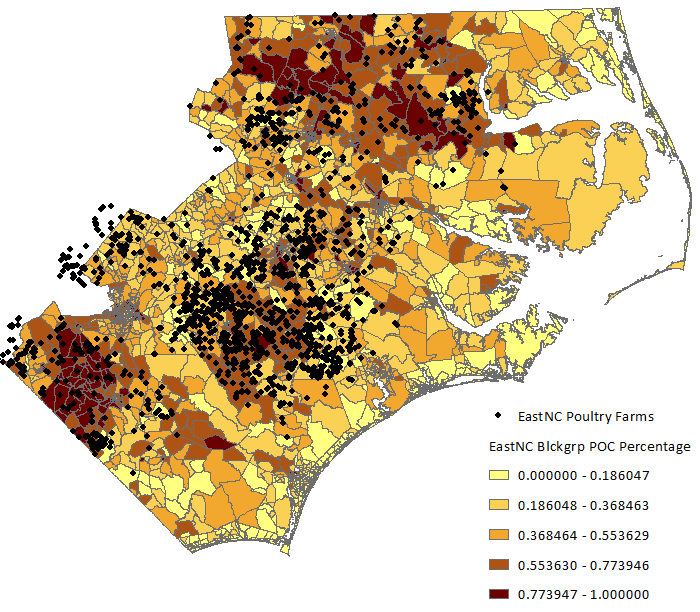


**S3 Fig. Poultry Farm Locations.**

Locations of poultry farms (provided by Environmental Working Group) in eastern NC. The area is decomposed into 1,855 block groups, where the darker areas are those with a higher percentage of people of color. The map of NC block groups is obtained from US Census Bureau (<https://www.census.gov/en.html>) (public domain).

Because households located in urban areas are systematically exposed to lower CAFOs as farms are prohibited locating in urban areas, we also exclude urban households in all of our analyses. We use 2010 Census and Urban Area designations to define urban area. Urban area is defined by both urbanized area (a densely settled core of census tracts and/or census blocks that have 50,000 or more people) and urban clusters (a densely settled core of census tracts and/or census blocks that have at least 2,500 and less than 50,000 people). Although CAFOs are not located in the urban area, some farms were sited closely to urban area boundary. Our data show the closest distances between CAFOs, and urban boundaries are 282 and 7.64 meters for hogs and poultry, respectively. Households living on the edge of an urban boundary could therefore be exposed to CAFOs, so we consider the urban households to be the ones located in the center of urban area. S4 Fig is one example using a 3km buffer. We draw a 3km interior buffer of urban area, the households located in the interior buffer (the triangles) are defined as urban households and the households outside the interior buffer (the dots) are defined as “urban buffer” households and are included in the analyses. We draw the corresponding distance buffers for each distance specification.


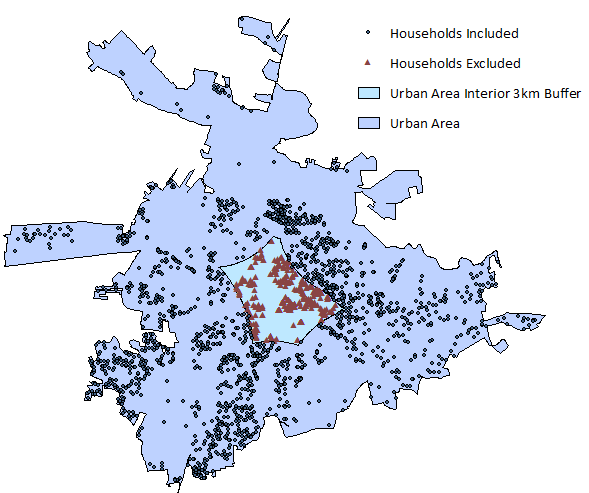


**S4 Fig. Urban Area Definition (3km example).**

Urban households are excluded in all of our analyses. We begin by considering the set of both urbanized areas (densely settled cores of census tracts and/or census blocks that have 50,000 or more people) and urban clusters (densely settled cores of census tracts and/or census blocks that have at least 2,500 and less than 50,000 people). We draw a 3km interior buffer in each of these areas and households located in the interior buffer (the triangles) are defined as urban households and are excluded in our analyses. We use the same methods for 4km and 5km analyses. The map of urban areas is obtained from US Census Bureau (<https://www.census.gov/en.html>) (public domain).

## **Steady state live weight (SSLW) calculation.**

Because different growth phases of hogs have varying impacts on the environment, primarily due to differences in waste production. Simply using animal counts to estimate the pollution exposure can be misleading. Instead, we construct the steady state live weight (SSLW) variable that considers both the growth stage and number of animals. Given different weights (see S4 Table) of a hog in each growth stage, we calculate SSLW by multiplying the number of animals with average weight of a hog at its respective weight for every farm.

**S4 Table. Average weights in each regulated activity**

| Growth Stage | Average weights |
| --- | --- |
| Swine – wean to feeder | 30 |
| Swine – feeder to finish | 135 |
| Swine – wean to finish | 135 |
| Swine – gilts | 150 |
| Swine – boar/stud | 400 |
| Swine – farrow to wean | 433 |
| Swine – farrow to feeder | 522 |
| Swine – farrow to finish | 1417 |

Average weights in each growth stage.

Source: NRCS Tech Std 633 for Waste Utilization Manure tables.

## **Single CAFO exposure.**

We use animal farm data (provided by NC Department of Environmental Quality and Environment Water Group) and household demographic data InfoUSA to calculate the aggregated SSLW (in 1,000,000) and bird counts (in 100,000) within household's 3km/4km^[[1]](#footnote-1)^ buffers and corresponding 95% confidence interval at each income level for each race (white, Black, and Hispanic). In Figs S5 and S6, the two panels show the hog/poultry exposure (within 3km/4km) distribution for all households (both owners and renters).


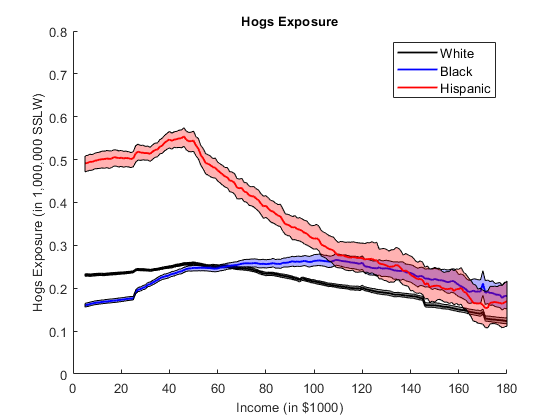


Figure (5a)


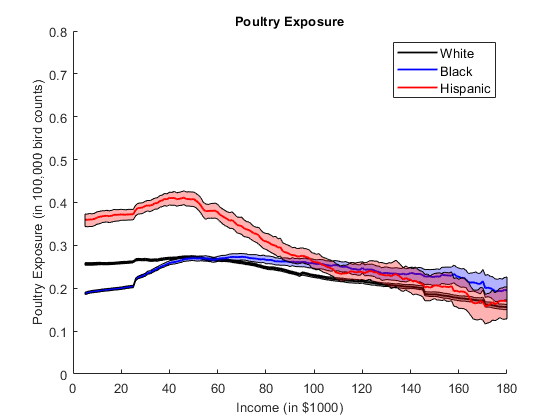


Figure (5b)

**S5 Fig. 3km Hogs and Poultry Exposure, by Race and Income.**

We use animal farm data (provided by NC Department of Environmental Quality and Environmental Working Group) and household demographic data (InfoUSA) to calculate the aggregated SSLW (in 1,000,000) and bird counts (in 100,000) within household's 3km buffer and corresponding 95% confidence interval at each income level for each race (white, Black, and Hispanic). The two panels show the hog/poultry exposure distribution for all households. The solid line shows the exposure, and shaded area shows the corresponding 95% confidence interval.


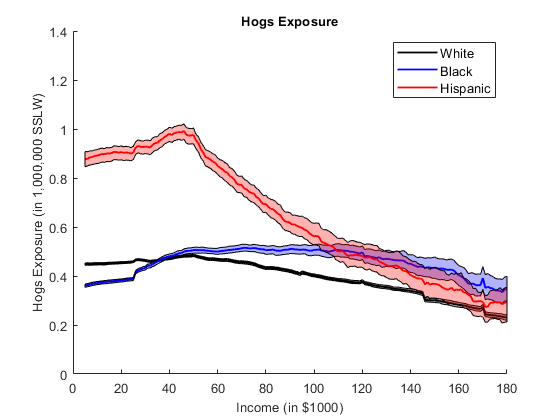


Figure (6a)


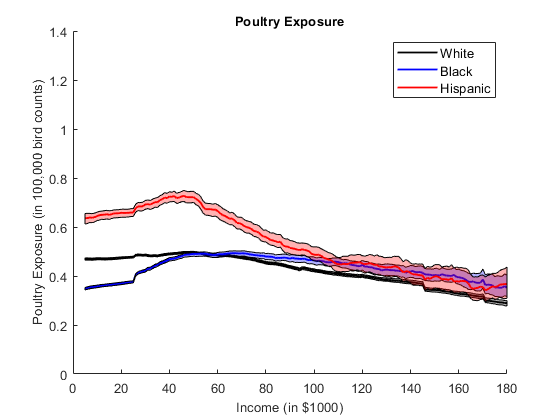


Figure (6b)

**S6 Fig. 4km Hogs and Poultry Exposure, by Race and Income.**

We use animal farm data (provided by NC Department of Environmental Quality and Environmental Working Group) and household demographic data (InfoUSA) to calculate the aggregated SSLW (in 1,000,000) and bird counts (in 100,000) within household's 4km buffer and corresponding 95% confidence interval at each income level for each race (white, Black, and Hispanic). The two panels show the hog/poultry exposure distribution for all households. The solid line shows the exposure, and shaded area shows the corresponding 95% confidence interval.

## **Single CAFO exposure, by owners and renters.**

We use animal farm data (provided by NC Department of Environmental Quality and Environment Water Group) and household demographic data InfoUSA to calculate the aggregated SSLW (in 1,000,000) and bird counts (in 100,000) within household's 3km/4km/5km (see Figs S7-S9) buffers and corresponding 95% confidence interval at each income level for each race (white, Black, and Hispanic). The four panels show the hog/poultry exposure distribution (within 3km/4km/5km) for owners and renters.


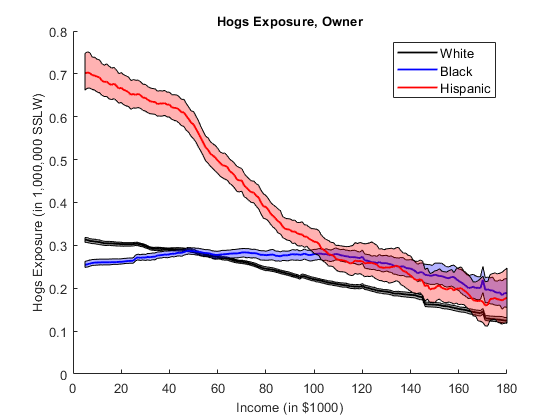


Figure (7a)

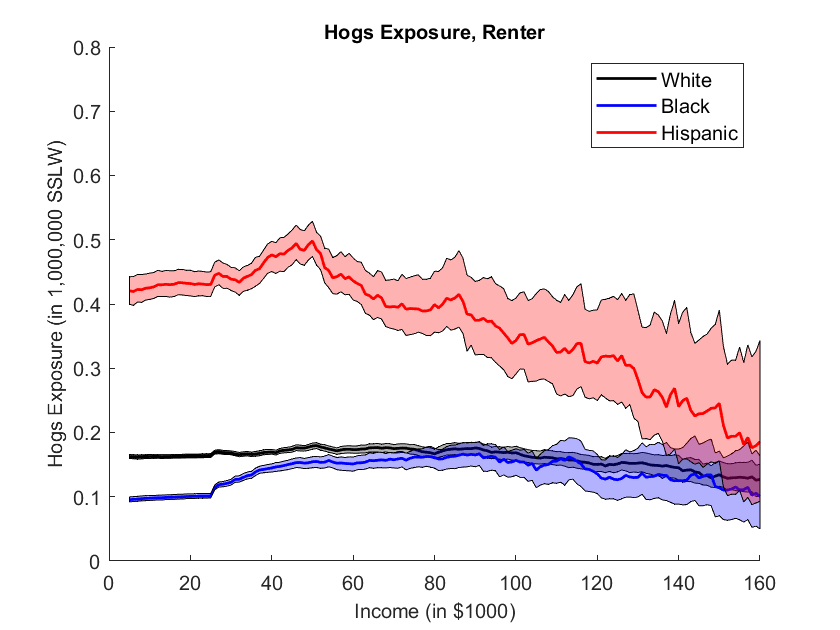


Figure (7b)


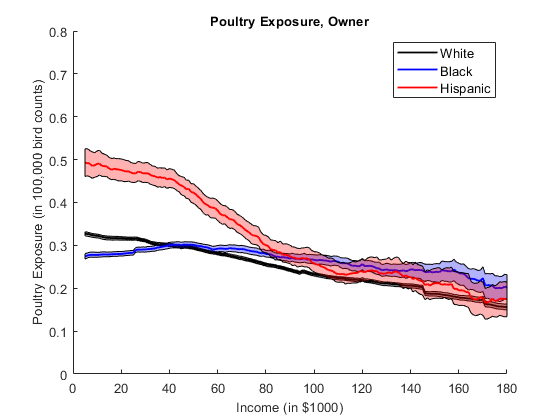


Figure (7c)


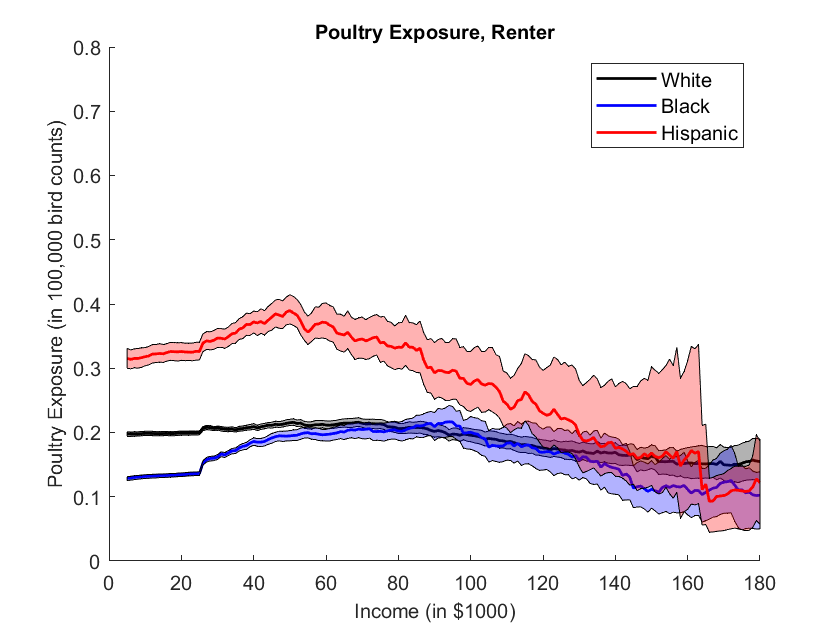


Figure (7d)

**S7 Fig. 3km Hogs and Poultry Exposure by Owner/Renter, by Race and Income.**

We use animal farm data (provided by NC Department of Environmental Quality and Environmental Working Group) and household demographic data (InfoUSA) to calculate the aggregated SSLW (in 1,000,000) and bird counts (in 100,000) within household's 3km buffer and corresponding 95% confidence interval at each income level for each race (white, Black, and Hispanic). The four panels show the hog/poultry exposure distribution for owners and renters. The solid line shows the exposure, and shaded area shows the corresponding 95% confidence interval.


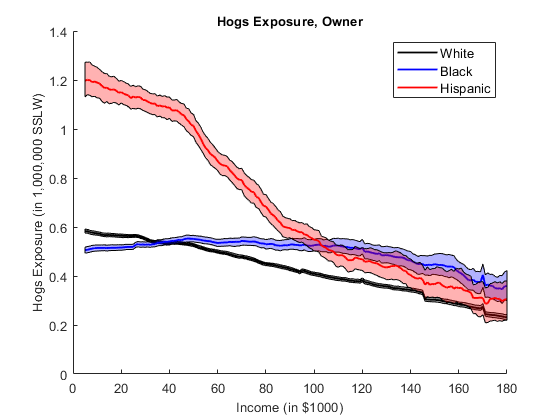


Figure (8a)


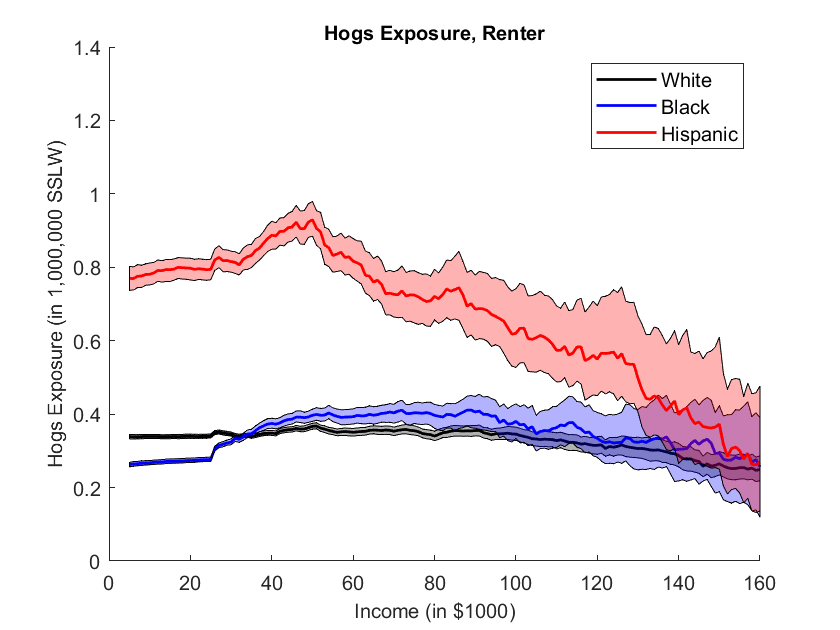


Figure (8b)


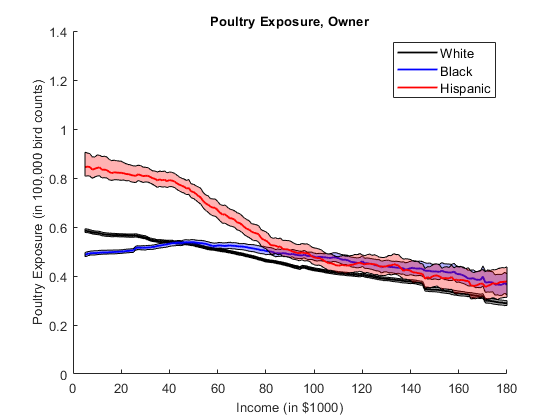


Figure (8c)


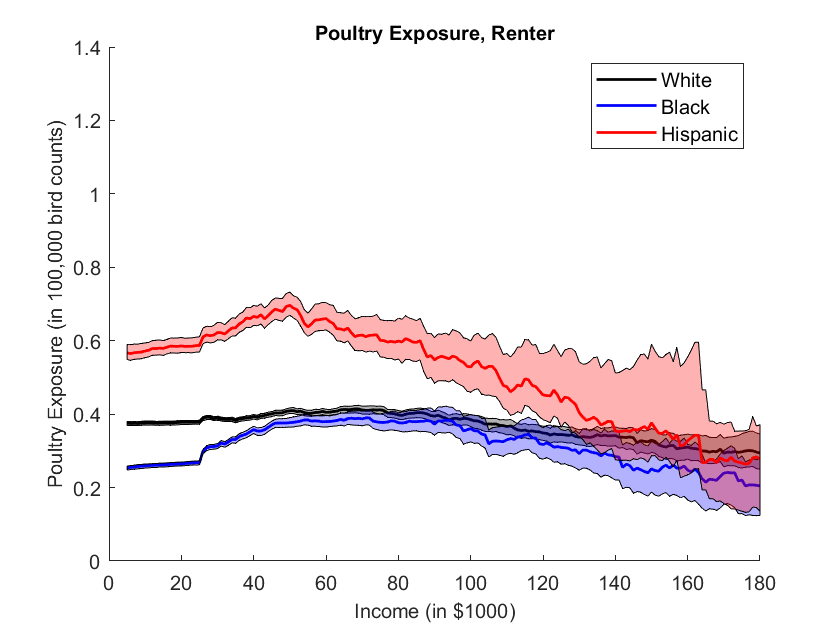


Figure (8d)

**S8 Fig. 4km Hogs and Poultry Exposure by Owner/Renter, by Race and Income.**

We use animal farm data (provided by NC Department of Environmental Quality and Environmental Working Group) and household demographic data (InfoUSA) to calculate the aggregated SSLW (in 1,000,000) and bird counts (in 100,000) within household's 4km buffer and corresponding 95% confidence interval at each income level for each race (white, Black, and Hispanic). The four panels show the hog/poultry exposure distribution for owners and renters. The solid line shows the exposure, and shaded area shows the corresponding 95% confidence interval.


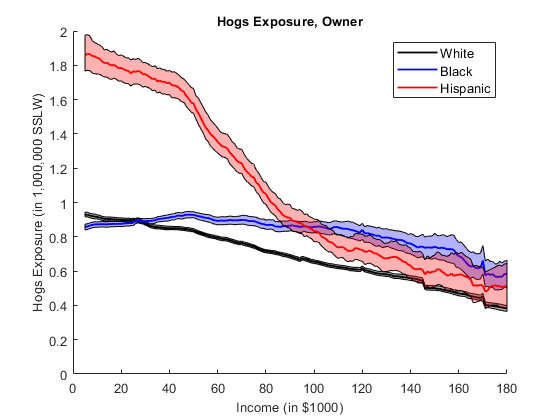


Figure (9a)


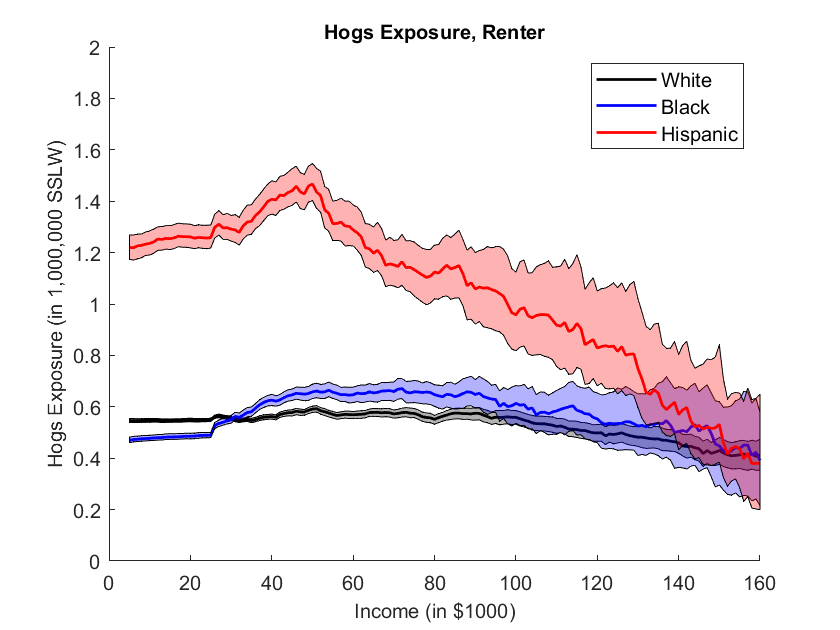


Figure (9b)


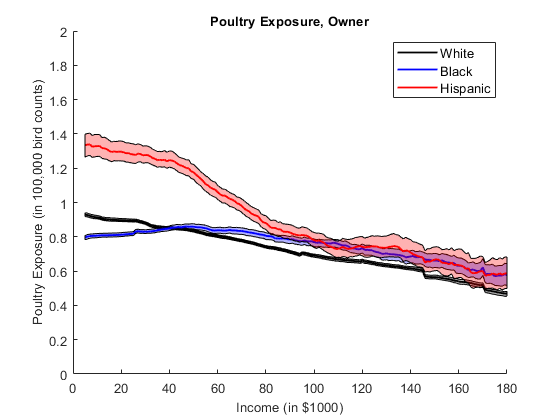


Figure (9c)


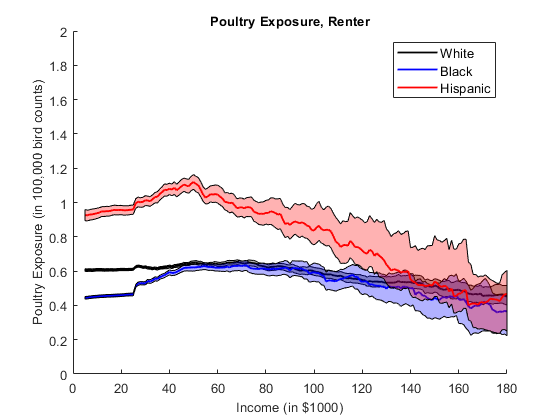


Figure (9d)

**S9 Fig. 5km Hogs and Poultry Exposure by Owner/Renter, by Race and Income.**

We use animal farm data (provided by NC Department of Environmental Quality and Environmental Working Group) and household demographic data (InfoUSA) to calculate the aggregated SSLW (in 1,000,000) and bird counts (in 100,000) within household's 5km buffer and corresponding 95% confidence interval at each income level for each race (white, Black, and Hispanic). The four panels show the hog/poultry exposure distribution for owners and renters. The solid line shows the exposure, and shaded area shows the corresponding 95% confidence interval.

## **Hog exposure differences.**

This section plots (see Figs S10-S12) the hog (in 1,000,000 SSLW) exposure difference between minority group and white for owners and renters. The solid line shows the exposure differences between Black/Hispanic and white residents and the shaded area shows the corresponding confidence interval. A positive value indicates the exposure is higher for Black/Hispanic residents, compared to white residents.


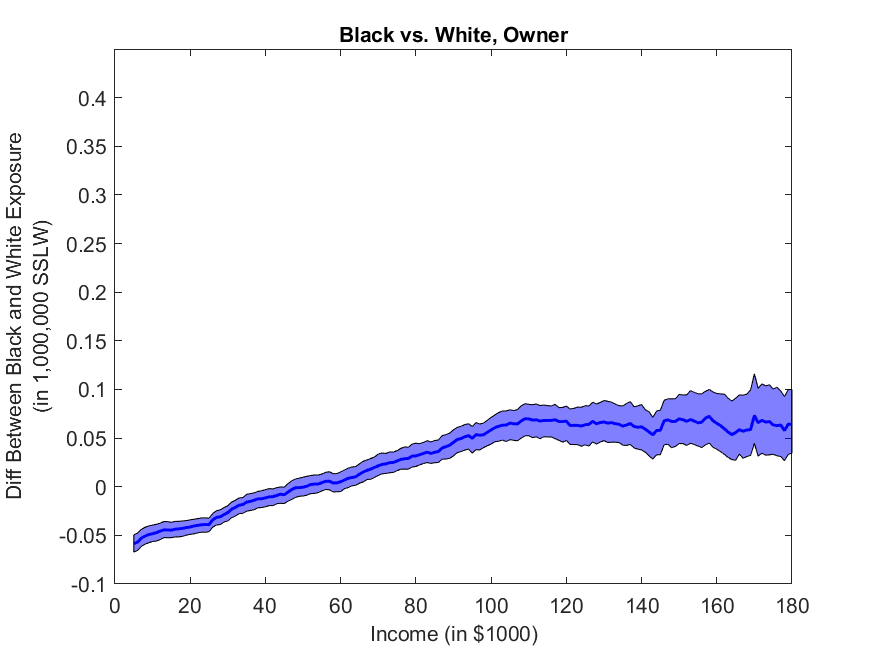


Figure (10a)


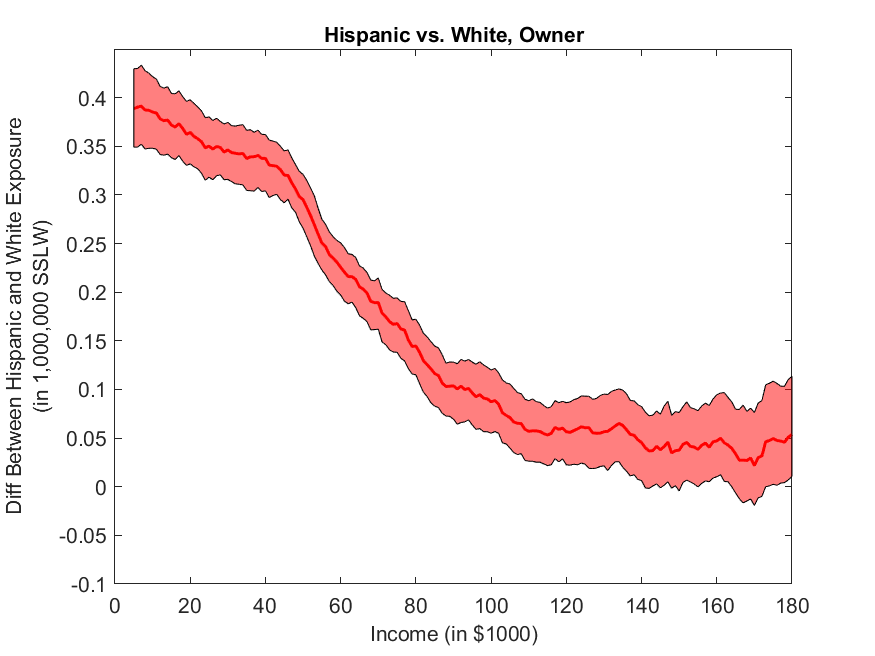


Figure (10b)


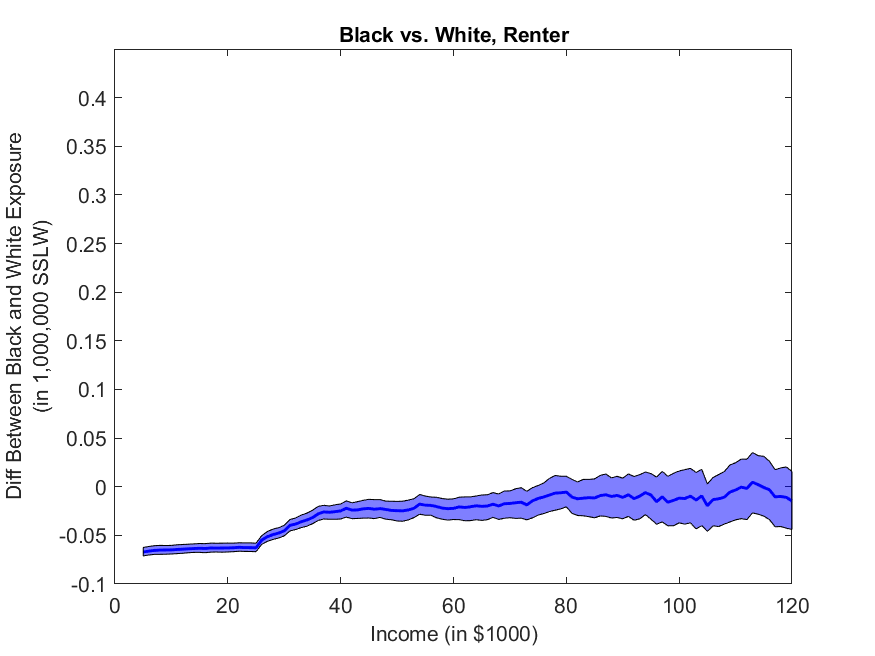


Figure (10c)


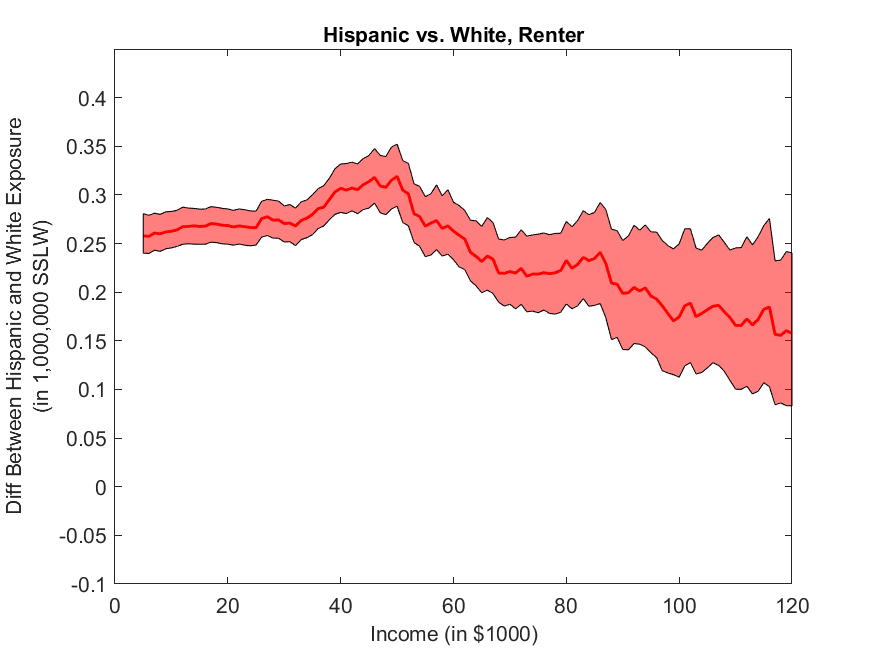


Figure (10d)

**S10 Fig. 3km Hogs Exposure Race Difference.**

We use hog farm data (provided by NC Department of Environmental Quality) and household demographic data (InfoUSA) to calculate the hog exposure (within 3km) difference (in 1,000,000 SSLW) between minority group and white for owners and renters. The solid line shows the exposure differences between Black/Hispanic and white residents. The shaded area shows the corresponding 95% confidence interval.


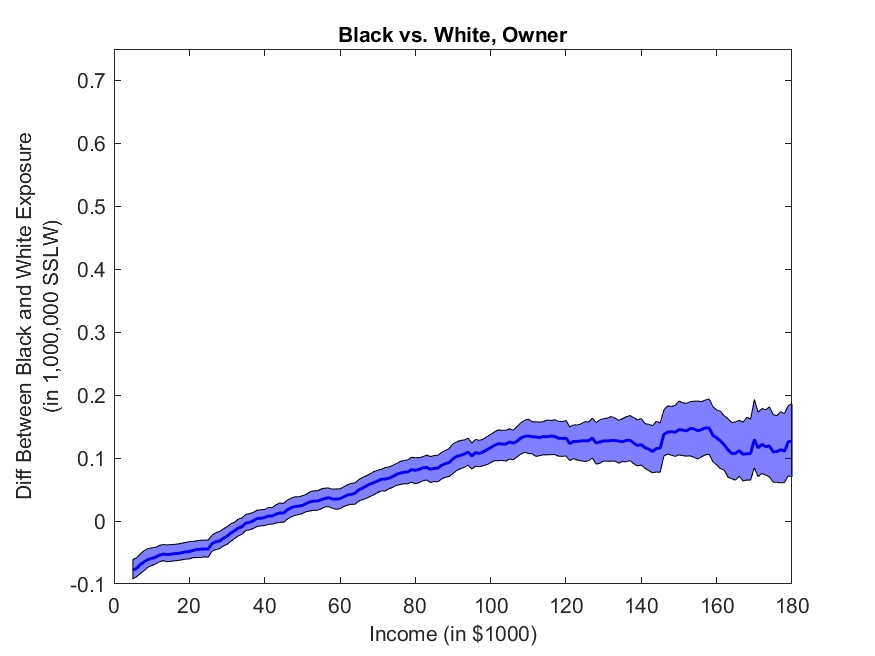


Figure (11a)


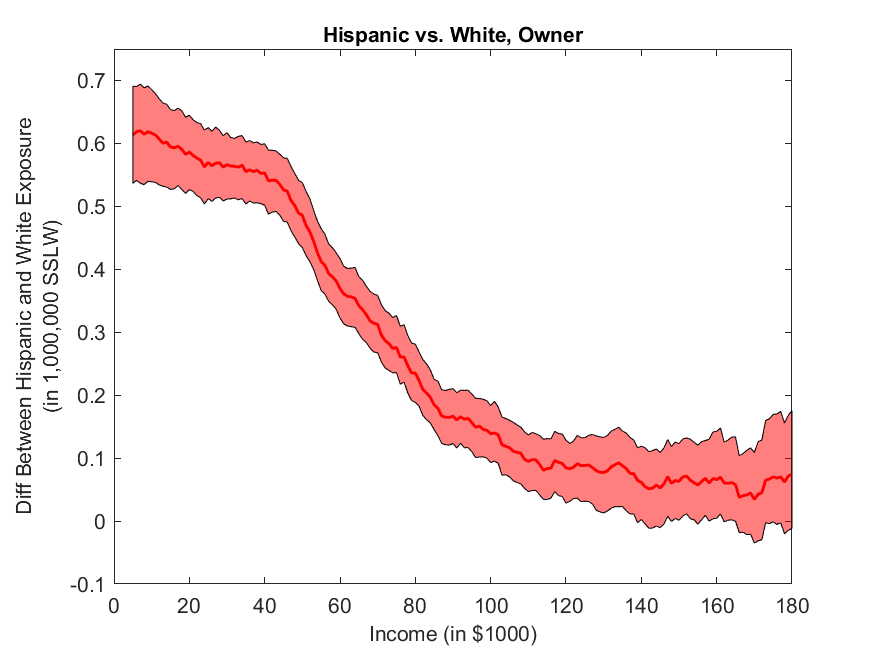


Figure (11b)


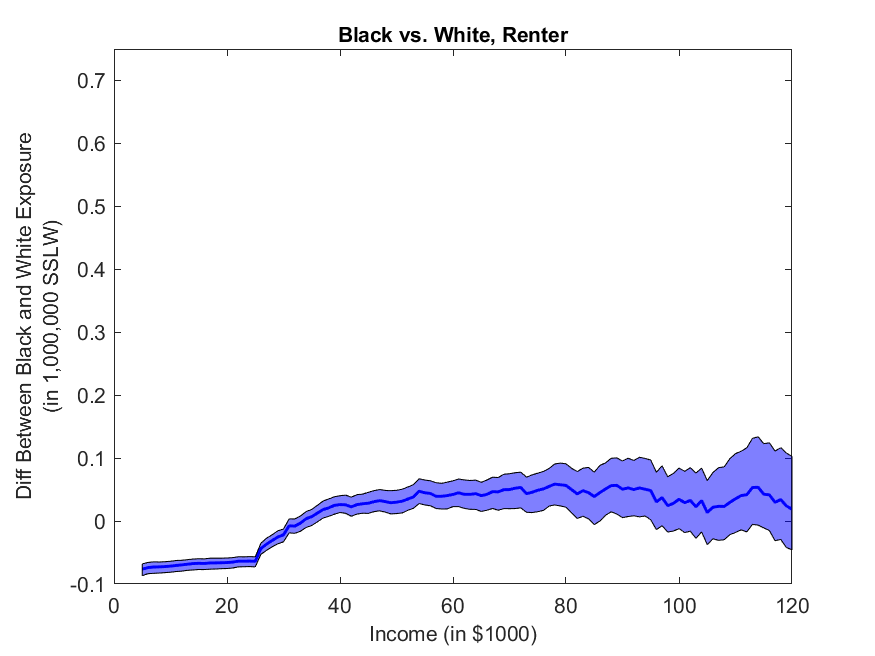


Figure (11c)


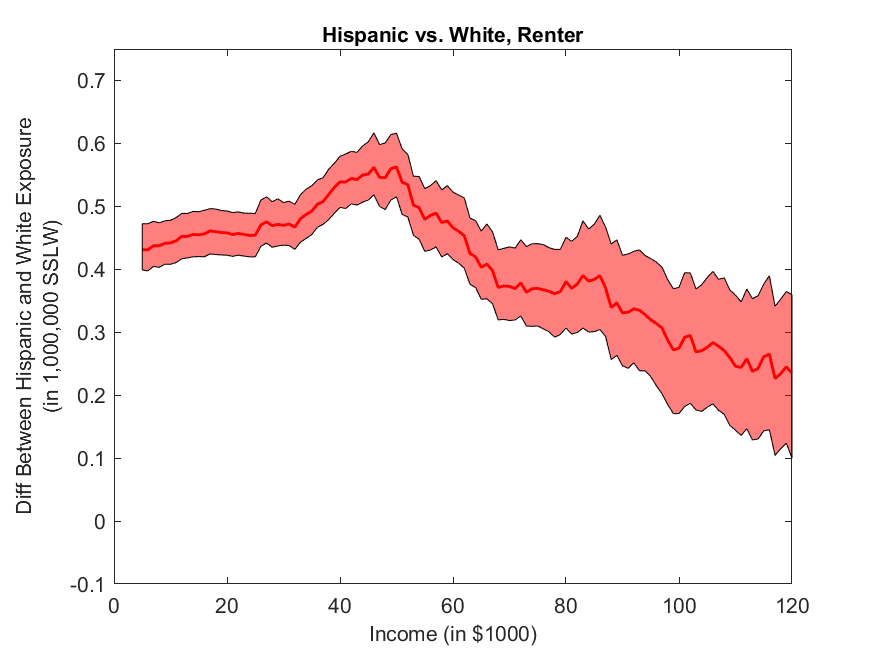


Figure (11d)

**S11 Fig. 4km Hogs Exposure Race Difference.**

We use hog farm data (provided by NC Department of Environmental Quality) and household demographic data (InfoUSA) to calculate the hog exposure (within 4km) difference (in 1,000,000 SSLW) between minority group and white for owners and renters. The solid line shows the exposure differences between Black/Hispanic and white residents. The shaded area shows the corresponding 95% confidence interval.


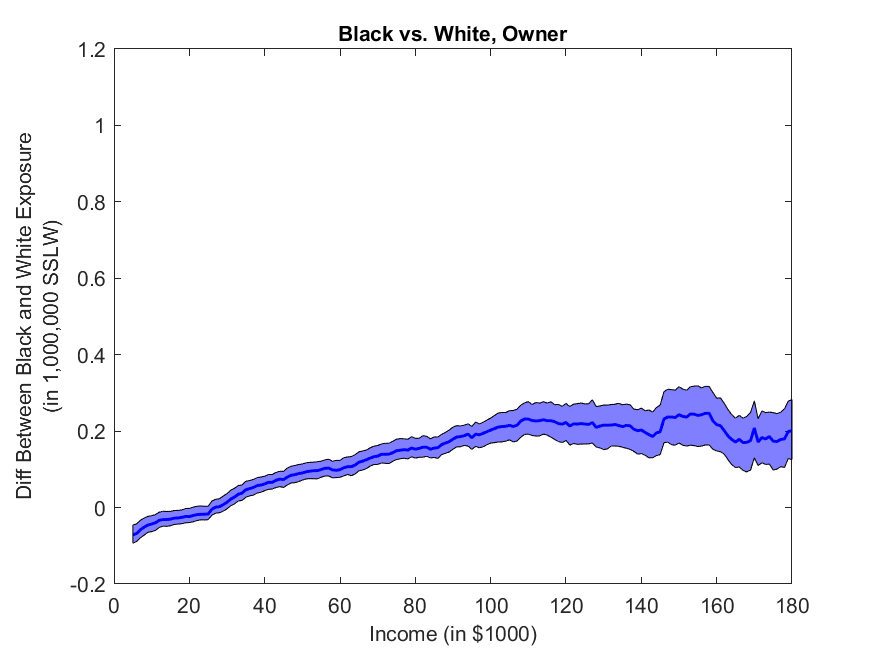


Figure (12a)


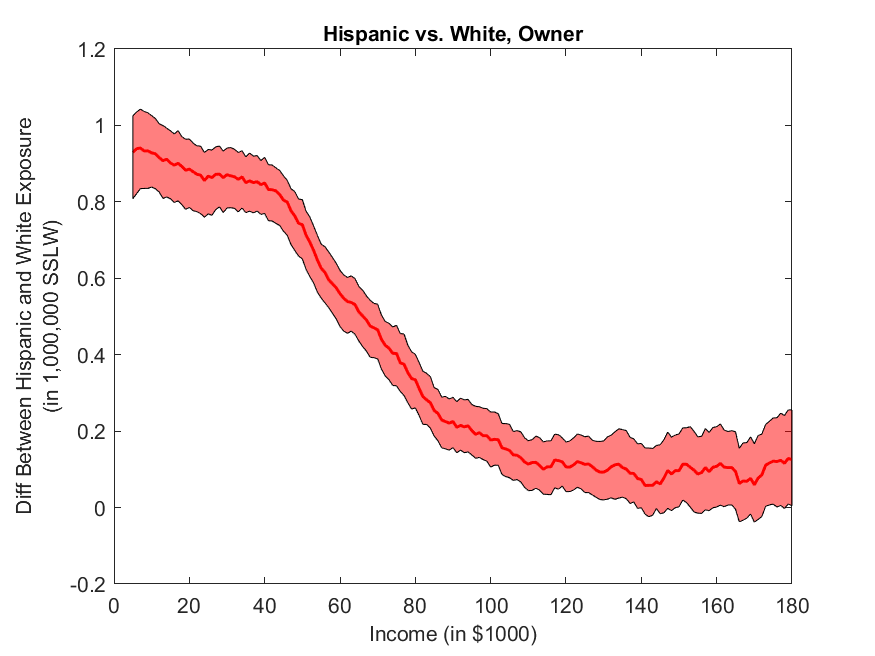


Figure (12b)


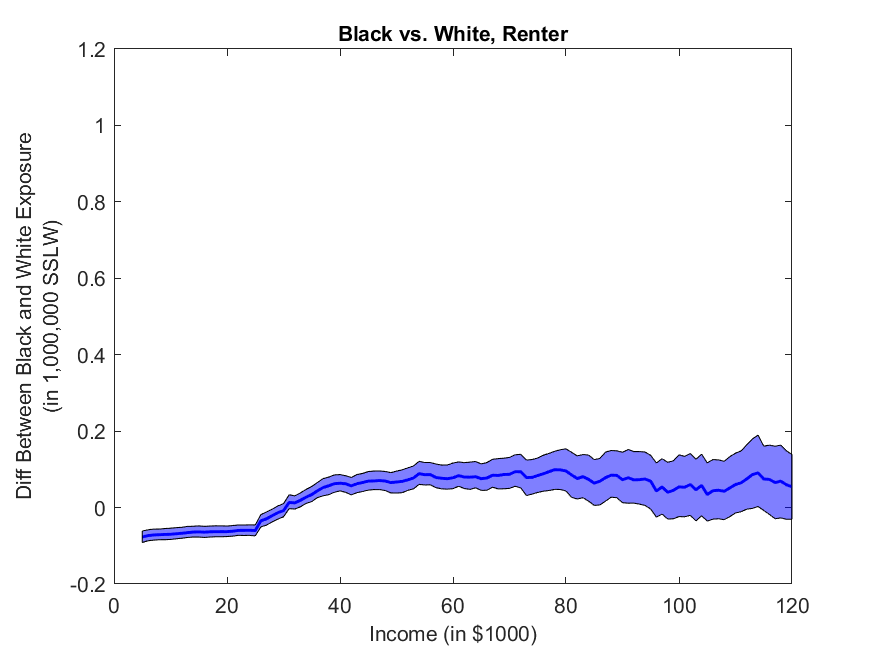


Figure (12c)


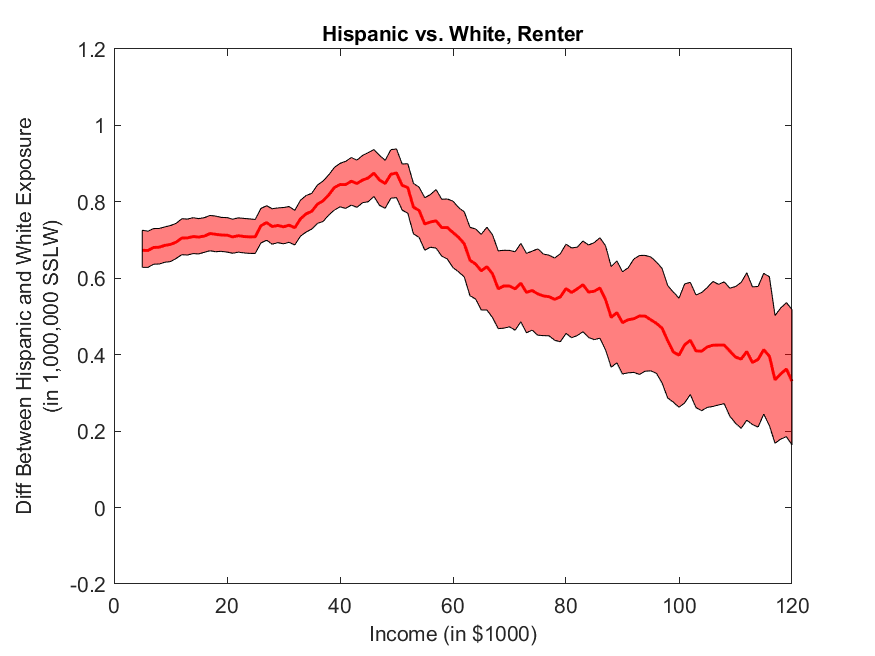


Figure (12d)

**S12 Fig. 5km Hogs Exposure Race Difference.**

We use hog farm data (provided by NC Department of Environmental Quality) and household demographic data (InfoUSA) to calculate the hog exposure (within 5km) difference (in 1,000,000 SSLW) between minority group and white for owners and renters. The solid line shows the exposure differences between Black/Hispanic and white residents. The shaded area shows the corresponding 95% confidence interval.

## **Poultry exposure differences.**

This section plots (see Figs S13-S15) the poultry (in 100,000 bird counts) exposure difference between minority group and white for owners and renters. The solid line shows the exposure differences between Black/Hispanic and white residents and the shaded area shows the corresponding confidence interval.


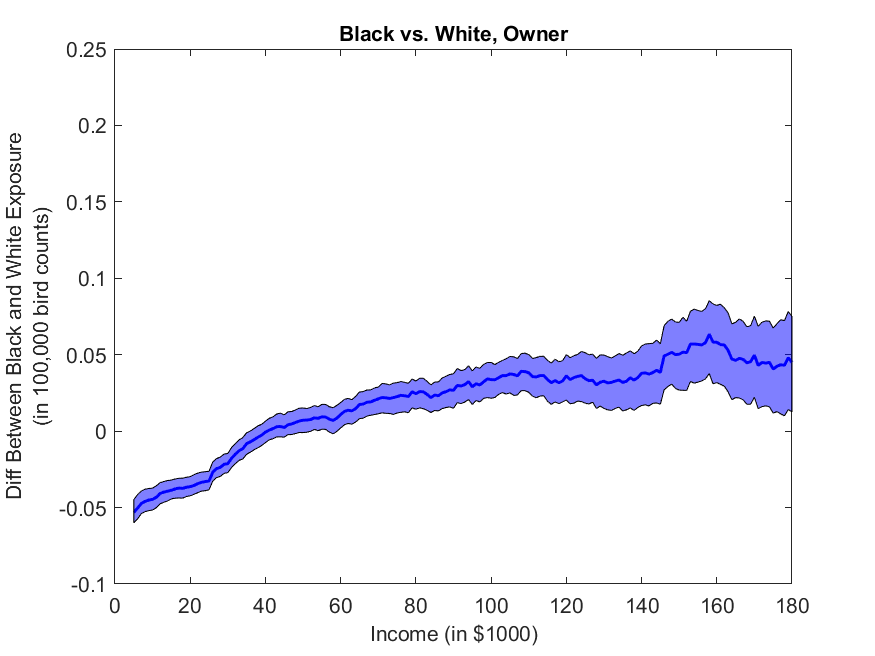


Figure (13a)


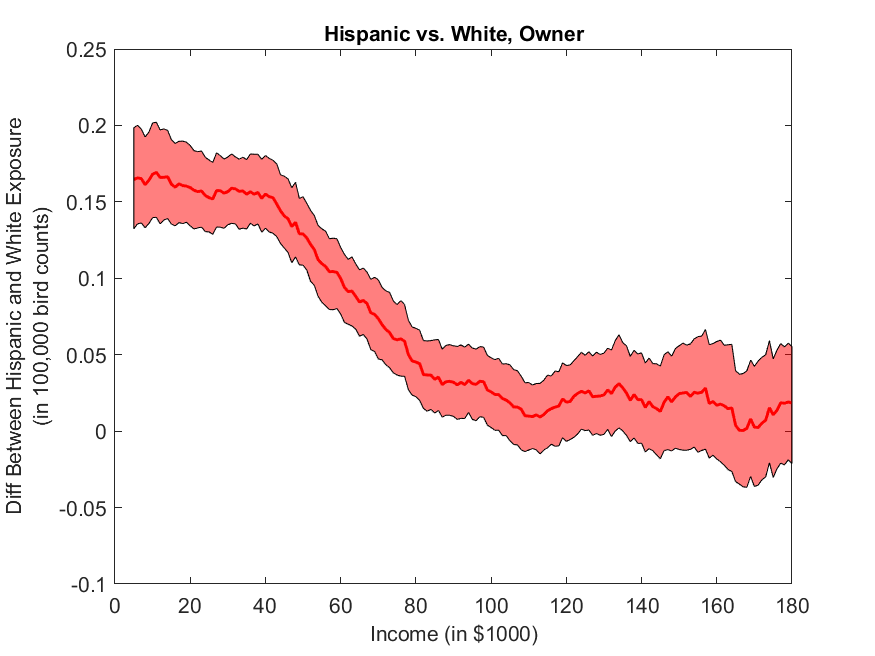


Figure (13b)


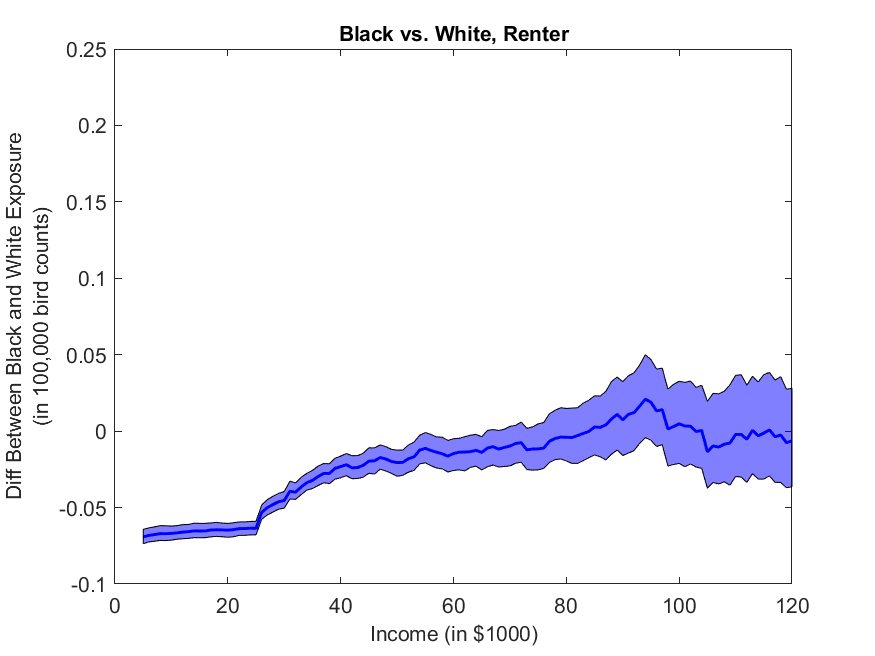


Figure (13c)


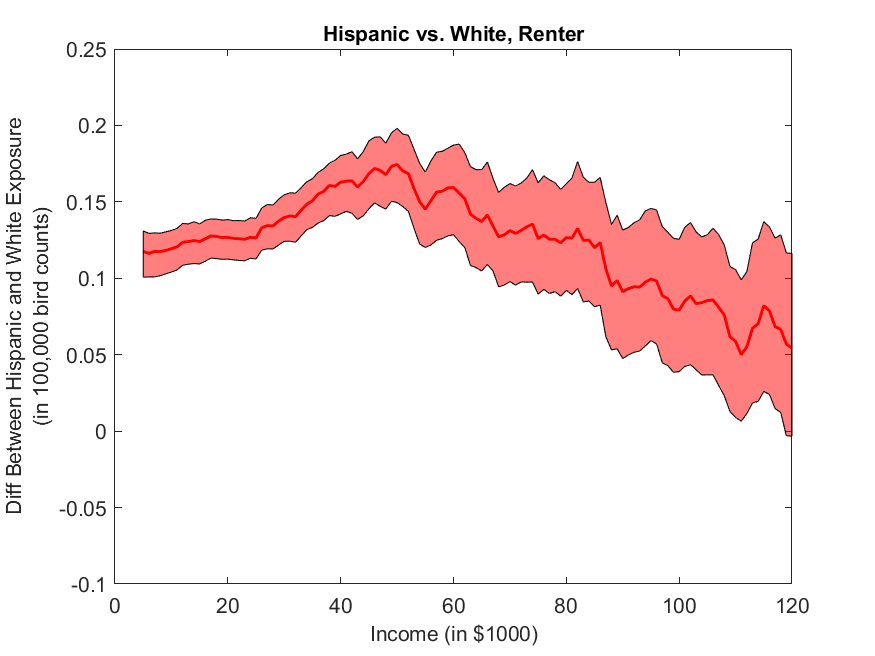


Figure (13d)

**S13 Fig. 3km Poultry Exposure Race Difference.**

We use poultry farm data (provided by Environmental Working Group) and household demographic data (InfoUSA) to calculate the hog exposure (within 3km) difference (in 100,000 bird counts) between minority group and white for owners and renters. The solid line shows the exposure differences between Black/Hispanic and white residents. The shaded area shows the corresponding 95% confidence interval.


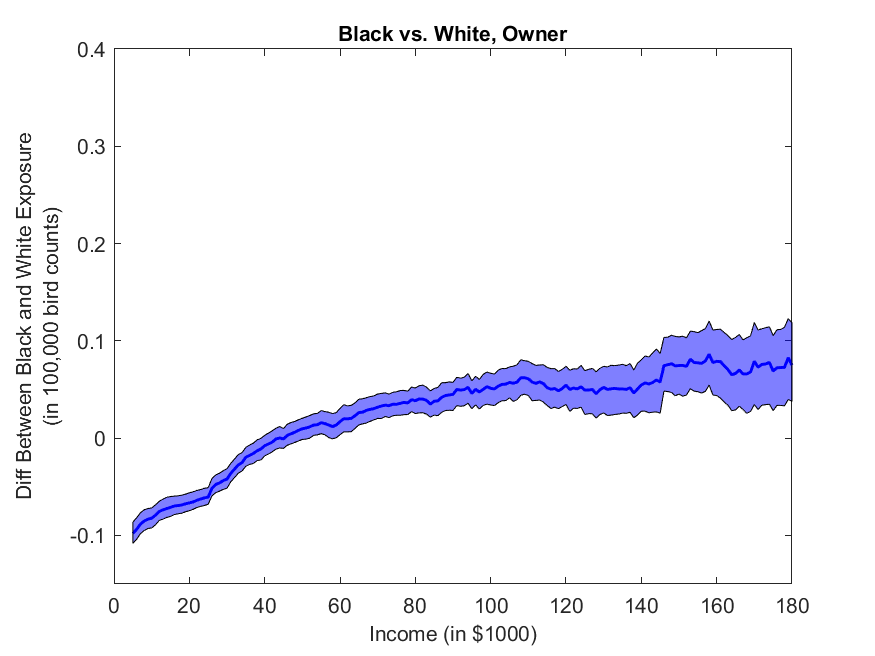


Figure (14a)


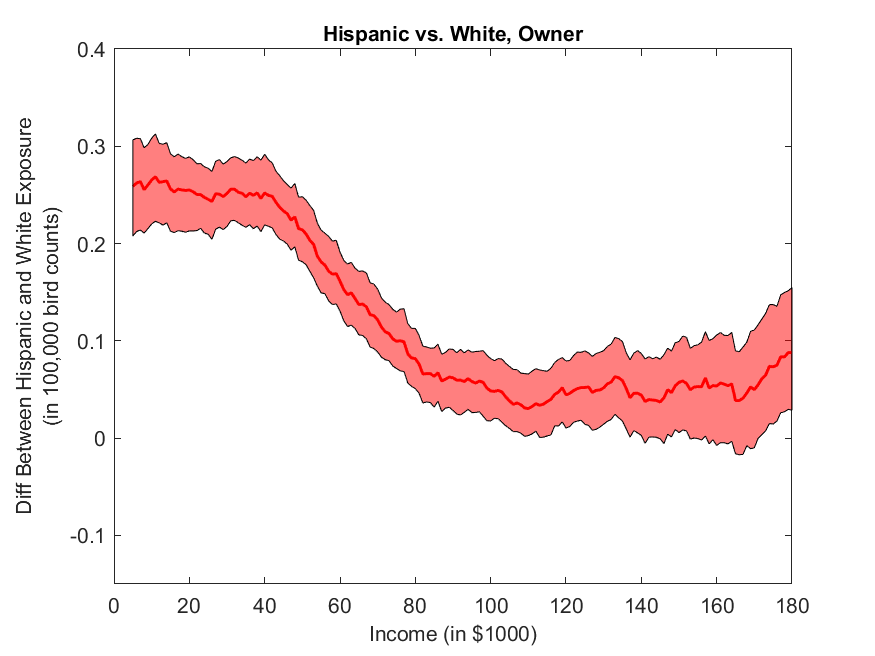


Figure (14b)


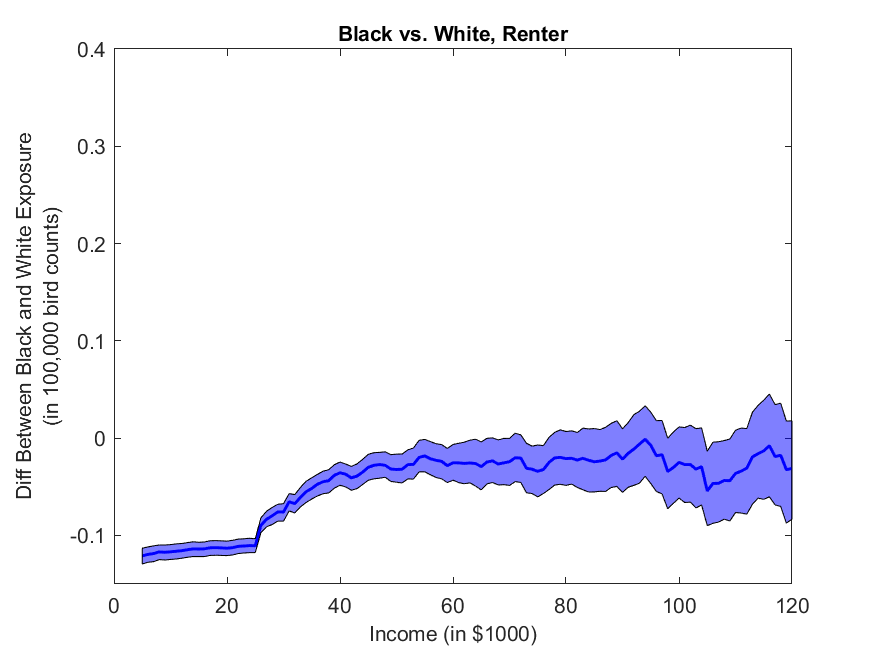


Figure (14c)


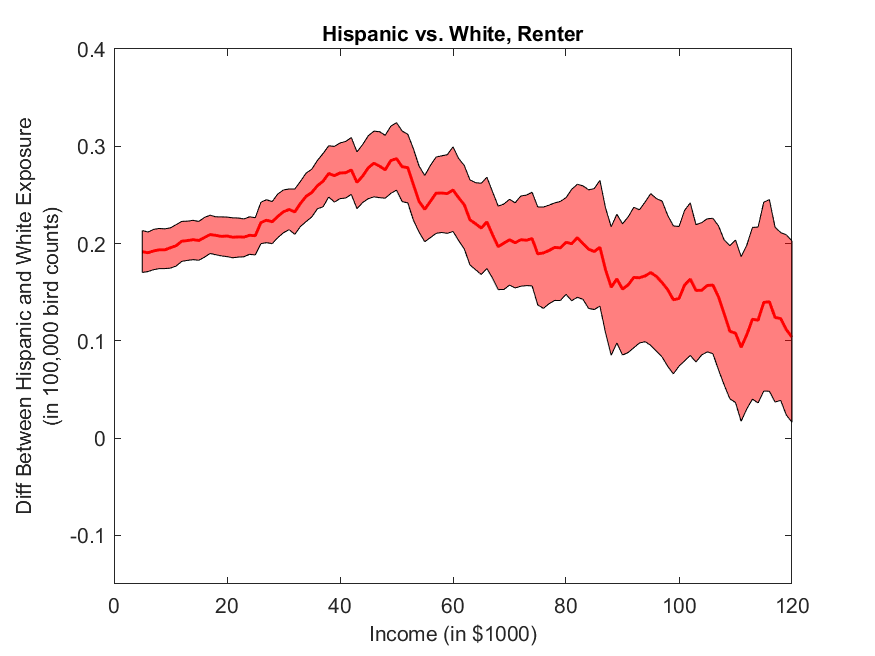


Figure (14d)

**S14 Fig. 4km Poultry Exposure Race Difference.**

We use poultry farm data (provided by Environmental Working Group) and household demographic data (InfoUSA) to calculate the hog exposure (within 4km) difference (in 100,000 bird counts) between minority group and white for owners and renters. The solid line shows the exposure differences between Black/Hispanic and white residents. The shaded area shows the corresponding 95% confidence interval.


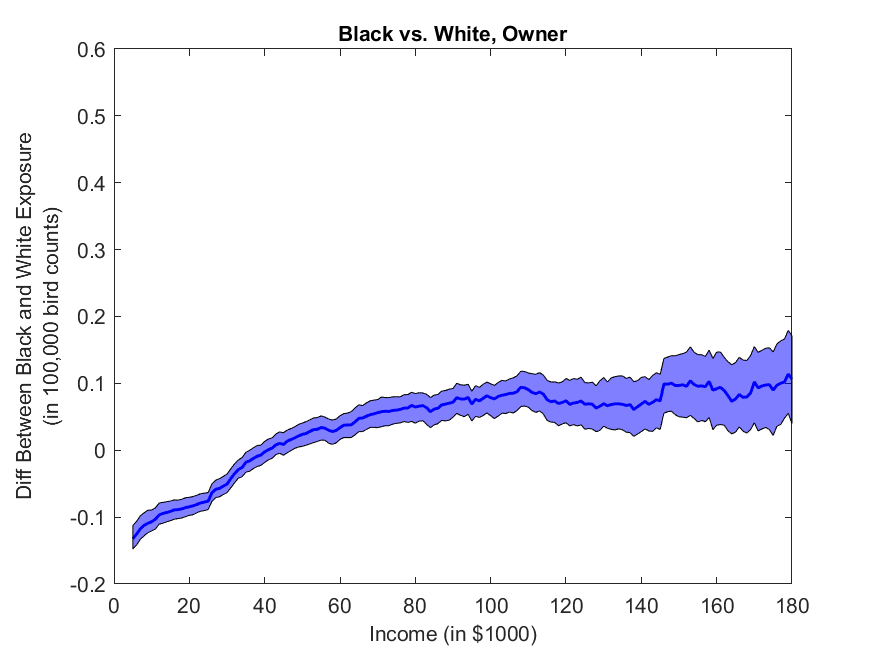


Figure (15a)


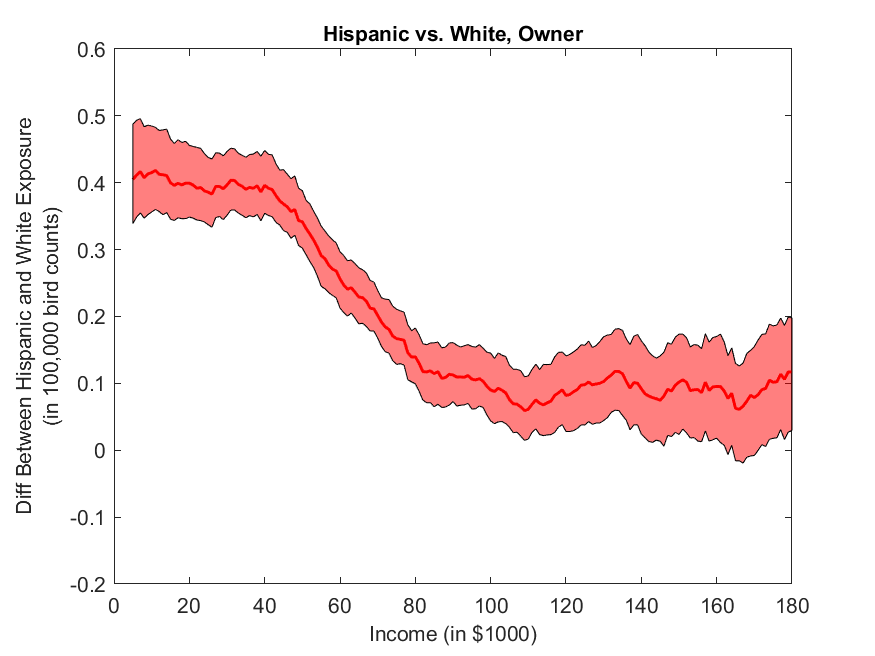


Figure (15b)


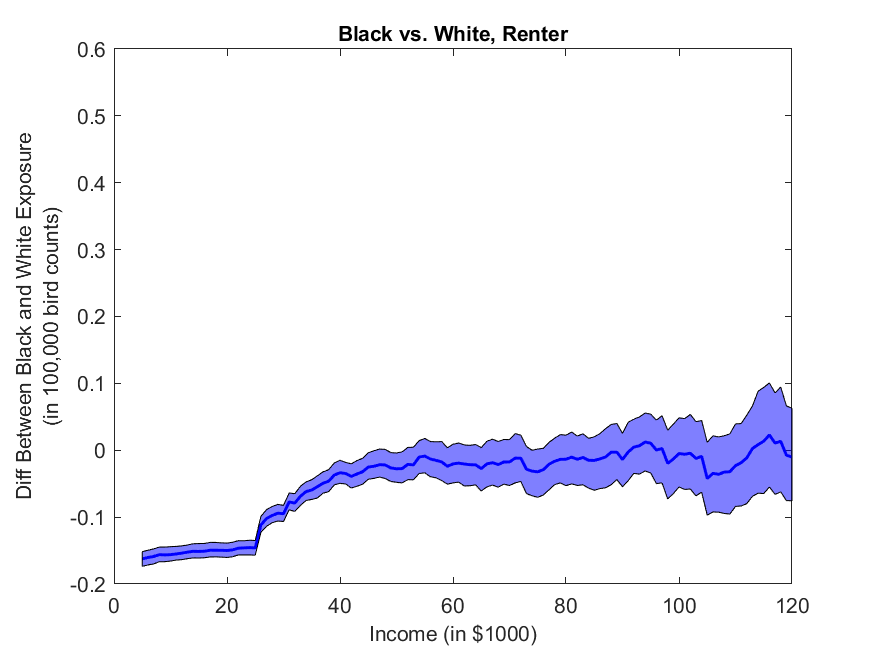


Figure (15c)


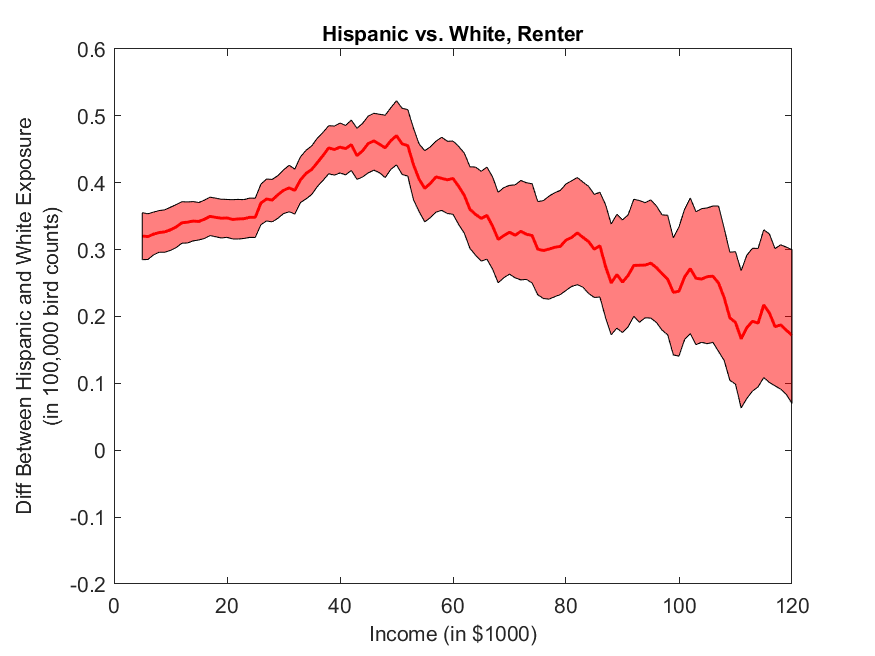


Figure (15d)

**S15 Fig. 5km Poultry Exposure Race Difference.**

We use poultry farm data (provided by Environmental Working Group) and household demographic data (InfoUSA) to calculate the hog exposure (within 5km) difference (in 100,000 bird counts) between minority group and white for owners and renters. The solid line shows the exposure differences between Black/Hispanic and white residents. The shaded area shows the corresponding 95% confidence interval.

## **CAFO exposure differences by water.**

This section plots (see Figs S16-S18) the hog (in 1,000,000 SSLW) and poultry (in 100,000 bird counts) exposure difference between minority group and white for private well and community water system dependent households. The lines show the exposure differences between Black/Hispanic and white residents, where the solid and dashed lines show the exposure differences for groundwater- and piped-water-dependent households. The shaded area shows the corresponding 95% confidence interval.


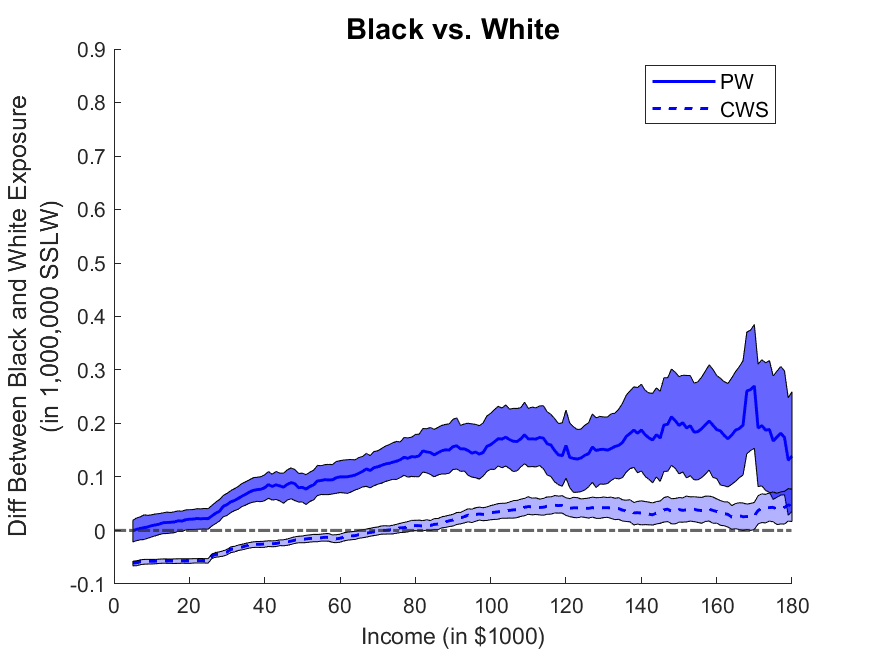


Figure (16a)


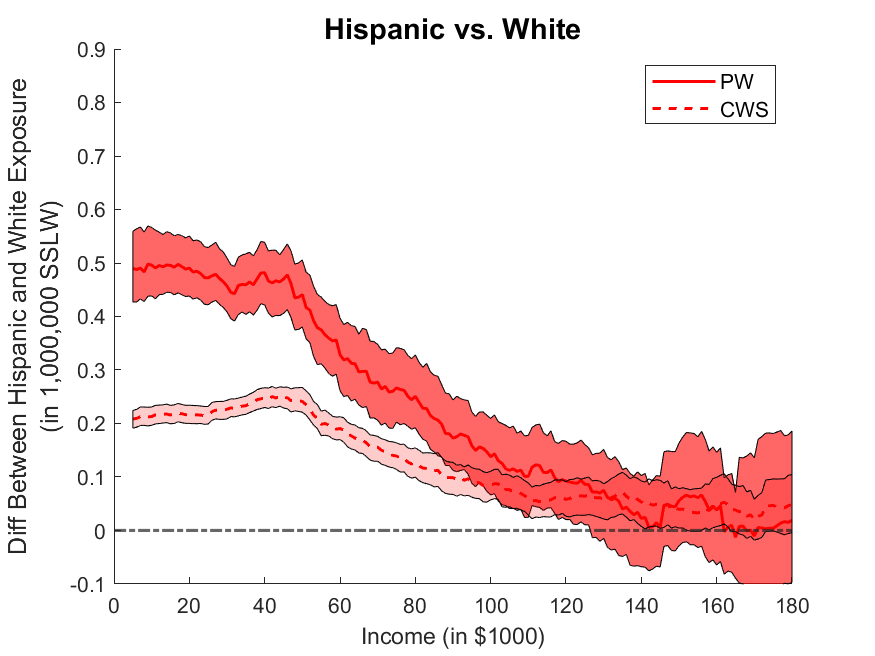


Figure (16b)


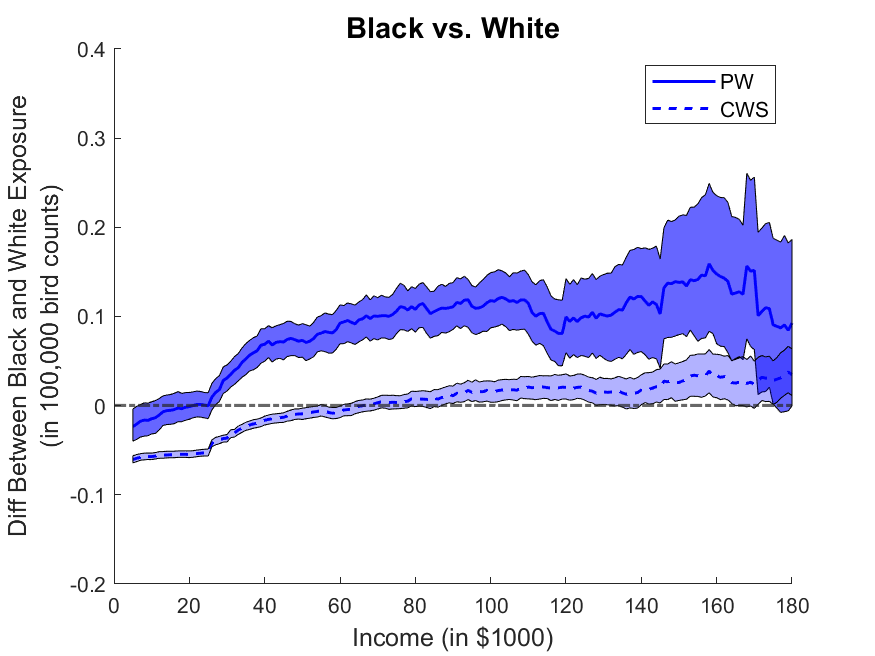


Figure (16c)


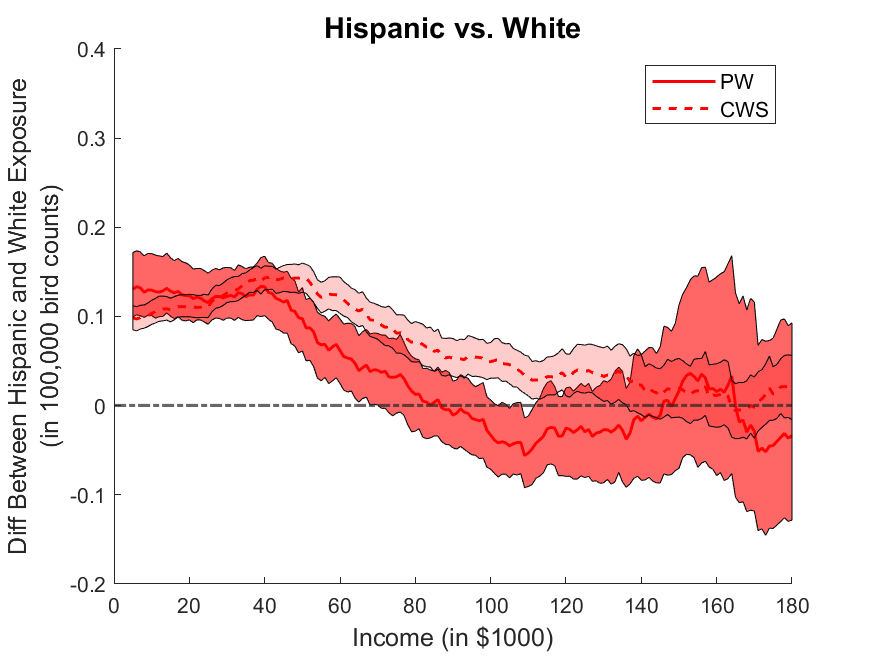


Figure (16d)

**S16 Fig. 3km CAFO Exposure Race Difference, Private Well vs Community Water System.**

We use hog farm data (provided by NC Department of Environmental Quality), poultry farm data (provided by Environmental Working Group), and household demographic data (InfoUSA) to calculate the hog (in 1,000,000 SSLW) and poultry (in 100,000 bird counts) exposure (within 3km) difference between minority group and white for private well and community water system dependent households. The lines show the exposure differences between Black/Hispanic and white residents, where the solid and dashed lines show the exposure differences for private well and community water system dependent households. The shaded area shows the corresponding 95% confidence interval.


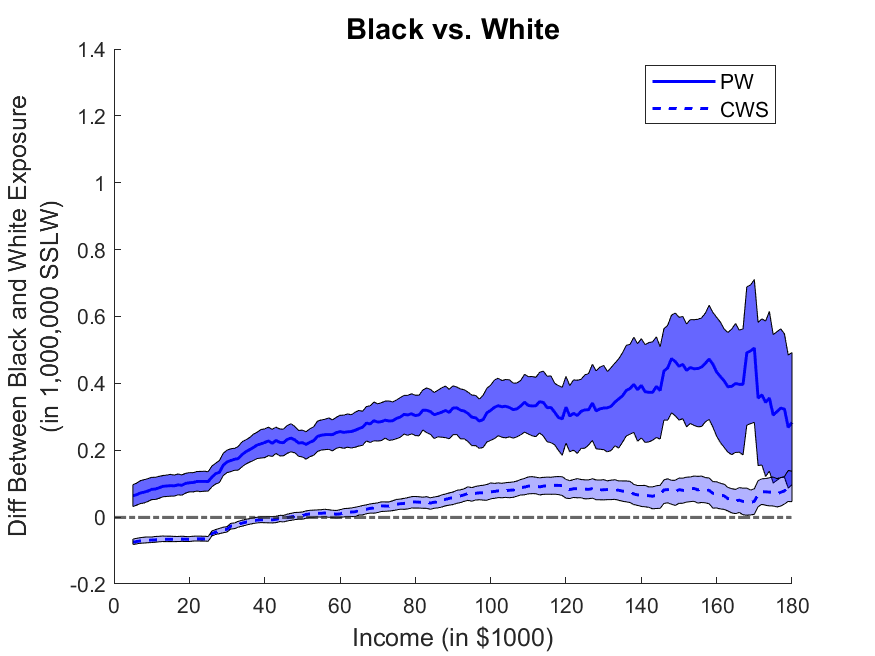


Figure (17a)


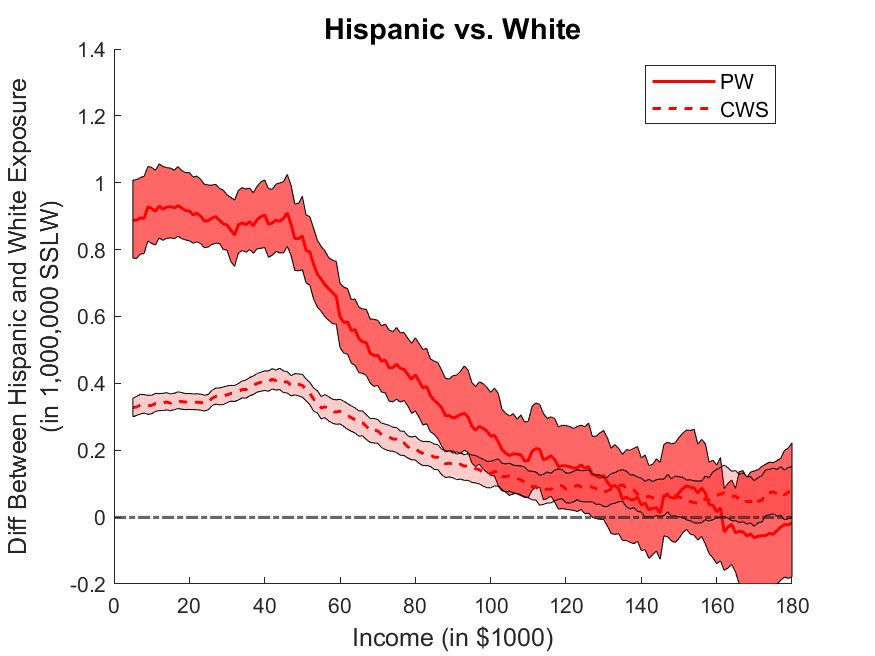


Figure (17b)


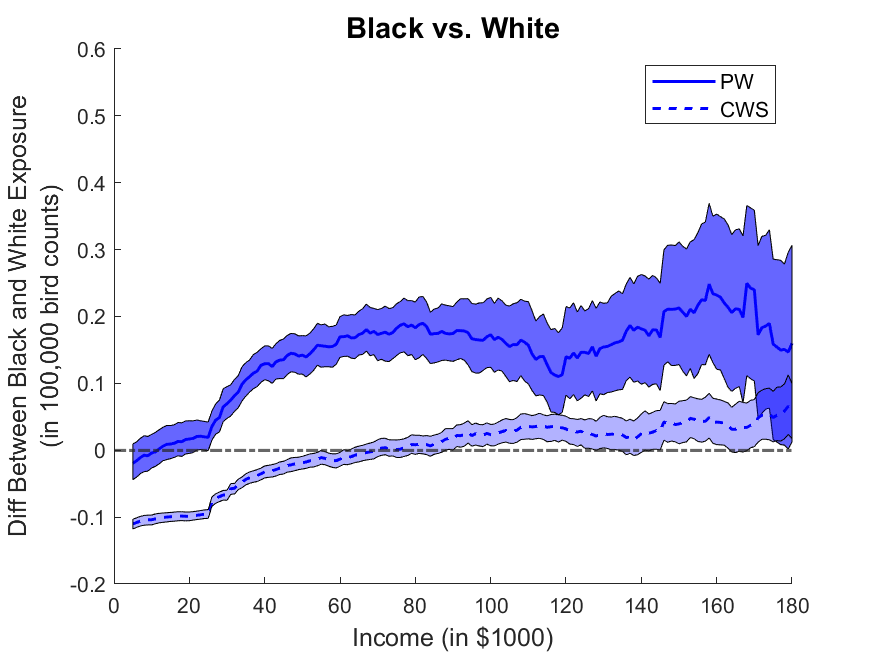


Figure (17c)


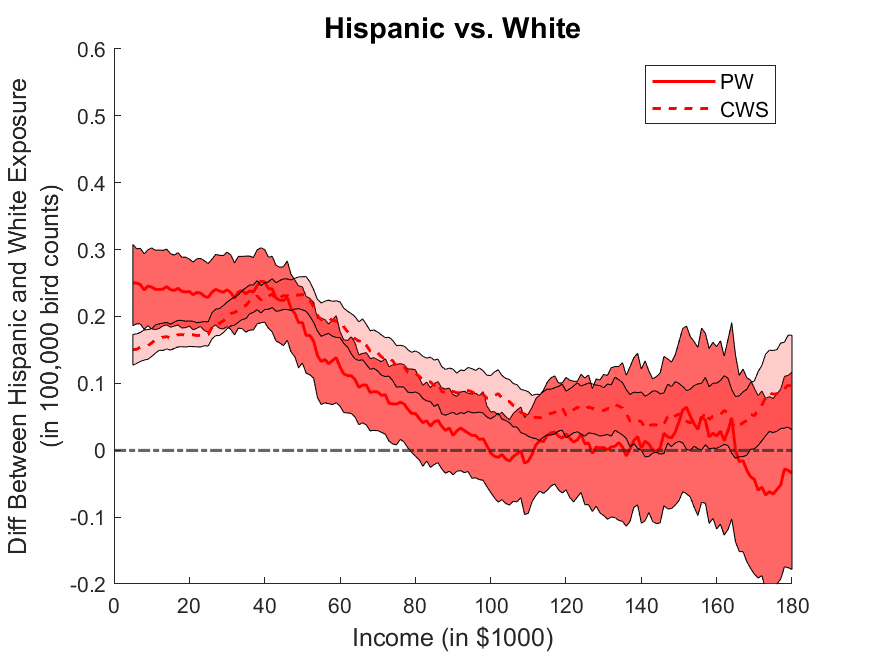


Figure (17d)

**S17 Fig. 4km CAFO Exposure Race Difference, Private Well vs Community Water System.**

We use hog farm data (provided by NC Department of Environmental Quality), poultry farm data (provided by Environmental Working Group), and household demographic data (InfoUSA) to calculate the hog (in 1,000,000 SSLW) and poultry (in 100,000 bird counts) exposure (within 4km) difference between minority group and white for private well and community water system dependent households. The lines show the exposure differences between Black/Hispanic and white residents, where the solid and dashed lines show the exposure differences for private well and community water system dependent households. The shaded area shows the corresponding 95% confidence interval.


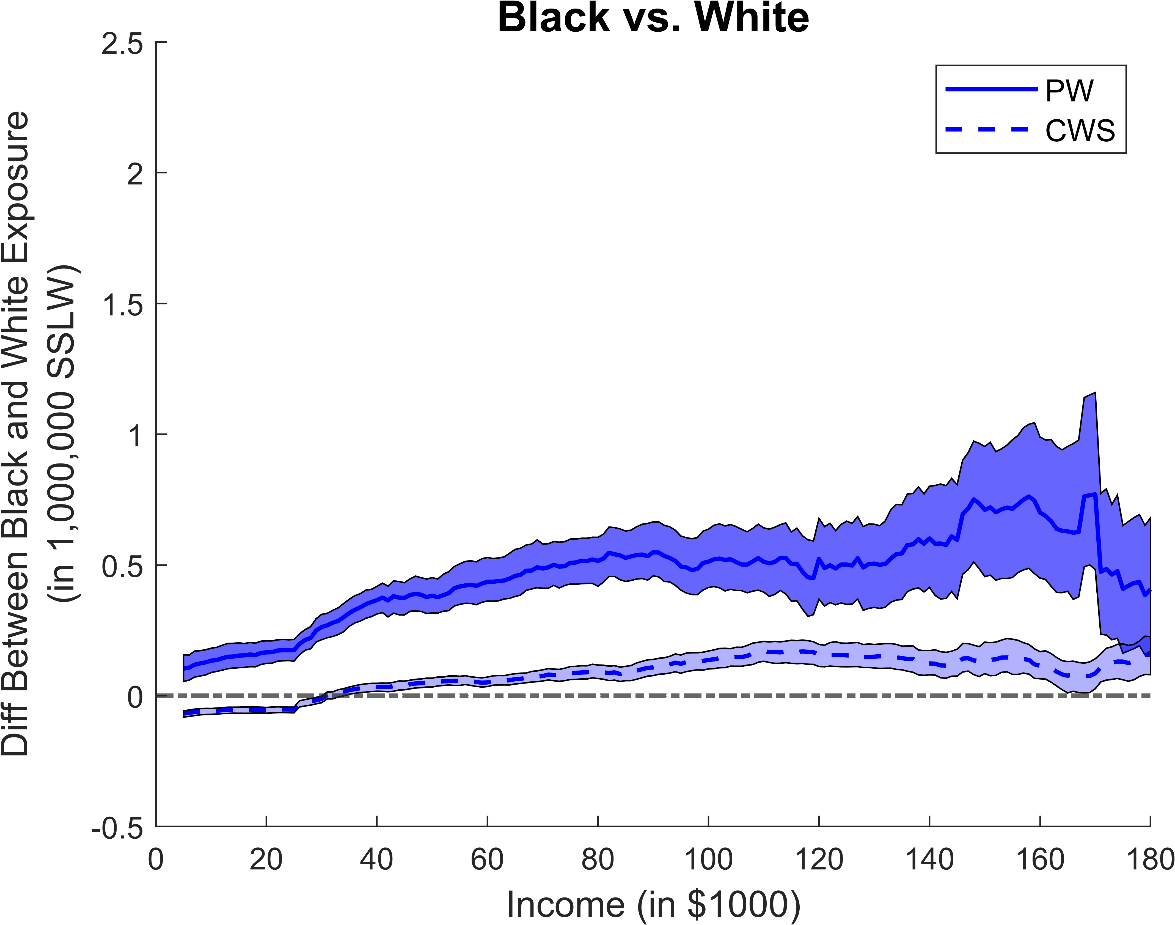


Figure (18a)


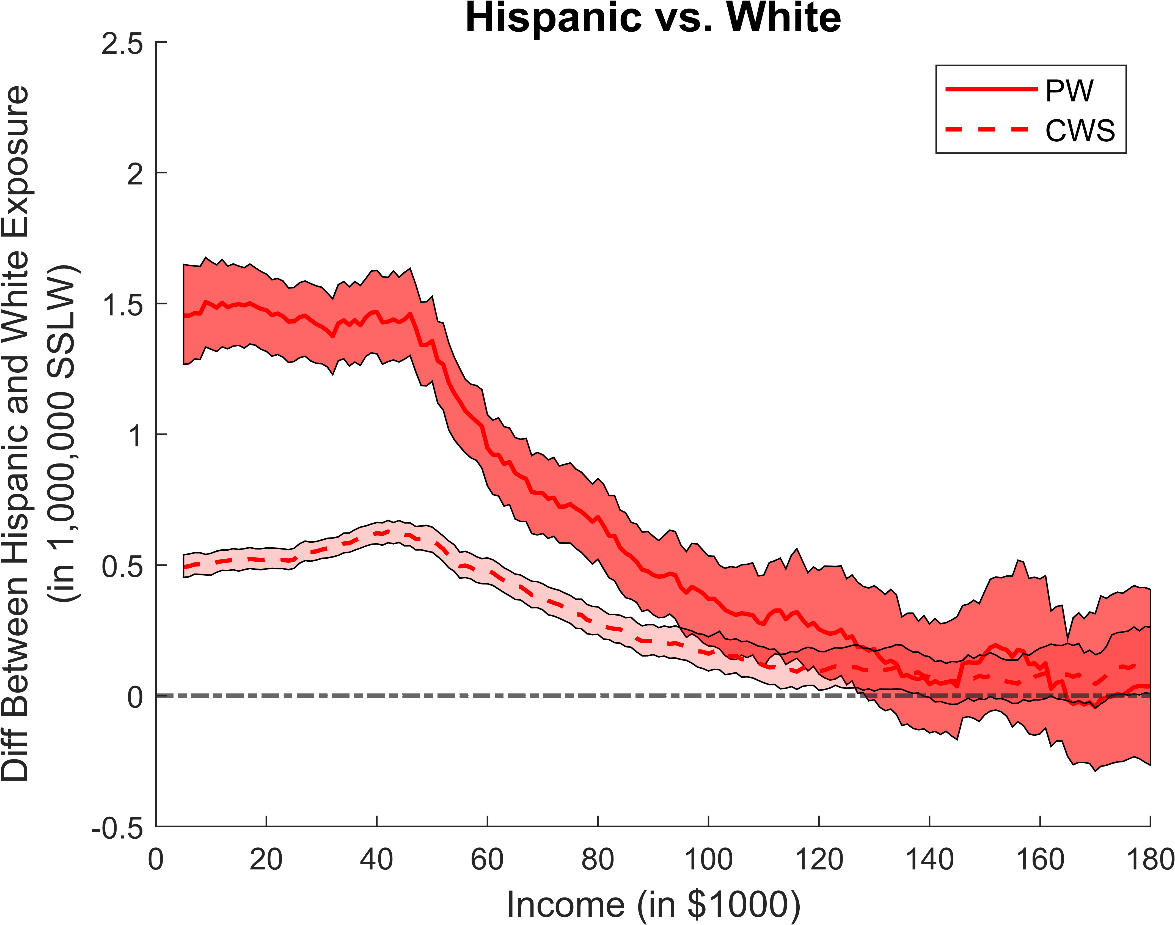


Figure (18b)


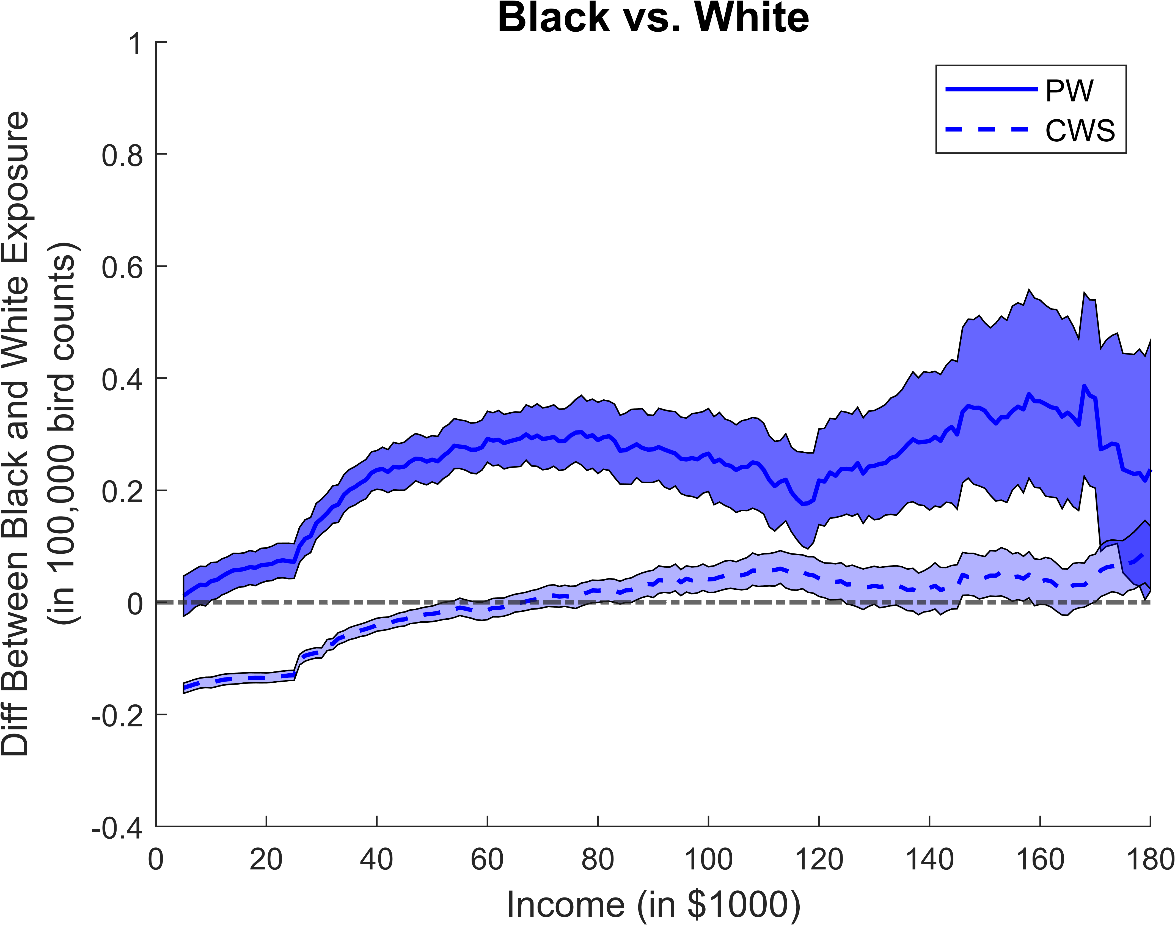


Figure (18c)


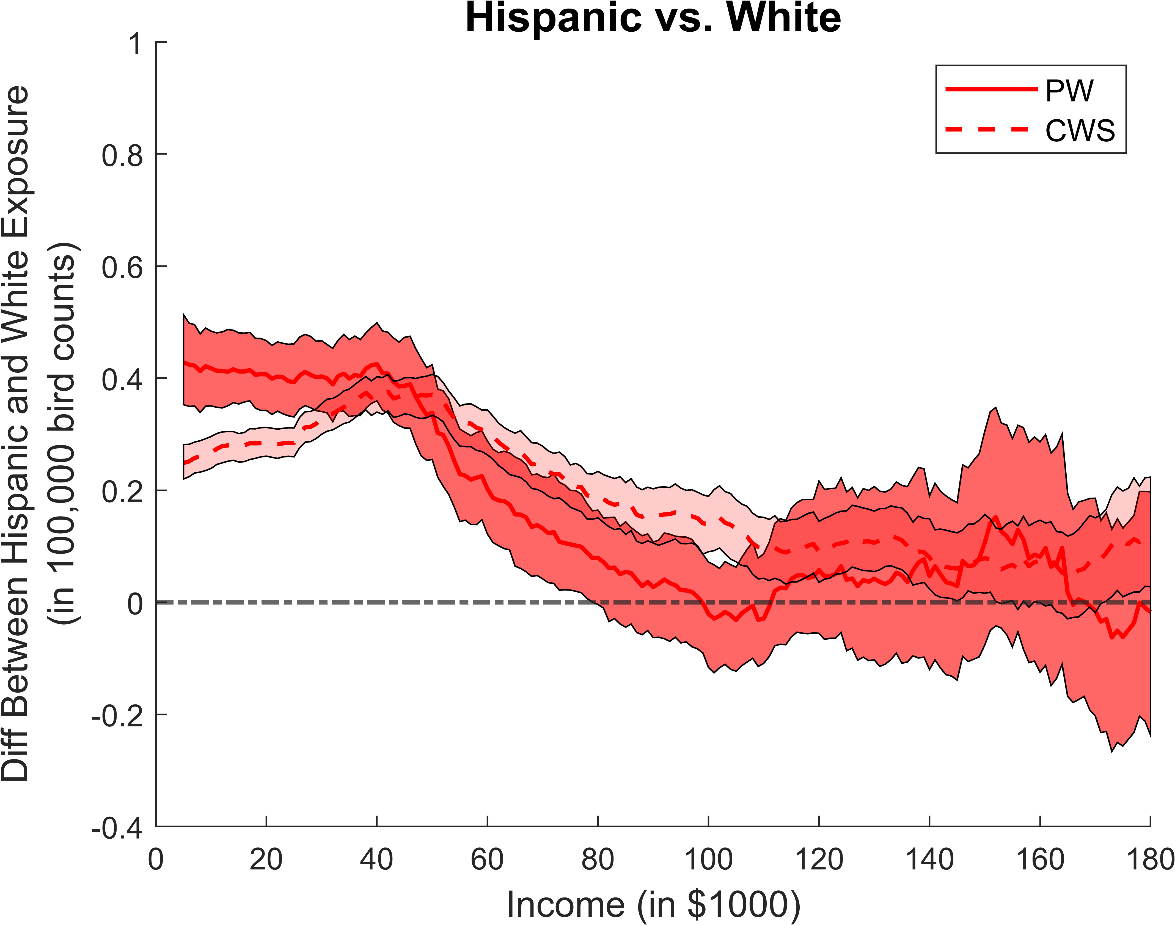


Figure (18d)

**S18 Fig. 5km CAFO Exposure Race Difference, Private Well vs Community Water System.**

We use hog farm data (provided by NC Department of Environmental Quality), poultry farm data (provided by Environmental Working Group), and household demographic data (InfoUSA) to calculate the hog (in 1,000,000 SSLW) and poultry (in 100,000 bird counts) exposure (within 5km) difference between minority group and white for private well and community water system dependent households. The lines show the exposure differences between Black/Hispanic and white residents, where the solid and dashed lines show the exposure differences for private well and community water system dependent households. The shaded area shows the corresponding 95% confidence interval.

## **Hog exposure differences by water, and by owners and renters.**

This section plots (see Figs S19-S21) the hog (in 1,000,000 SSLW) exposure difference between minority group and white for private well and community water system dependent owners and renters. The lines show the exposure differences between Black/Hispanic and white residents, where the solid and dashed lines show the exposure differences for groundwater- and piped-water-dependent households. The shaded area shows the corresponding 95% confidence interval.


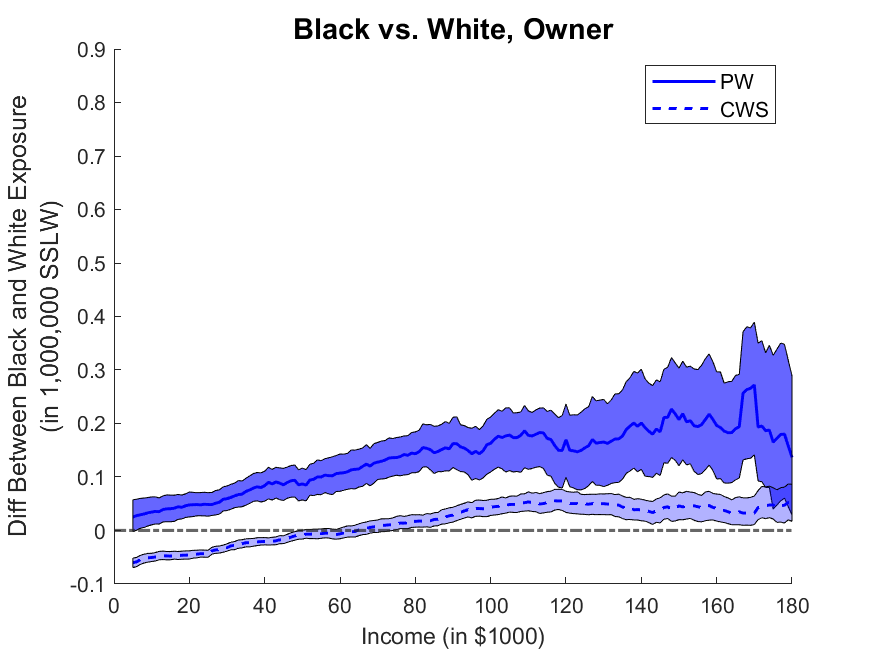


Figure (19a)


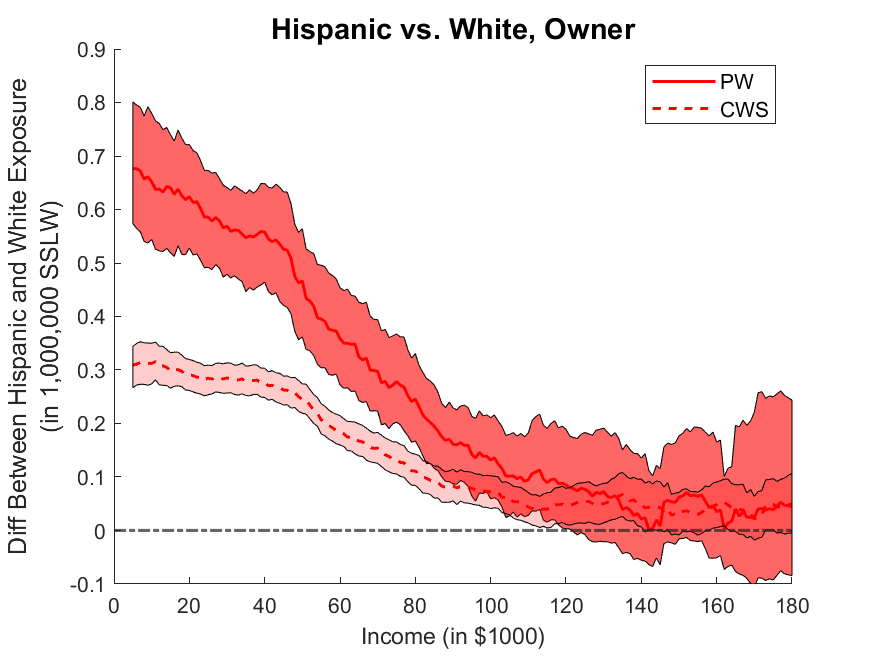


Figure (19b)


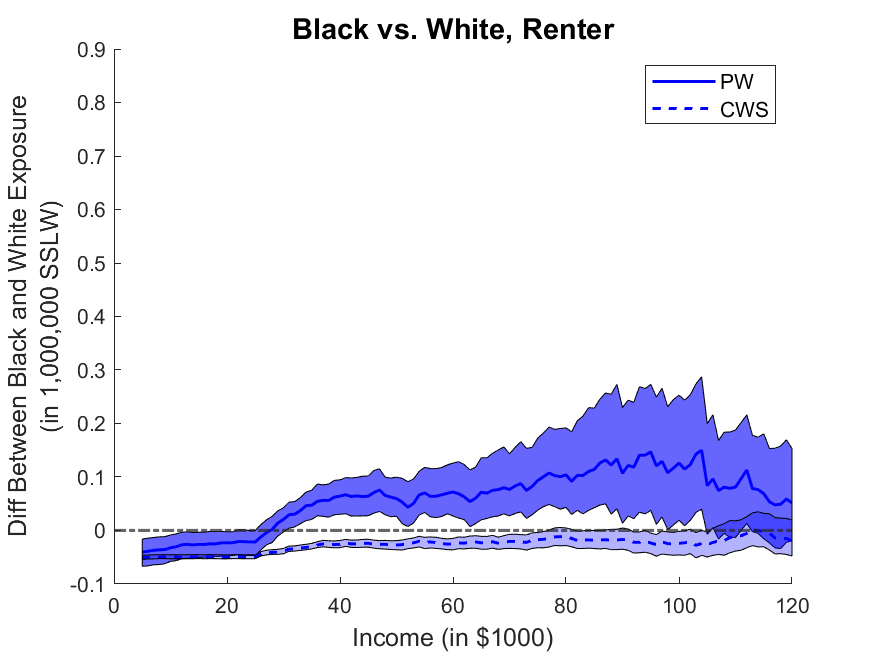


Figure (19c)


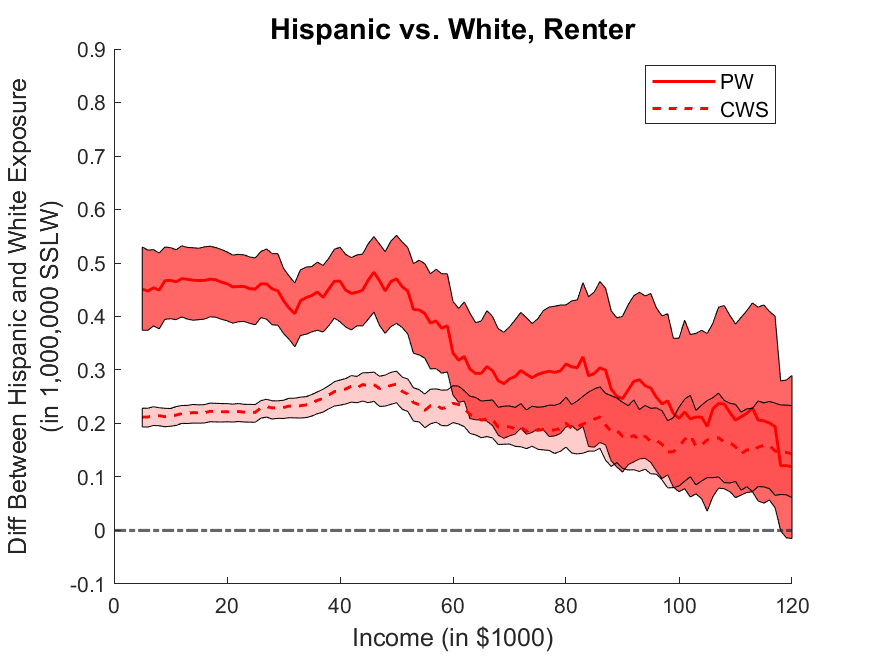


Figure (19d)

**S19 Fig. 3km Hogs Exposure Race Difference by Owner/Renter, Private Well vs Community Water System.**

We use hog farm data (provided by NC Department of Environmental Quality) and household demographic data (InfoUSA) to calculate the hog exposure (within 3km) difference (in 1,000,000 SSLW) between minority group and white for private well and community water system dependent owners and renters. The lines show the exposure differences between Black/Hispanic and white residents, where the solid and dashed lines show the exposure differences for private well and community water system dependent households. The shaded area shows the corresponding 95% confidence interval.


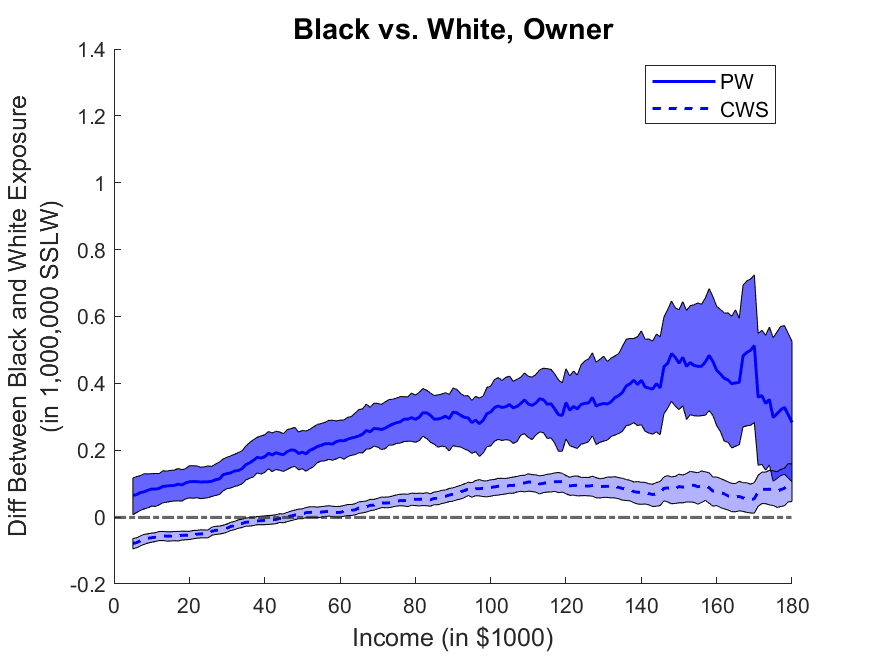


Figure (20a)


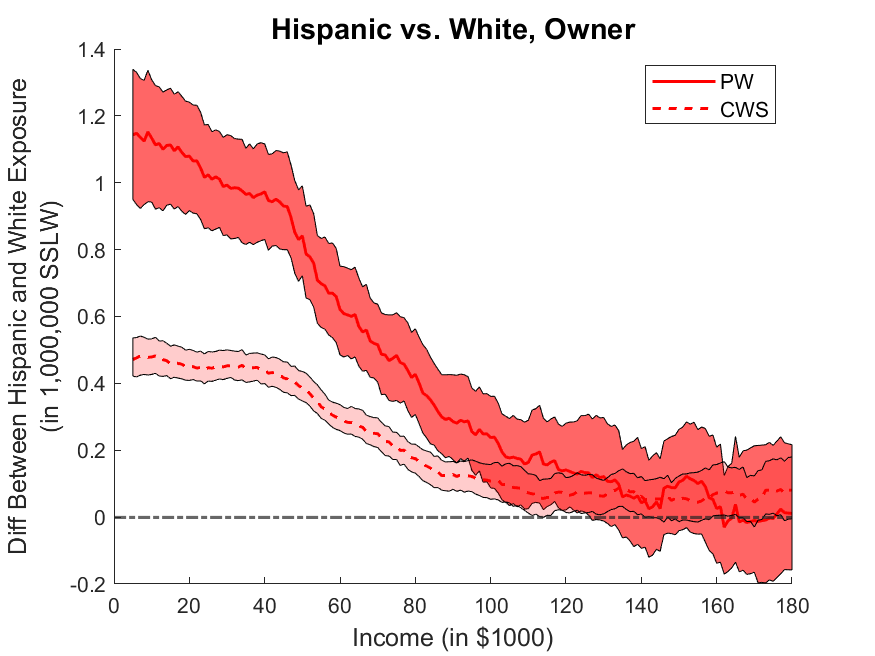


Figure (20b)


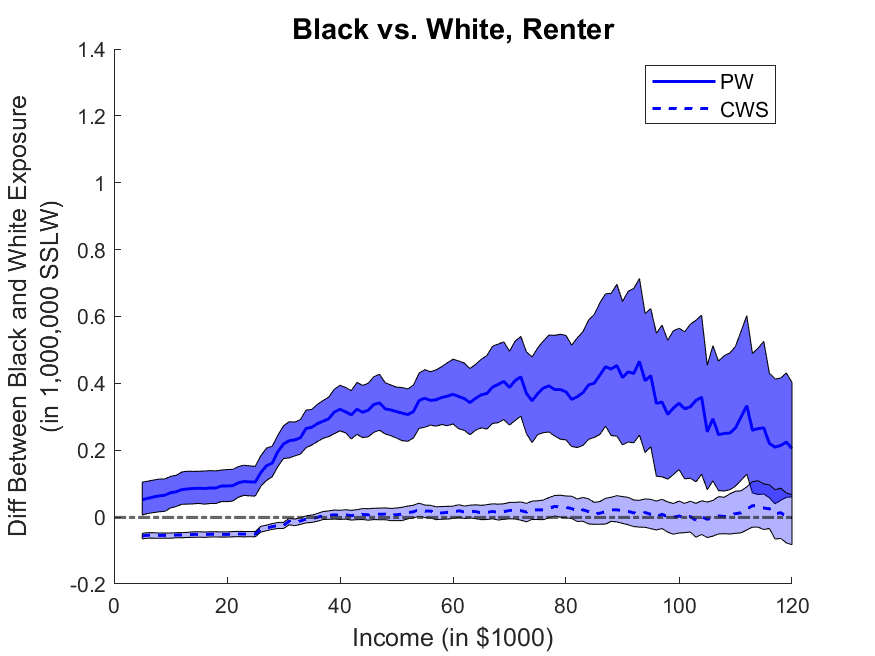


Figure (20c)


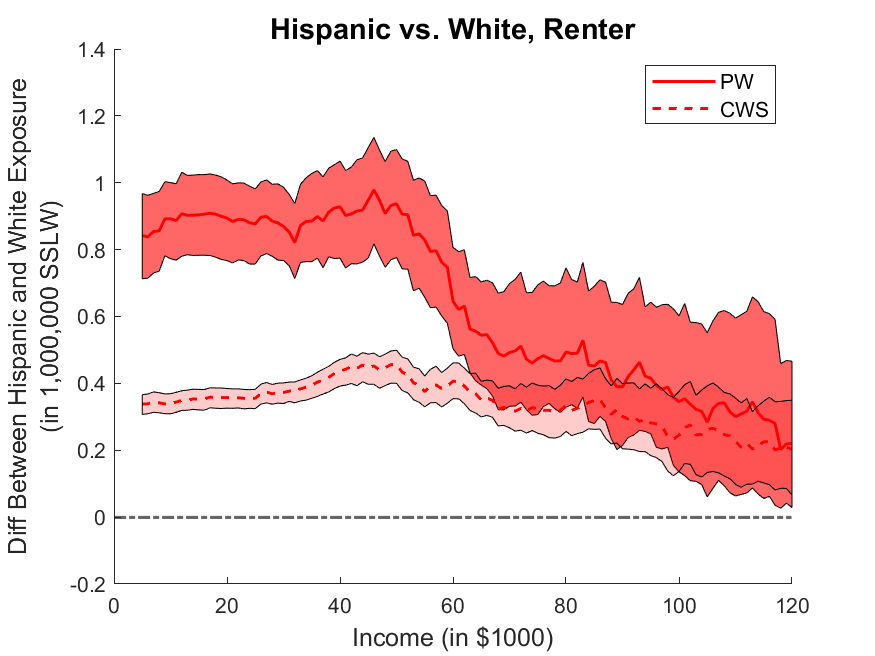


Figure (20d)

**S20 Fig. 4km Hogs Exposure Race Difference by Owner/Renter, Private Well vs Community Water System.**

We use hog farm data (provided by NC Department of Environmental Quality) and household demographic data (InfoUSA) to calculate the hog exposure (within 4km) difference (in 1,000,000 SSLW) between minority group and white for private well and community water system dependent owners and renters. The lines show the exposure differences between Black/Hispanic and white residents, where the solid and dashed lines show the exposure differences for private well and community water system dependent households. The shaded area shows the corresponding 95% confidence interval.


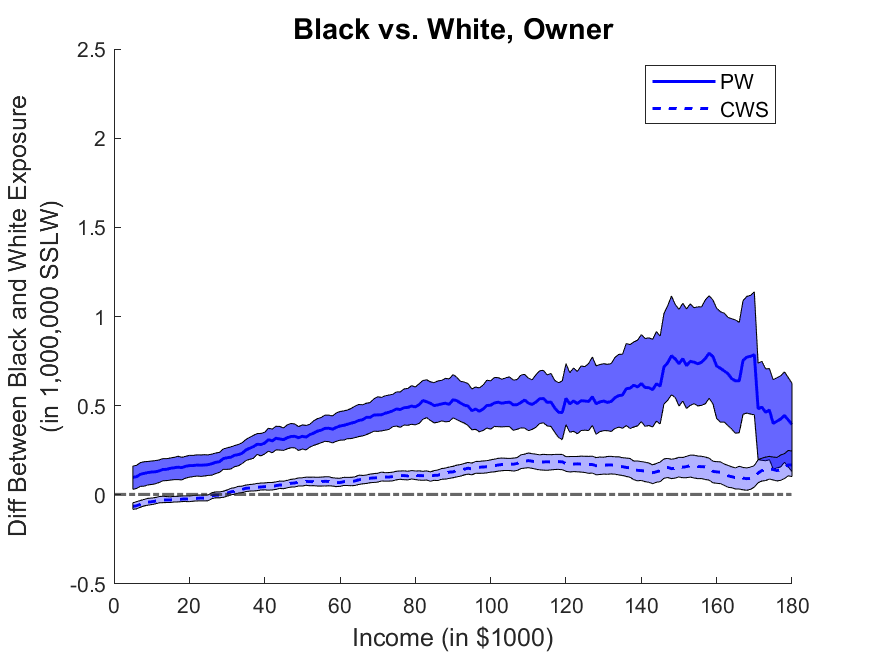


Figure (21a)


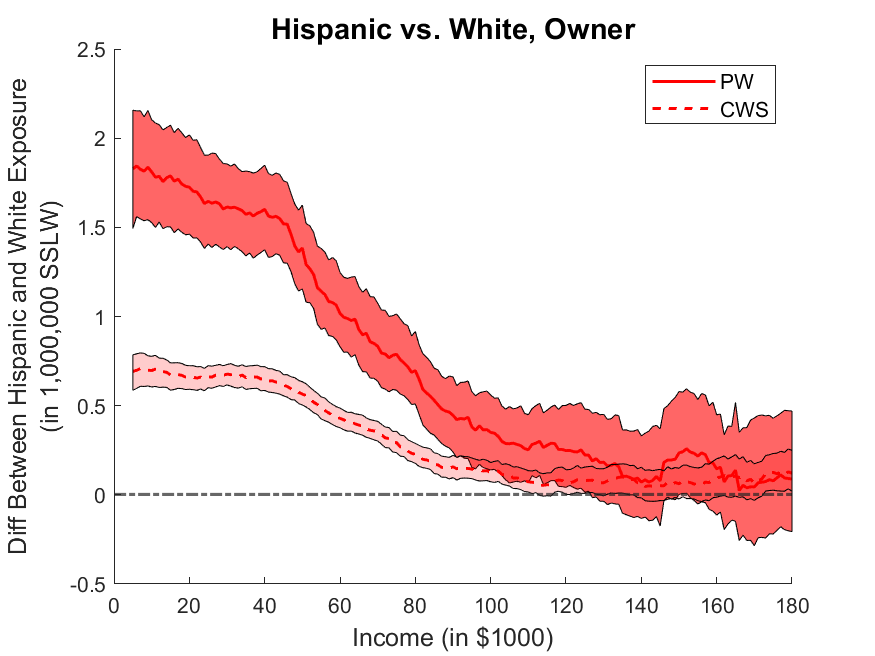


Figure (21b)


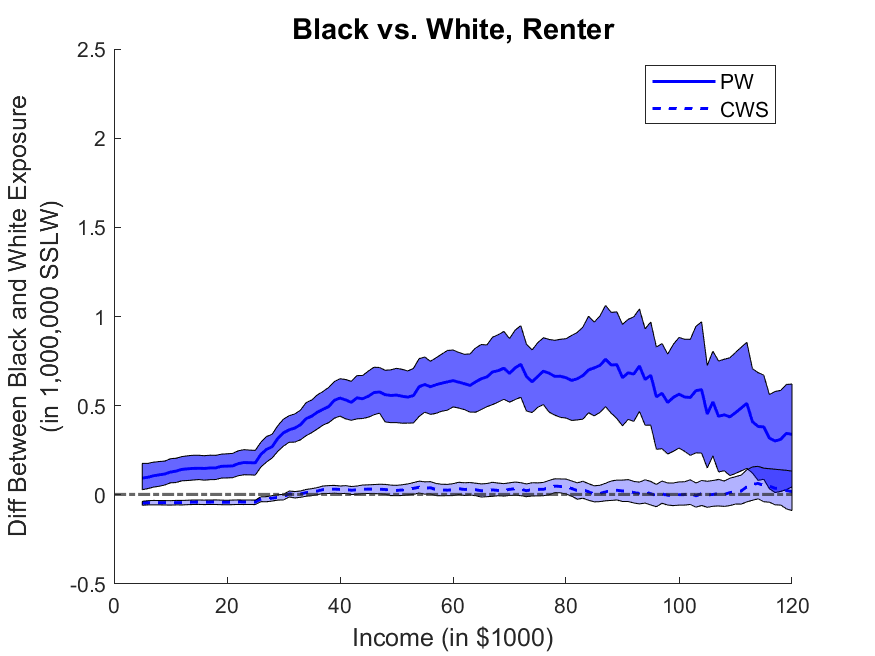


Figure (21c)


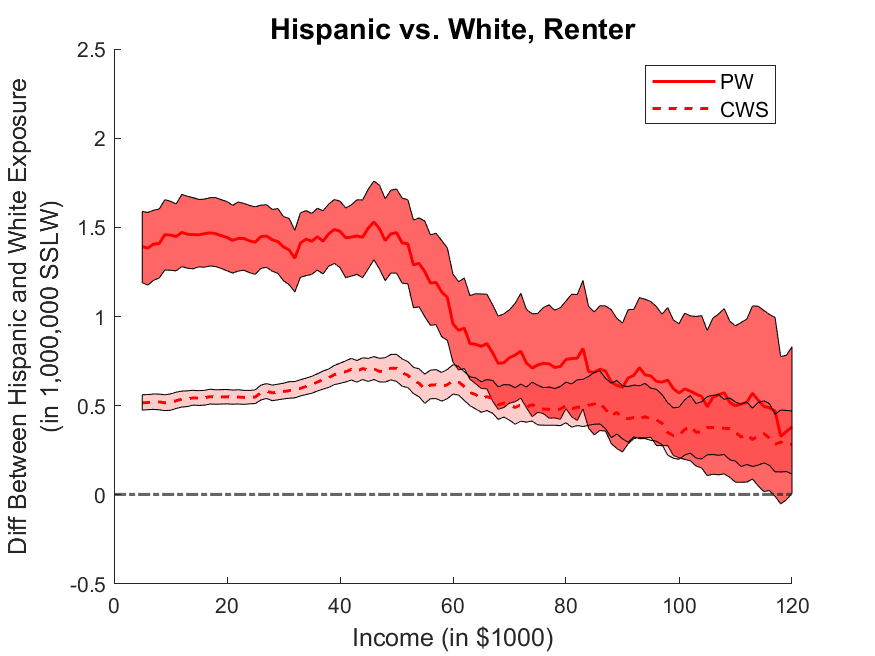


Figure (21d)

**S21 Fig. 5km Hogs Exposure Race Difference by Owner/Renter, Private Well vs Community Water System.**

We use hog farm data (provided by NC Department of Environmental Quality) and household demographic data (InfoUSA) to calculate the hog exposure (within 5km) difference (in 1,000,000 SSLW) between minority group and white for private well and community water system dependent owners and renters. The lines show the exposure differences between Black/Hispanic and white residents, where the solid and dashed lines show the exposure differences for private well and community water system dependent households. The shaded area shows the corresponding 95% confidence interval.

## **Poultry exposure differences by water, and by owners and renters.**

This section plots (see Figs S22-S24) the poultry (in 100,000 bird counts) exposure difference between minority group and white for groundwater- and piped-water-dependent owners and renters. The lines show the exposure differences between Black/Hispanic and white residents, where the solid and dashed lines show the exposure differences for private well and community water system dependent households. The shaded area shows the corresponding 95% confidence interval.


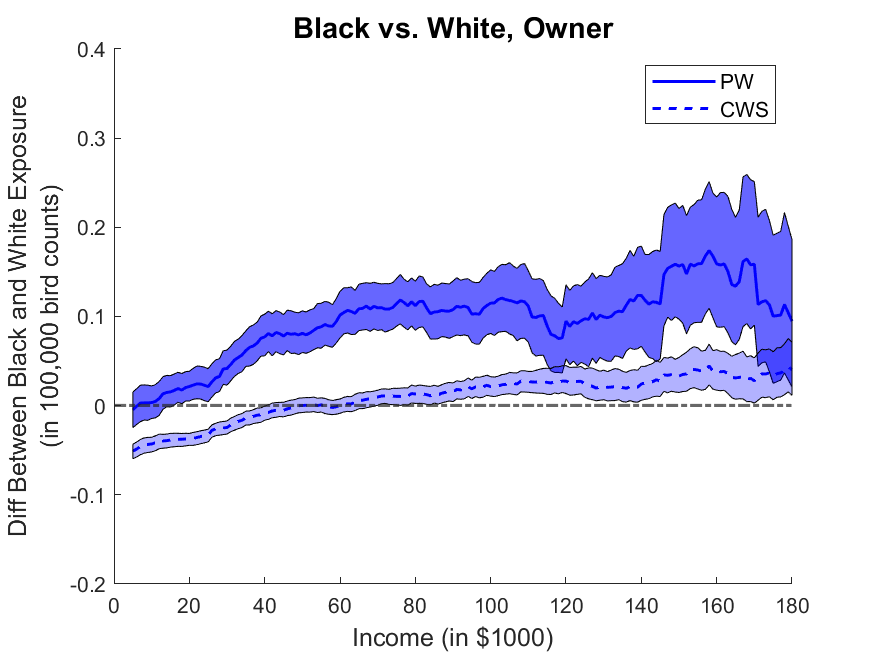


Figure (22a)


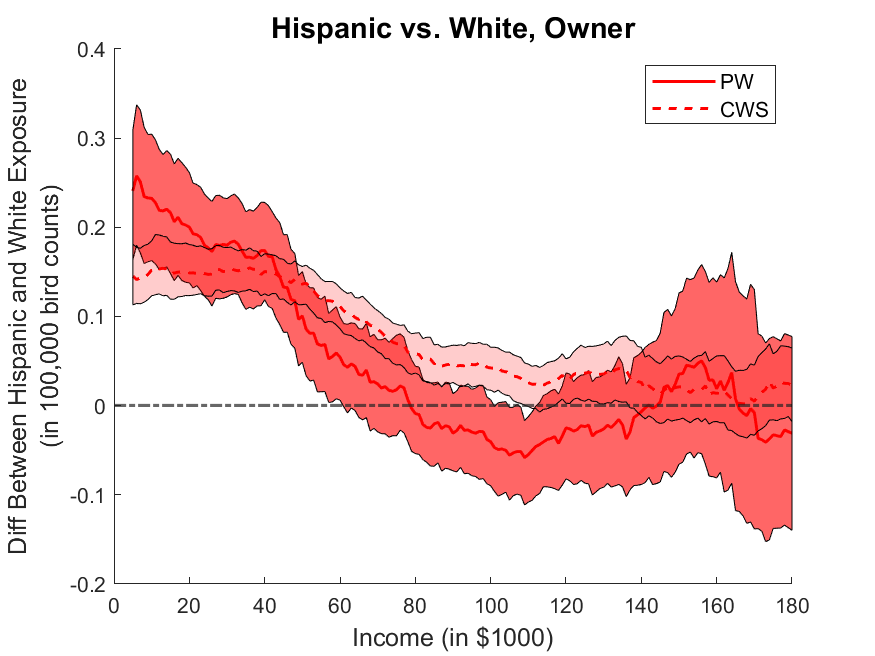


Figure (22b)


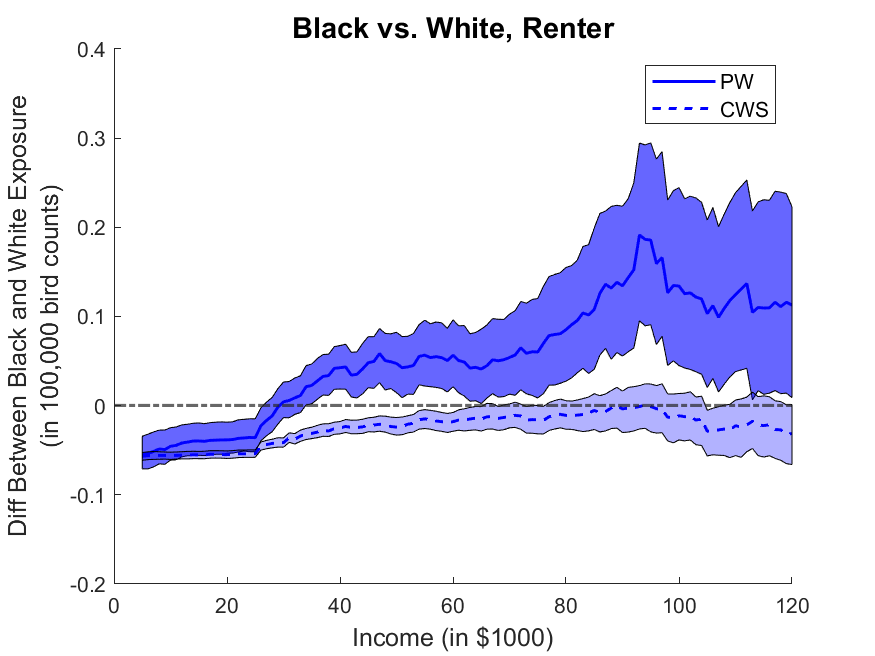


Figure (22c)


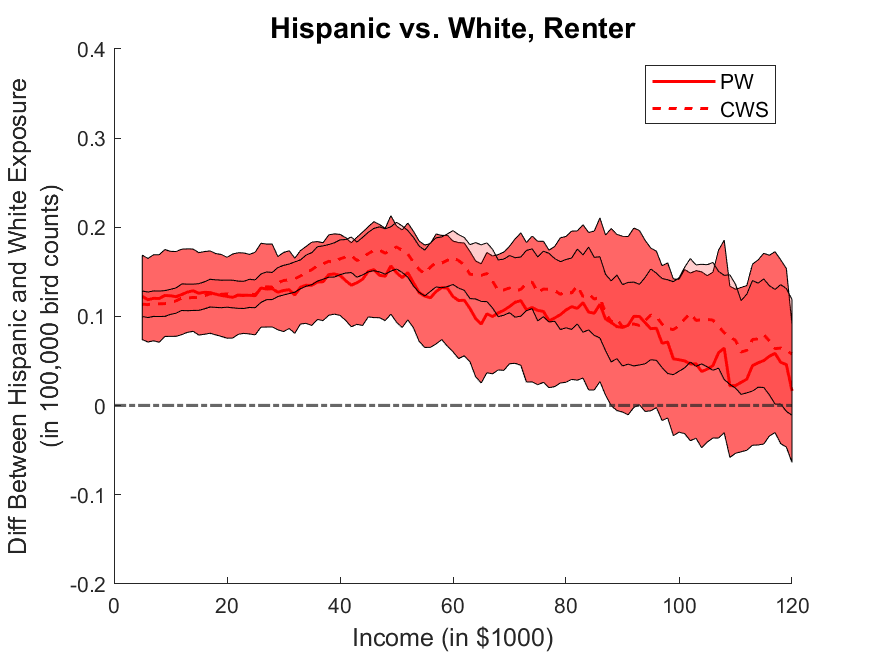


Figure (22d)

**S22 Fig. 3km Poultry Exposure Race Difference by Owner/Renter, Private Well vs Community Water System.**

We use poultry farm data (provided by Environmental Working Group) and household demographic data (InfoUSA) to calculate the poultry exposure (within 3km) difference (in 100,000 bird counts) between minority group and white for private well and community water system dependent owners and renters. The lines show the exposure differences between Black/Hispanic and white residents, where the solid and dashed lines show the exposure differences for private well and community water system dependent households. The shaded area shows the corresponding 95% confidence interval.


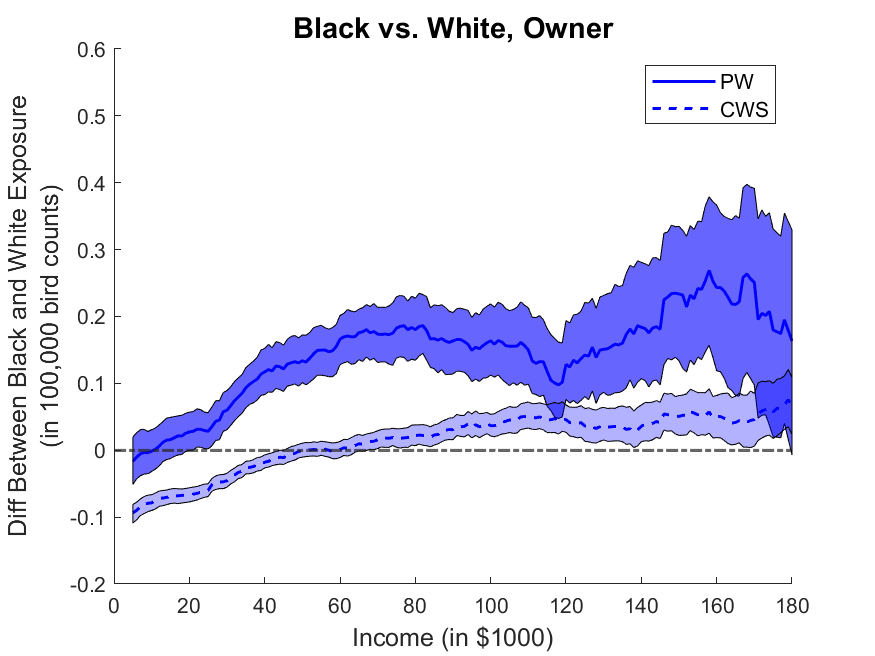


Figure (23a)


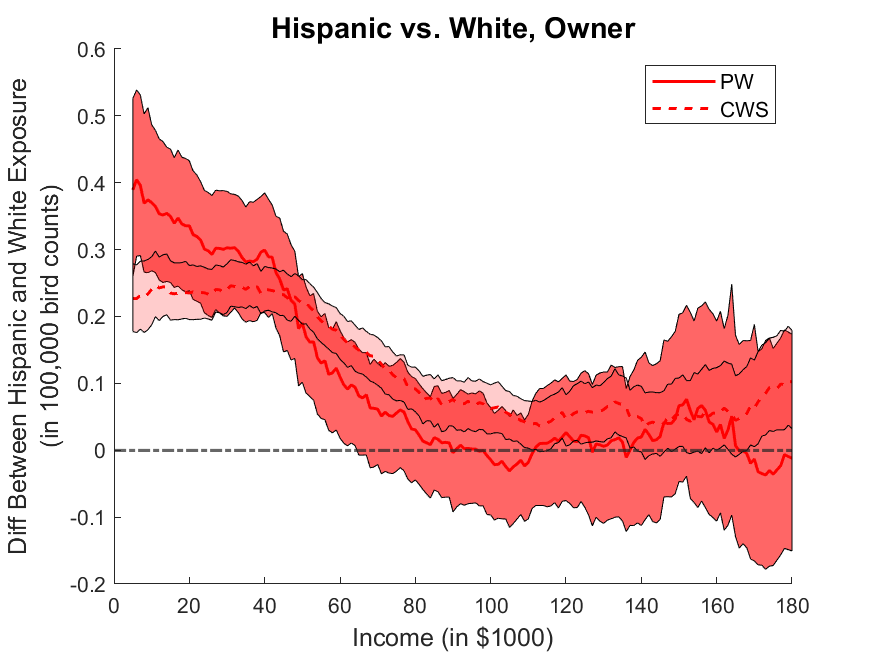


Figure (23b)


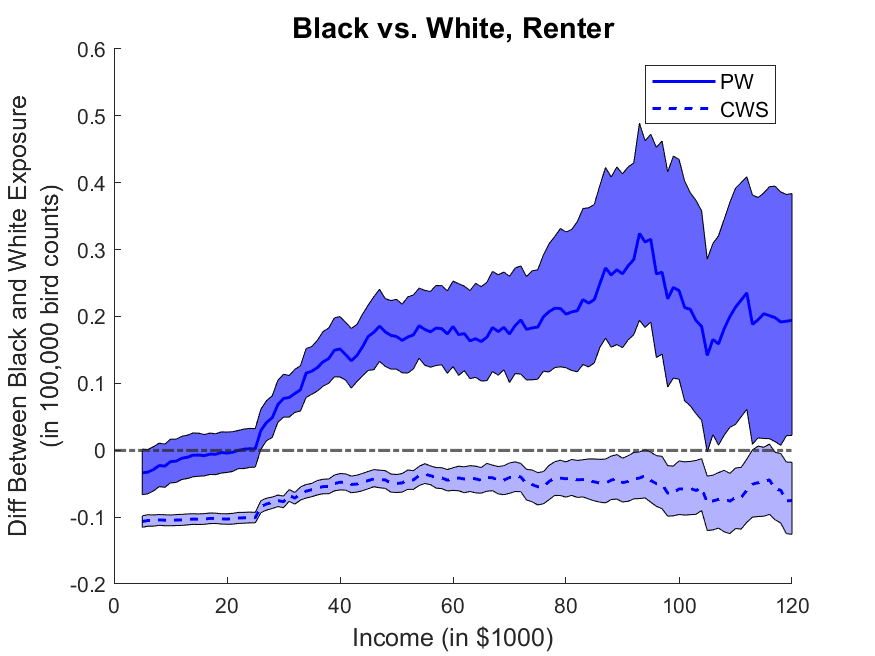


Figure (23c)


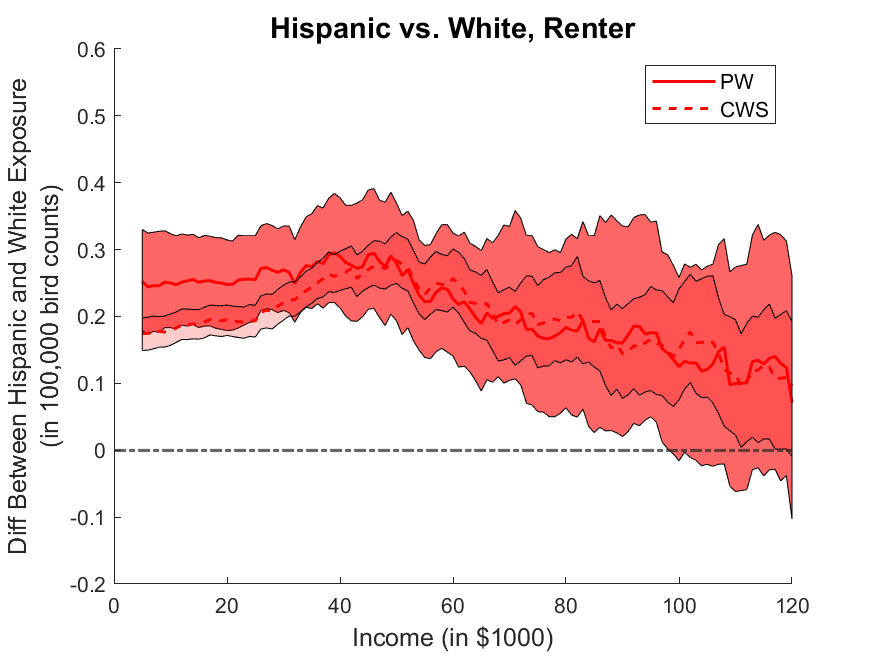


Figure (23d)

**S23 Fig. 4km Poultry Exposure Race Difference by Owner/Renter, Private Well vs Community Water System.**

We use poultry farm data (provided by Environmental Working Group) and household demographic data (InfoUSA) to calculate the poultry exposure (within 4km) difference (in 100,000 bird counts) between minority group and white for private well and community water system dependent owners and renters. The lines show the exposure differences between Black/Hispanic and white residents, where the solid and dashed lines show the exposure differences for private well and community water system dependent households. The shaded area shows the corresponding 95% confidence interval.


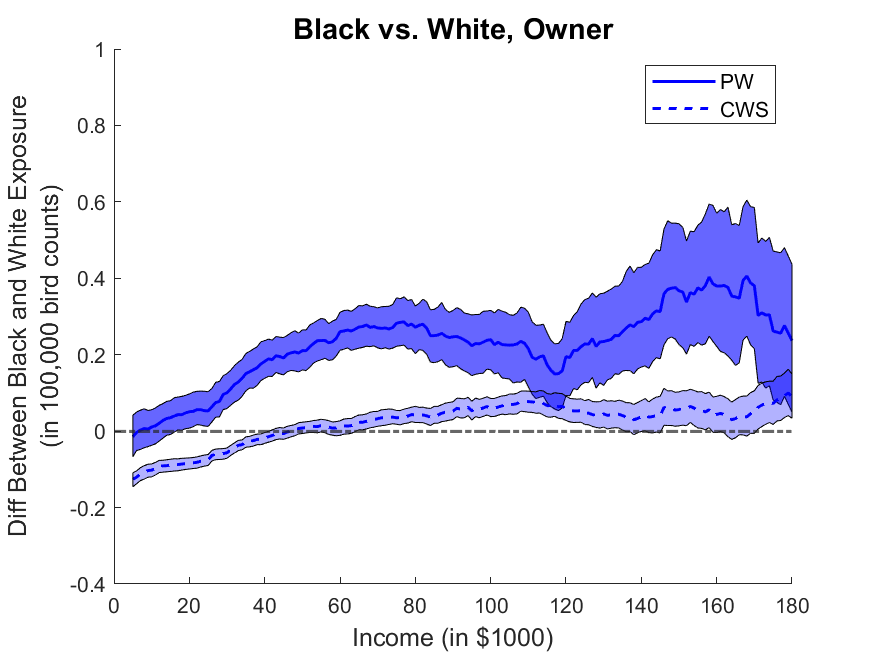


Figure (24a)


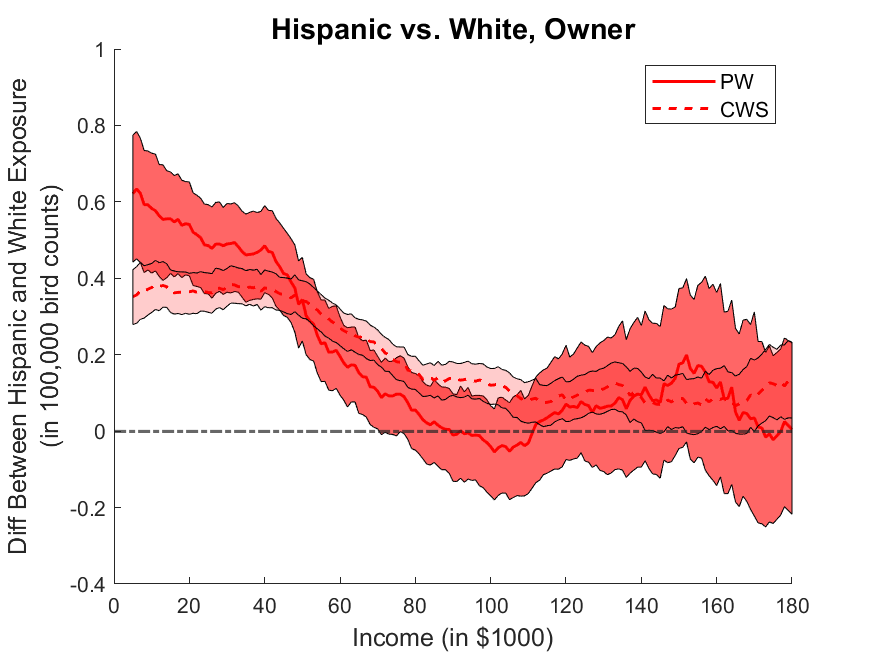


Figure (24b)


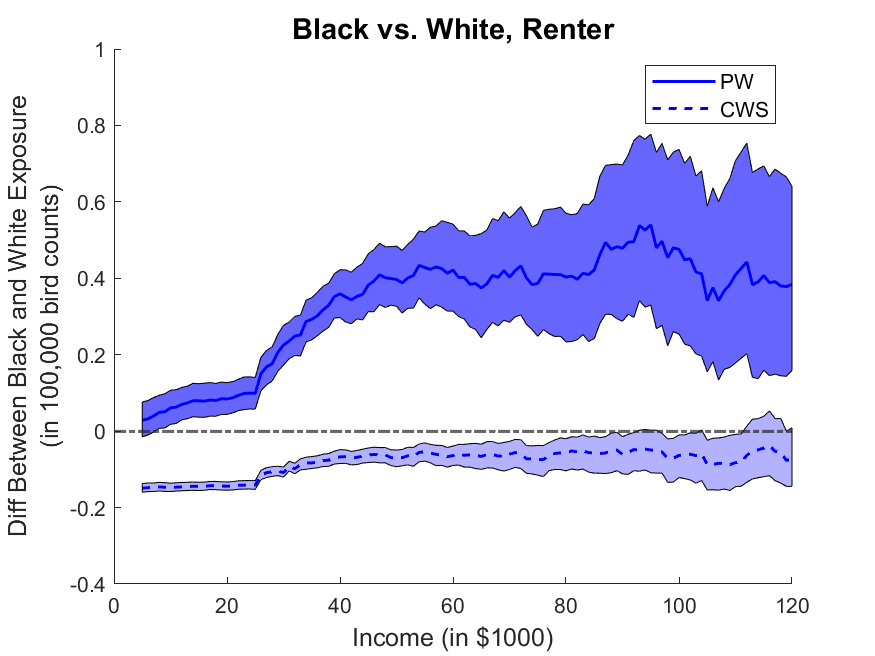


Figure (24c)


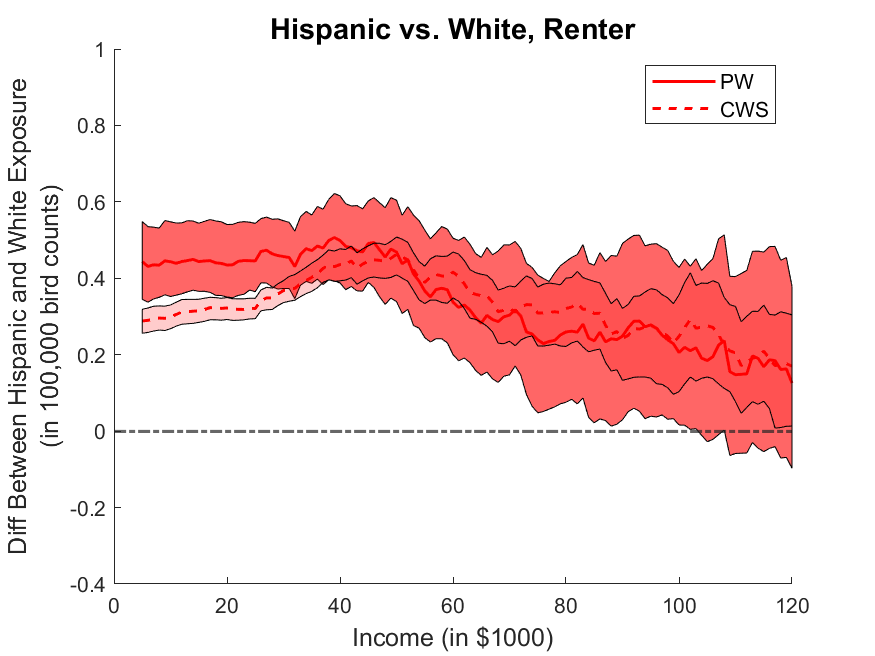


Figure (24d)

**S24 Fig. 5km Poultry Exposure Race Difference by Owner/Renter, Private Well vs Community Water System.**

We use poultry farm data (provided by Environmental Working Group) and household demographic data (InfoUSA) to calculate the poultry exposure (within 5km) difference (in 100,000 bird counts) between minority group and white for private well and community water system dependent owners and renters. The lines show the exposure differences between Black/Hispanic and white residents, where the solid and dashed lines show the exposure differences for private well and community water system dependent households. The shaded area shows the corresponding 95% confidence interval.

## **Exposure in major agricultural-producing counties.**

We run additional analyses by focusing on major agricultural-producing counties in eastern NC. In this section, we plot exposure for the top agricultural-producing counties in livestock farm cash receipts [28] and compare the exposure with other counties. The top ten counties are Duplin, Sampson, Bladen, Union, Robeson, Wilkes, Wayne, Anson, Randolph, and Bertie. The analyses focus on exposure within 5km buffer and owners (see Figs S25-S27).


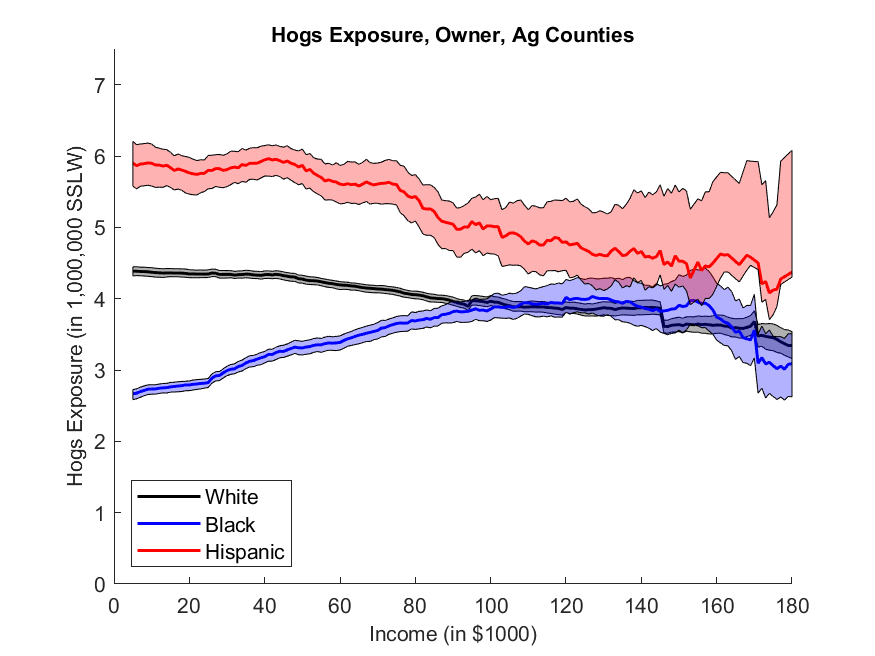


Figure (25a)


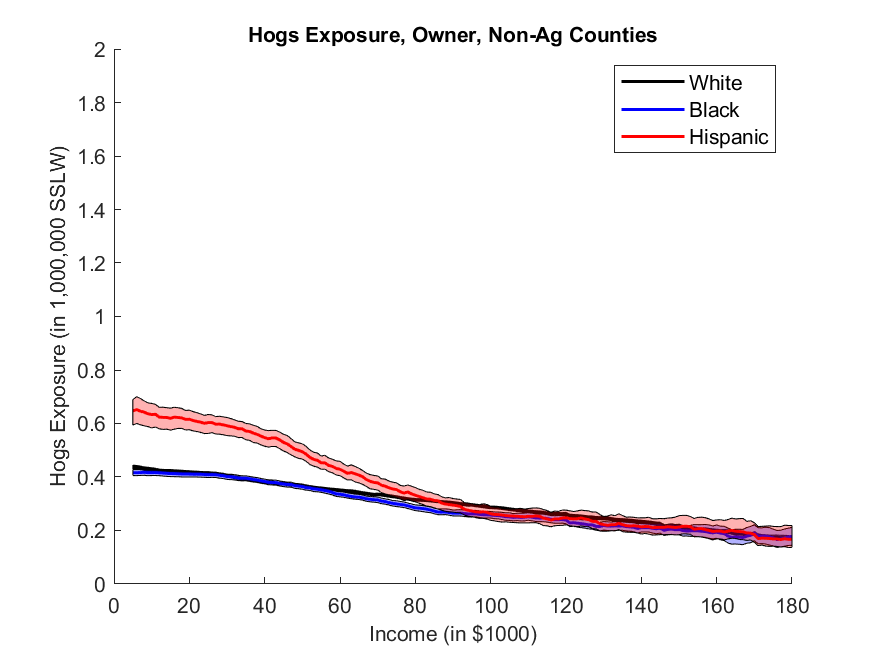


Figure (25b)


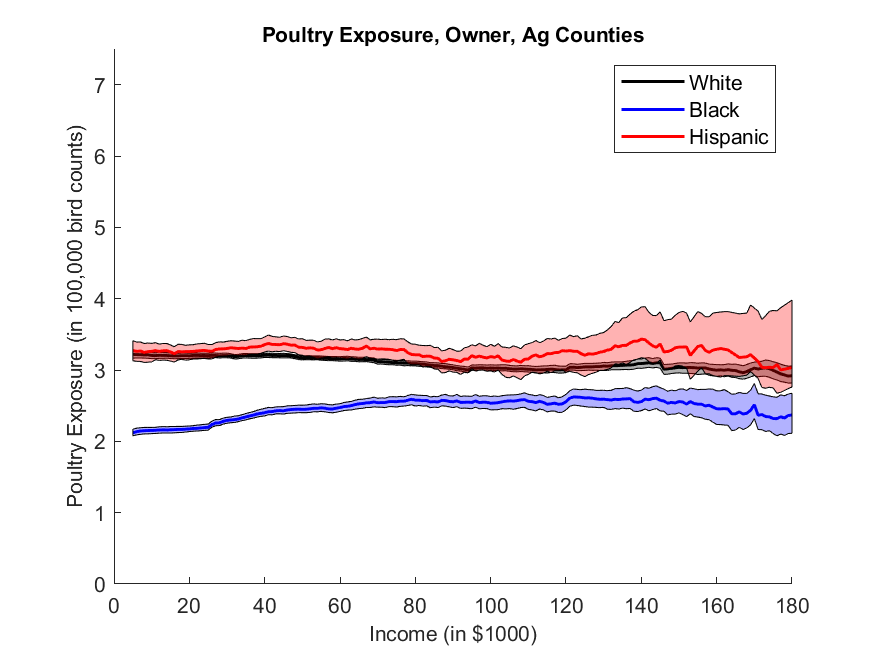


Figure (25c)


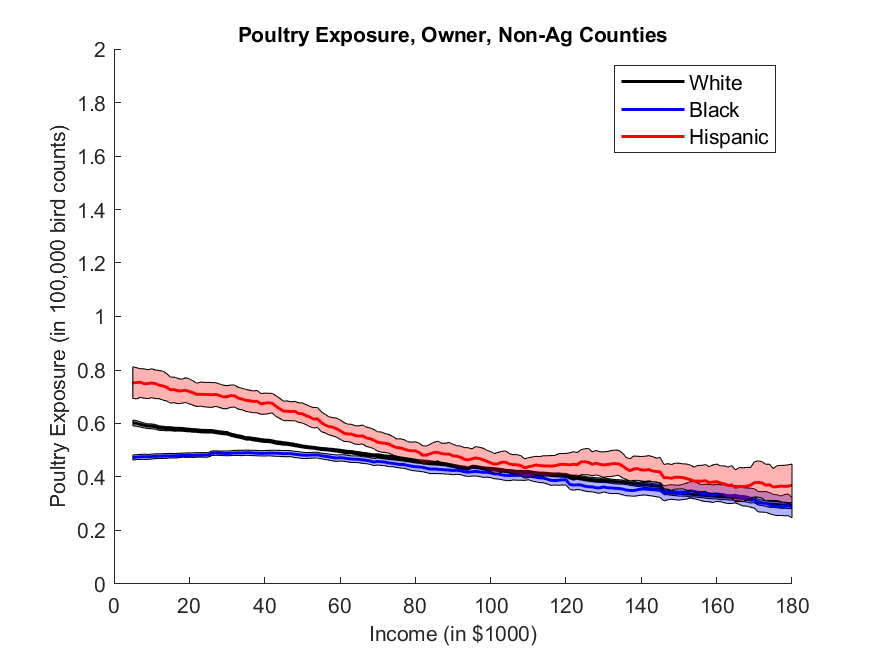


Figure (25d)

**S25 Fig. 5km Hogs and Poultry Exposure in Major Agricultural-Producing and Non-Agricultural-Producing Counties, by Race and Income.**

We use animal farm data (provided by NC Department of Environmental Quality and Environmental Working Group) and household demographic data (InfoUSA) to calculate the aggregated SSLW (in 1,000,000) and bird counts (in 100,000) within household's 5km buffer and corresponding 95% confidence interval at each income level for each race (white, Black, and Hispanic). The four panels show the hog/poultry exposure distribution for owners. The solid line shows the exposure, and shaded area shows the corresponding 95% confidence interval.


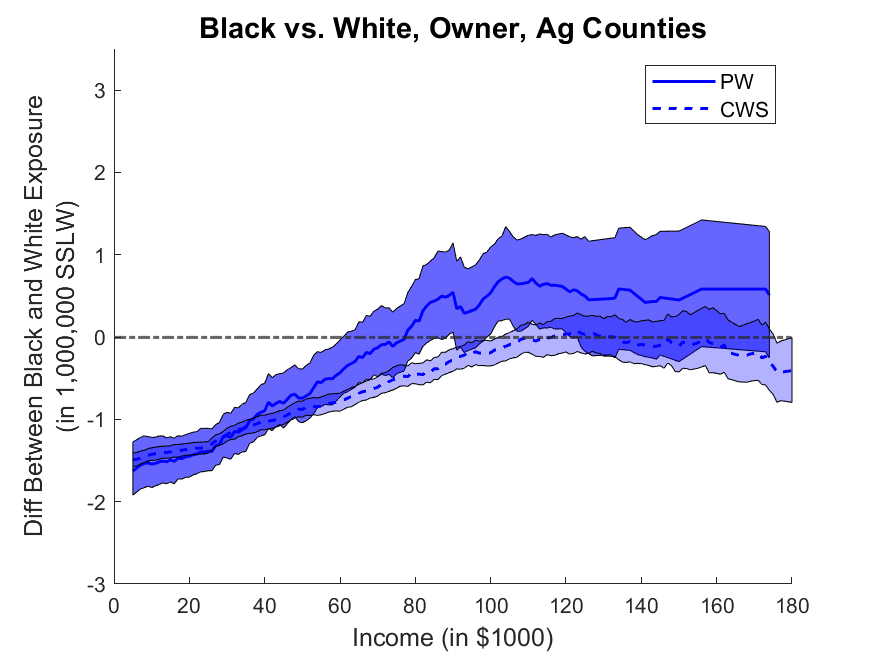


Figure (26a)


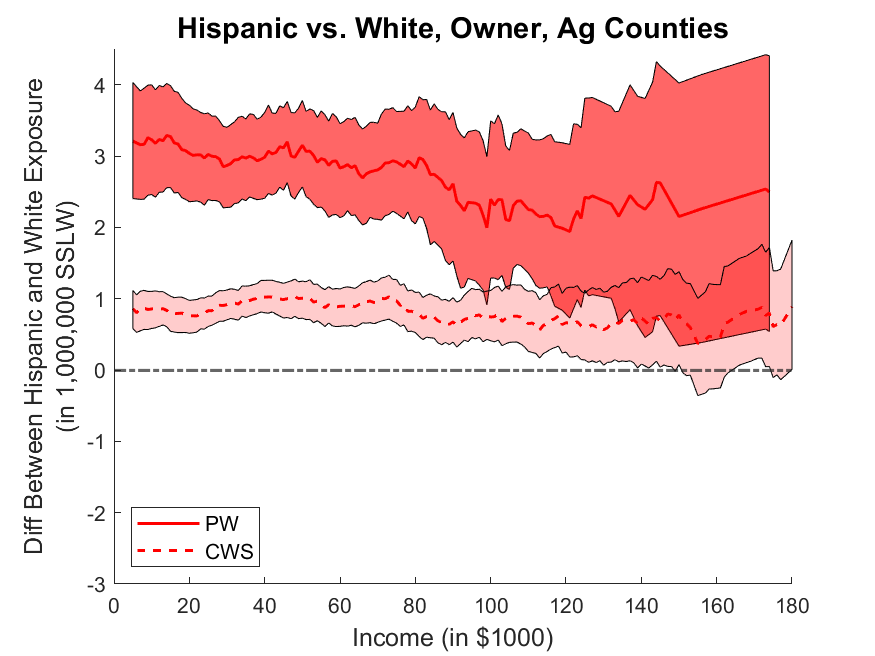


Figure (26b)


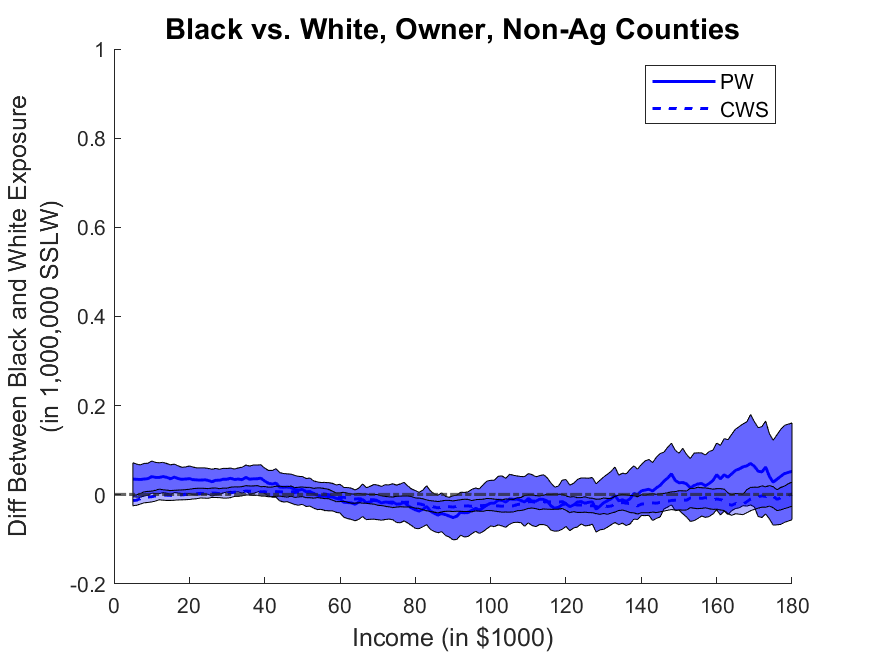


Figure (26c)


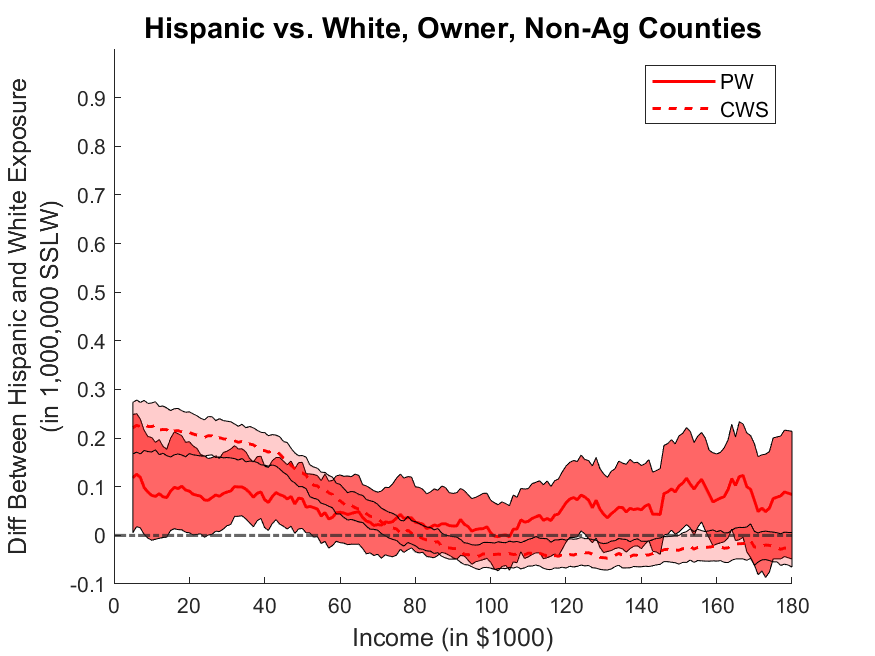


Figure (26d)

**S26 Fig. 5km Hogs Exposure Race Difference in Major Agricultural-Producing and Non-Agricultural-Producing Counties, Private Well vs Community Water System.**

We use hog farm data (provided by NC Department of Environmental Quality) and household demographic data (InfoUSA) to calculate the hog exposure (within 5km) difference (in 1,000,000 SSLW) between minority group and white for private well and community water system dependent owners. The lines show the exposure differences between Black/Hispanic and white residents, where the solid and dashed lines show the exposure differences for private well and community water system dependent households. The shaded area shows the corresponding 95% confidence interval.


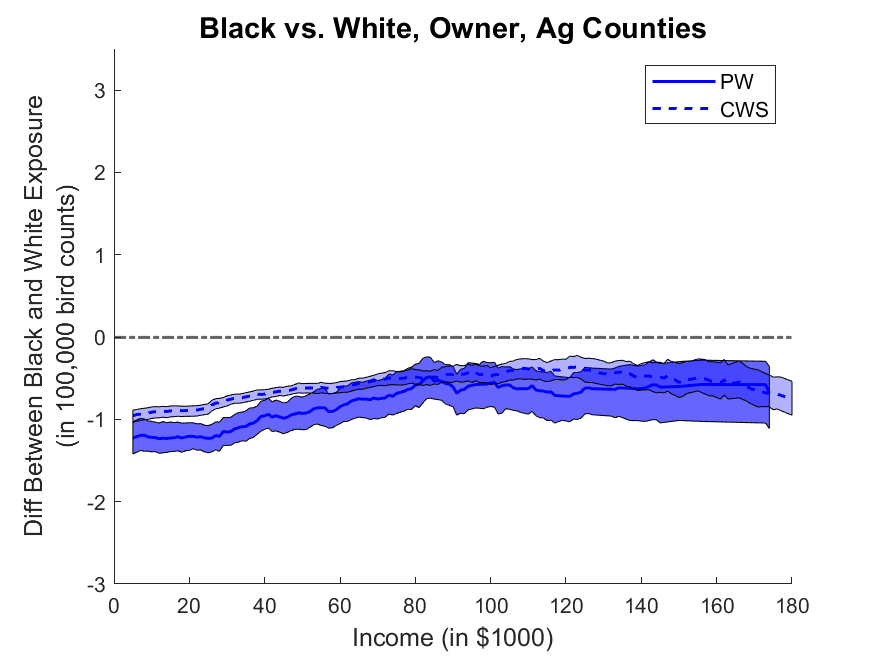


Figure (27a)


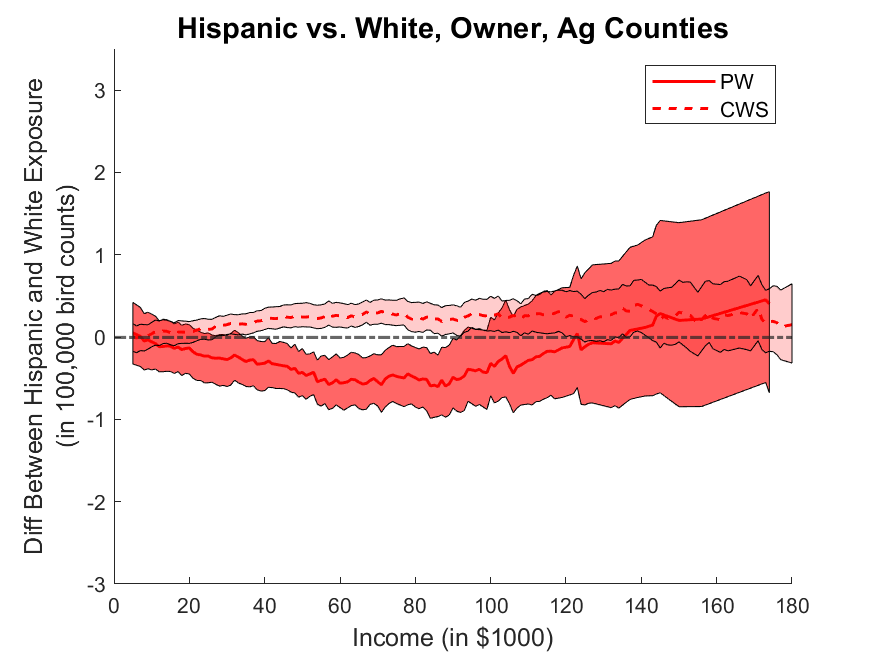


Figure (27b)


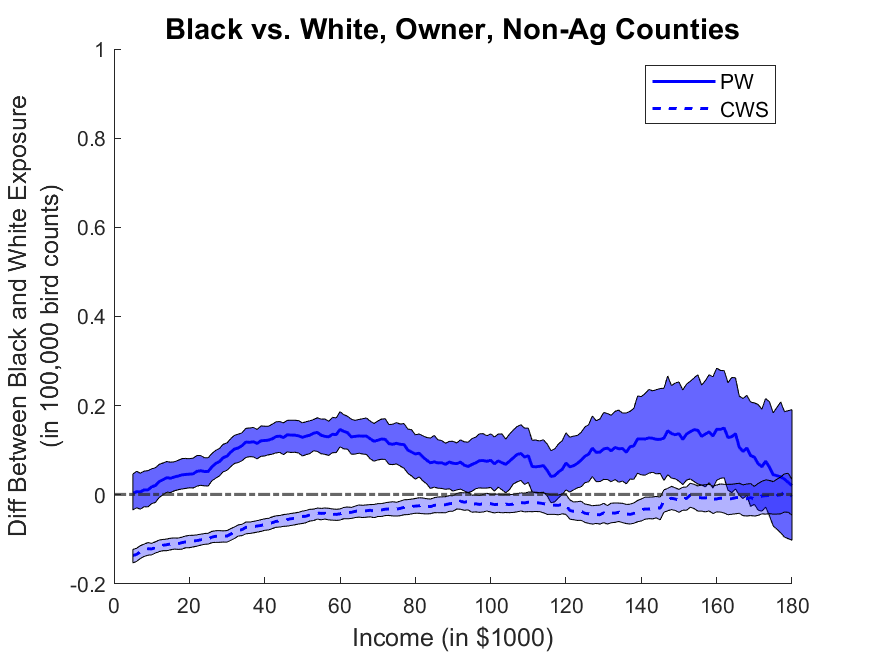


Figure (27c)


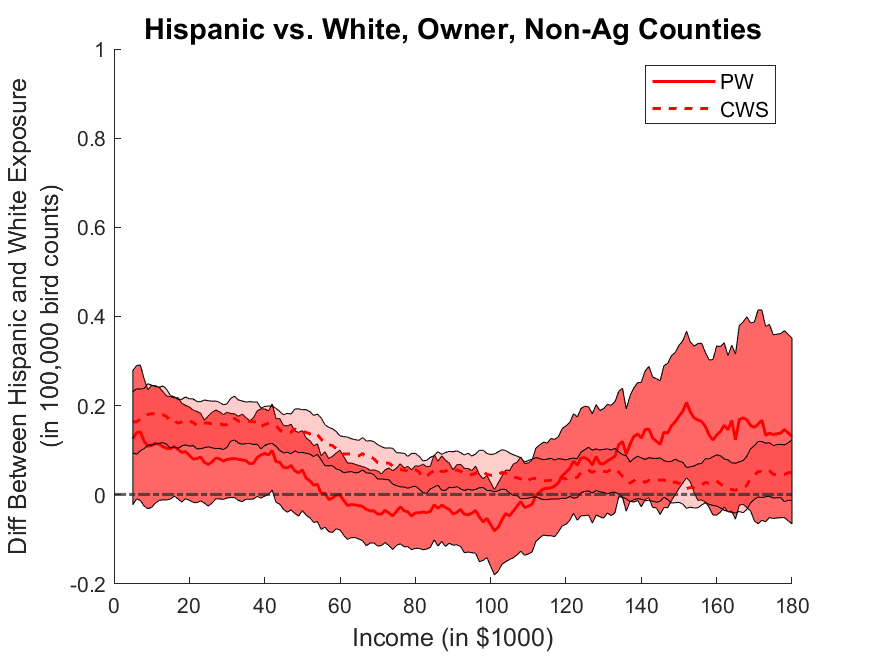


Figure (27d)

**S27 Fig. 5km Poultry Exposure Race Difference in Major Agricultural-Producing and Non-Agricultural-Producing Counties, Private Well vs Community Water System.**

We use poultry farm data (provided by Environmental Working Group) and household demographic data (InfoUSA) to calculate the poultry exposure (within 5km) difference (in 100,000 bird counts) between minority group and white for private well and community water system dependent owners. The lines show the exposure differences between Black/Hispanic and white residents, where the solid and dashed lines show the exposure differences for private well and community water system dependent households. The shaded area shows the corresponding 95% confidence interval.

## **Exposure patterns when considering Native Americans.**

Because U.S. census show some areas in NC contain high percentage of Native American, such as 41% in Robeson County. Including those counties in our analyses could bias our results because those Native Americans may be identified in a wrong racial category. We run additional analyses by dropping those counties (Robeson and Scotland counties). Results (see Figs S28-S30) are similar compared to the main specifications.


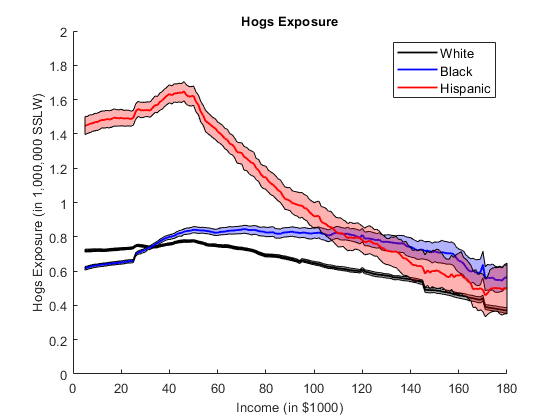


Figure (28a)


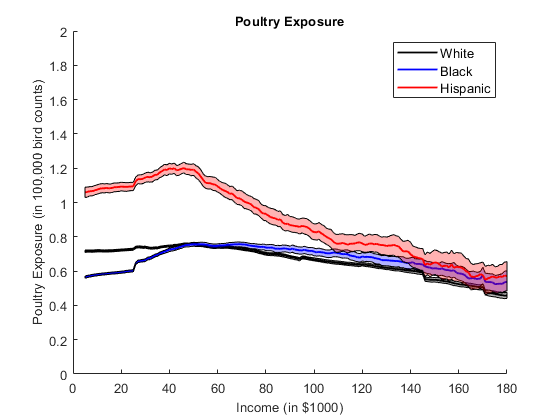


Figure (28b)

**S28 Fig. 5km CAFO Exposure (dropping Robeson and Scotland Counties).**

We use animal farm data (provided by NC Department of Environmental Quality and Environmental Working Group) and household demographic data (InfoUSA) to calculate the aggregated SSLW (in 1,000,000) and bird counts (in 100,000) within household's 5km buffer and corresponding 95% confidence interval at each income level for each race (white, Black, and Hispanic). The four panels show the hog/poultry exposure distribution for owners. The solid line shows the exposure, and shaded area shows the corresponding 95% confidence interval.


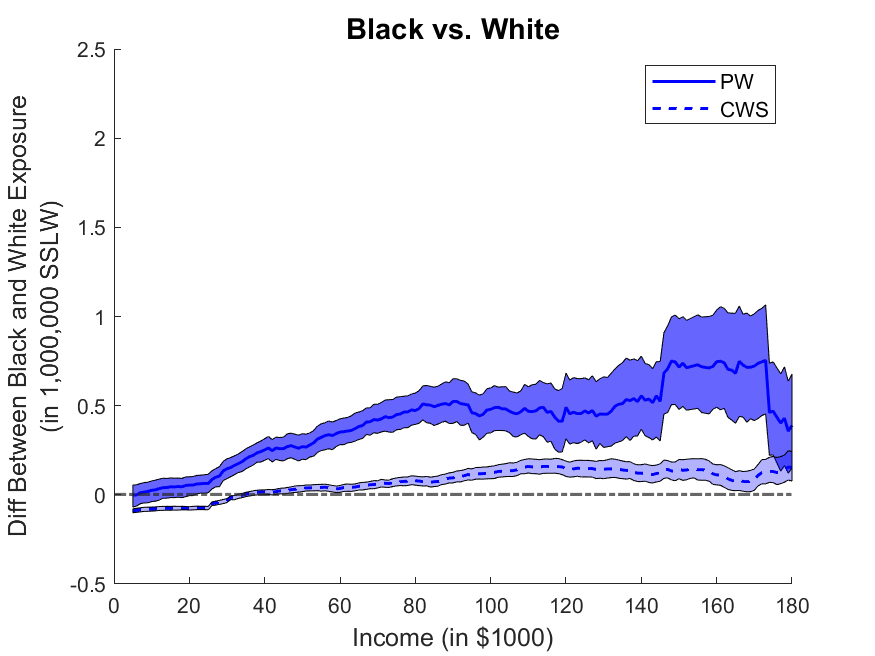


Figure (29a)


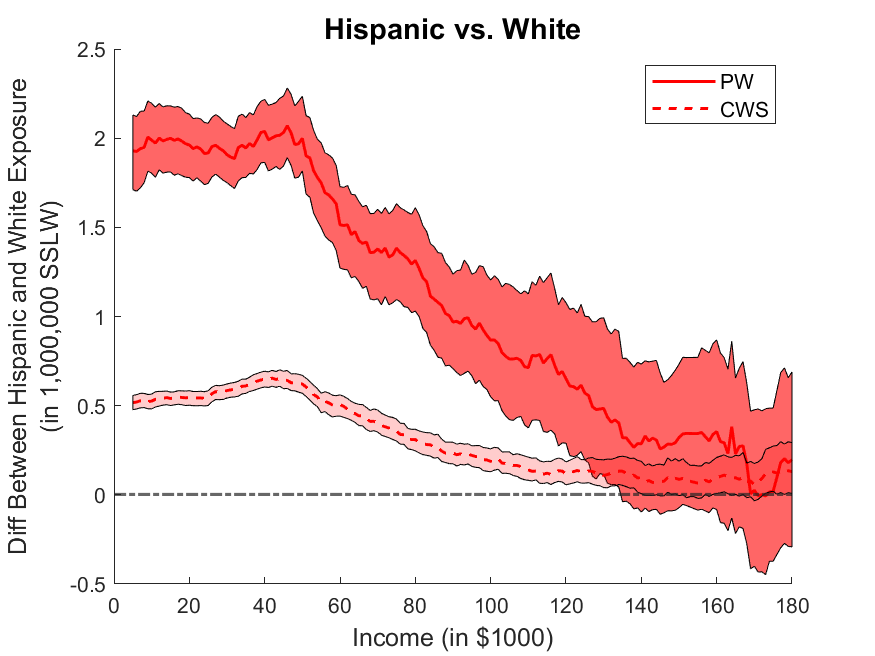


Figure (29b)

**S29 Fig. 5km Hogs Exposure Race Difference (dropping Robeson and Scotland Counties).**

We use hog farm data (provided by NC Department of Environmental Quality) and household demographic data (InfoUSA) to calculate the hog exposure (within 5km) difference (in 1,000,000 SSLW) between minority group and white for private well and community water system dependent owners. The lines show the exposure differences between Black/Hispanic and white residents, where the solid and dashed lines show the exposure differences for private well and community water system dependent households. The shaded area shows the corresponding 95% confidence interval.


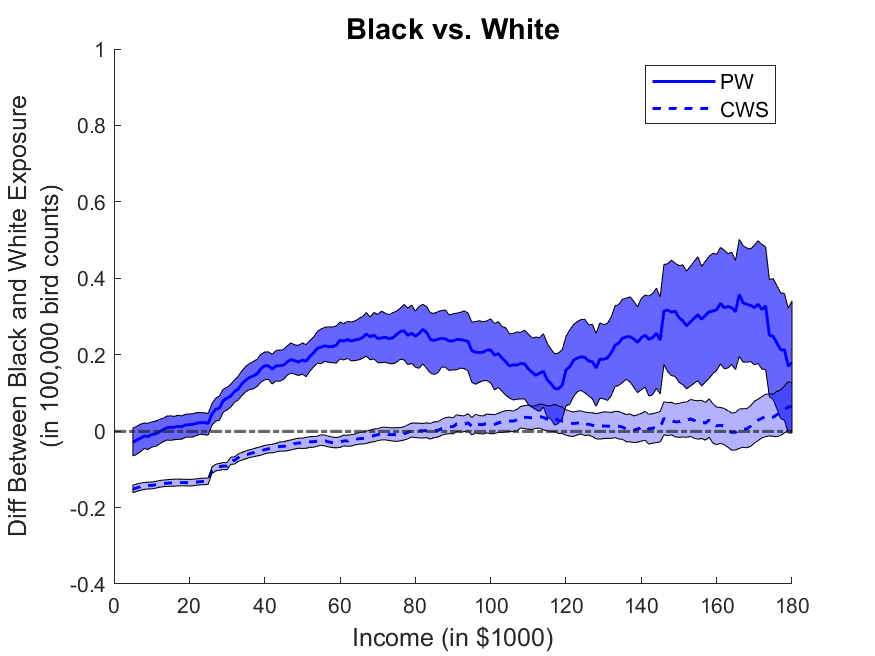


Figure (30a)


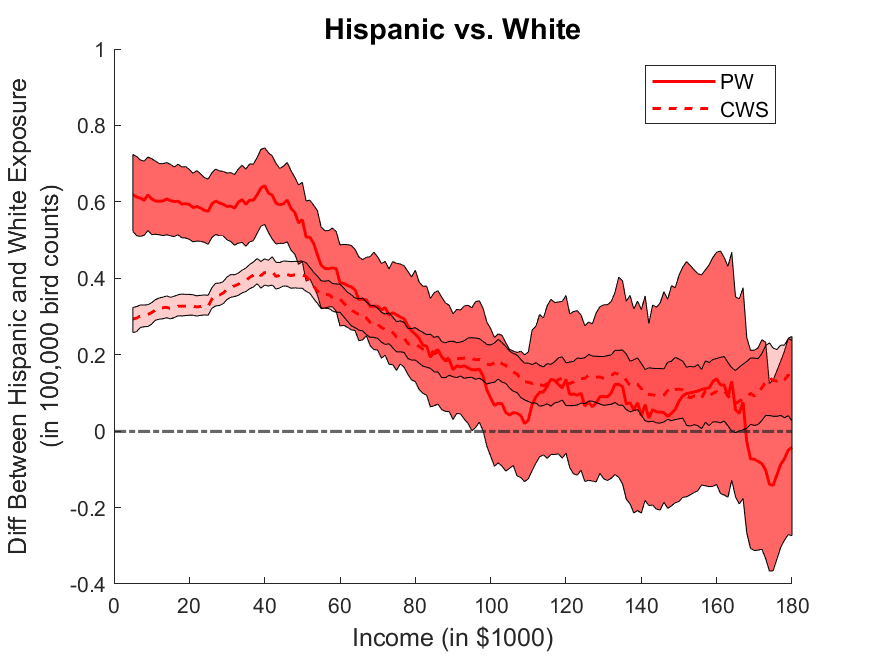


Figure (30b)

**S30 Fig. 5km Poultry Exposure Race Difference (dropping Robeson and Scotland Counties).**

We use poultry farm data (provided by Environmental Working Group) and household demographic data (InfoUSA) to calculate the poultry exposure (within 5km) difference (in 100,000 bird counts) between minority group and white for private well and community water system dependent owners. The lines show the exposure differences between Black/Hispanic and white residents, where the solid and dashed lines show the exposure differences for private well and community water system dependent households. The shaded area shows the corresponding 95% confidence interval.

## **Bivariate probit analysis.**

This section shows the four predicted join probabilities from bivariate probit models: $P(Hogs=0,Poultry=0)$, $P(Hogs=0,Poultry=1)$, $P(Hogs=1,Poultry=0)$, and $P(Hogs=1,Poultry=1)$ for 3km, 4km, and 5km exposure. In the bivariate probit model, we define outcome as 1 if the CAFO exposure is positive and 0 otherwise. The independent variables are race by income dummies (see Tables S5-S7).

**S5 Table. CAFO Exposure - Predicted Probability (Hogs, Poultry), 3km**

|  | $P(Hogs=0,$  $Poultry=0)$ | $P(Hogs=0,$  $Poultry=1)$ | $P(Hogs=1,$  $Poultry=0)$ | $P(Hogs=1,$  $Poultry=1)$ |
| --- | --- | --- | --- | --- |
| White Low Income | 0.754*** (0.000735) | 0.0856***  (0.000466) | 0.0711***  (0.000422) | 0.0894***  (0.000429) |
| Black Low Income | 0.757***  (0.00105) | 0.104***  (0.000730) | 0.0544***  (0.000507) | 0.0846***  (0.000581) |
| Hispanic Low Income | 0.661***  (0.00303) | 0.0905***  (0.00171) | 0.104***  (0.00186) | 0.145***  (0.00196) |
| White Medium Income | 0.742***  (0.000657) | 0.0876***  (0.000421) | 0.0744***  (0.000387) | 0.0958***  (0.000401) |
| Black Medium Income | 0.714***  (0.00146) | 0.108***  (0.000970) | 0.0680***  (0.000741) | 0.110***  (0.000870) |
| Hispanic Medium Income | 0.652***  (0.00323) | 0.0979***  (0.00190) | 0.0997***  (0.00192) | 0.150***  (0.00212) |
| White High Income | 0.771***  (0.000951) | 0.0875***  (0.000620) | 0.0619***  (0.000512) | 0.0800***  (0.000519) |
| Black High Income | 0.736***  (0.00309) | 0.100***  (0.00202) | 0.0660***  (0.00159) | 0.0982***  (0.00173) |
| Hispanic High Income | 0.721***  (0.00572) | 0.0887***  (0.00344) | 0.0822***  (0.00330) | 0.108***  (0.00329) |
| N | 1010618 | 1010618 | 1010618 | 1010618 |

Standard errors in parentheses, ***p<0.01, **p<0.05, *p<0.1

Four predicted join probabilities from bivariate probit models: $P(Hogs=0,Poultry=0)$, $P(Hogs=0,Poultry=1)$, $P(Hogs=1,Poultry=0)$), and $P(Hogs=1,Poultry=1)$. In the bivariate probit model, we define outcome as 1 if the CAFO exposure is positive and 0 otherwise. The independent variables are race by income dummies. The low-income group includes households with income below $35,000, median income group includes household with income between $35,000 and $100,00, and high-income group includes households with income above $100,000.

**S6 Table. CAFO Exposure - Predicted Probability (Hogs, Poultry), 4km**

|  | $P(Hogs=0,$  $Poultry=0)$ | $P(Hogs=0,$  $Poultry=1)$ | $P(Hogs=1,$  $Poultry=0)$ | $P(Hogs=1,$  $Poultry=1)$ |
| --- | --- | --- | --- | --- |
| White Low Income | 0.675***  (0.000781) | 0.0969***  (0.000481) | 0.0883***  (0.000456) | 0.140***  (0.000526) |
| Black Low Income | 0.659***  (0.00110) | 0.116***  (0.000722) | 0.0763***  (0.000567) | 0.148***  (0.000742) |
| Hispanic Low Income | 0.577***  (0.00309) | 0.105***  (0.00179) | 0.110***  (0.00183) | 0.207***  (0.00230) |
| White Medium Income | 0.667***  (0.000698) | 0.103***  (0.000446) | 0.0854***  (0.000403) | 0.145***  (0.000481) |
| Black Medium Income | 0.621***  (0.00152) | 0.119***  (0.000970) | 0.0855***  (0.000796) | 0.175***  (0.00107) |
| Hispanic Medium Income | 0.567***  (0.00330) | 0.116***  (0.00201) | 0.102***  (0.00185) | 0.214***  (0.00249) |
| White High Income | 0.696***  (0.00100) | 0.110***  (0.000667) | 0.0689***  (0.000511) | 0.124***  (0.000638) |
| Black High Income | 0.657***  (0.00320) | 0.113***  (0.00203) | 0.0795***  (0.00165) | 0.150***  (0.00210) |
| Hispanic High Income | 0.629***  (0.00586) | 0.118***  (0.00375) | 0.0836***  (0.00305) | 0.169***  (0.00401) |
| N | 1038852 | 1038852 | 1038852 | 1038852 |

Standard errors in parentheses, ***p<0.01, **p<0.05, *p<0.1

Four predicted join probabilities from bivariate probit models: $P(Hogs=0,Poultry=0)$, $P(Hogs=0,Poultry=1)$, $P(Hogs=1,Poultry=0)$), and $P(Hogs=1,Poultry=1)$. In the bivariate probit model, we define outcome as 1 if the CAFO exposure is positive and 0 otherwise. The independent variables are race by income dummies. The low-income group includes households with income below $35,000, median income group includes household with income between $35,000 and $100,00, and high-income group includes households with income above $100,000.

**S7 Table. CAFO Exposure - Predicted Probability (Hogs, Poultry), 5km**

|  | $P(Hogs=0,$  $Poultry=0)$ | $P(Hogs=0,$  $Poultry=1)$ | $P(Hogs=1,$  $Poultry=0)$ | $P(Hogs=1,$  $Poultry=1)$ |
| --- | --- | --- | --- | --- |
| White Low Income | 0.606***  (0.000808) | 0.107***  (0.000494) | 0.0944***  (0.000461) | 0.193***  (0.000605) |
| Black Low Income | 0.566***  (0.00112) | 0.130***  (0.000728) | 0.0844***  (0.000568) | 0.220***  (0.000875) |
| Hispanic Low Income | 0.509***  (0.00310) | 0.116***  (0.00184) | 0.108***  (0.00176) | 0.268***  (0.00257) |
| White Medium Income | 0.605***  (0.000724) | 0.110***  (0.000454) | 0.0924***  (0.000414) | 0.193***  (0.000546) |
| Black Medium Income | 0.539***  (0.00155) | 0.132***  (0.000995) | 0.0883***  (0.000786) | 0.241***  (0.00124) |
| Hispanic Medium Income | 0.501***  (0.00331) | 0.126***  (0.00206) | 0.0995***  (0.00178) | 0.273***  (0.00278) |
| White High Income | 0.634  (0.00104) | 0.122***  (0.000681) | 0.0739***  (0.000512) | 0.170***  (0.000736) |
| Black High Income | 0.583***  (0.00327) | 0.120*** (0.00201) | 0.0887***  (0.00168) | 0.208***  (0.00244) |
| Hispanic High Income | 0.560***  (0.00589) | 0.135***  (0.00385) | 0.0816***  (0.00284) | 0.223***  (0.00453) |
| N | 1057216 | 1057216 | 1057216 | 1057216 |

Standard errors in parentheses, ***p<0.01, **p<0.05, *p<0.1

Four predicted join probabilities from bivariate probit models: $P(Hogs=0,Poultry=0)$, $P(Hogs=0,Poultry=1)$, $P(Hogs=1,Poultry=0)$), and $P(Hogs=1,Poultry=1)$. In the bivariate probit model, we define outcome as 1 if the CAFO exposure is positive and 0 otherwise. The independent variables are race by income dummies. The low-income group includes households with income below $35,000, median income group includes household with income between $35,000 and $100,00, and high-income group includes households with income above $100,000.

## **Bivariate probit equality test.**

This section tests equality of predicted probabilities in the supporting information. Those tables (3/4/5km buffers, see Tables S8-S10) compare whether coefficients of minorities are larger than white and compare within income groups. Results show minorities are more statistically more likely to be exposed to both hogs and poultry. Such probabilities are larger for Hispanic: the coefficients are larger for Hispanic, compared to Black residents.

**S8 Table. CAFO Exposure – Probability Equality Test (Hogs, Poultry), 3km**

|  | $P(Hogs=0,$  $Poultry=0)$ | $P(Hogs=0,$  $Poultry=1)$ | $P(Hogs=1,$  $Poultry=0)$ | $P(Hogs=1,$  $Poultry=1)$ |
| --- | --- | --- | --- | --- |
| $\beta_{B^{low}}-\beta_{W^{low}}$ | .0035*** | .018*** | -.0167*** | -.00487*** |
| $\beta_{H^{low}}-\beta_{W^{low}}$ | -.093*** | .0049*** | .0331*** | .0551*** |
| $\beta_{B^{med}}-\beta_{W^{med}}$ | -.028*** | .0103*** | -.0064*** | .01409*** |
| $\beta_{H^{med}}-\beta_{W^{med}}$ | -.09*** | .0203*** | .0253*** | .0545*** |
| $\beta_{B^{high}}-\beta_{W^{high}}$ | -.035*** | .0127*** | .00406*** | .01815*** |
| $\beta_{H^{high}}-\beta_{W^{high}}$ | -.049*** | .0011*** | .02032*** | .02777*** |

*** p<0.01, ** p<0.05, * p<0.1

Four predicted join probabilities from bivariate probit models: $P(Hogs=0,Poultry=0)$, $P(Hogs=0,Poultry=1)$, $P(Hogs=1,Poultry=0)$), and $P(Hogs=1,Poultry=1)$. In the bivariate probit model, we define outcome as 1 if the CAFO exposure is positive and 0 otherwise. The independent variables are race by income dummies. This table examines whether the coefficients of the minorities are larger than white.

**S9 Table. CAFO Exposure - Probability Equality Test (Hogs, Poultry), 4km**

|  | $P(Hogs=0,$  $Poultry=0)$ | $P(Hogs=0,$  $Poultry=1)$ | $P(Hogs=1,$  $Poultry=0)$ | $P(Hogs=1,$  $Poultry=1)$ |
| --- | --- | --- | --- | --- |
| $\beta_{B^{low}}-\beta_{W^{low}}$ | -.0156*** | .01947*** | -.01197*** | .00815*** |
| $\beta_{H^{low}}-\beta_{W^{low}}$ | -.0972*** | .00843*** | .02178*** | .06701*** |
| $\beta_{B^{med}}-\beta_{W^{med}}$ | -.0464*** | .01619*** | .00017 | .03009*** |
| $\beta_{H^{med}}-\beta_{W^{med}}$ | -.0996*** | .0133*** | .01659*** | .06969*** |
| $\beta_{B^{high}}-\beta_{W^{high}}$ | -.03916*** | .00293*** | .01064*** | .025584*** |
| $\beta_{H^{high}}-\beta_{W^{high}}$ | -.0672*** | .00818*** | .01475*** | .04432*** |

*** p<0.01, ** p<0.05, * p<0.1

Four predicted join probabilities from bivariate probit models: $P(Hogs=0,Poultry=0)$, $P(Hogs=0,Poultry=1)$, $P(Hogs=1,Poultry=0)$), and $P(Hogs=1,Poultry=1)$. In the bivariate probit model, we define outcome as 1 if the CAFO exposure is positive and 0 otherwise. The independent variables are race by income dummies. This table examines whether the coefficients of the minorities are larger than white.

**S10 Table. CAFO Exposure - Probability Equality Test (Hogs, Poultry), 5km**

|  | $P(Hogs=0,$  $Poultry=0)$ | $P(Hogs=0,$  $Poultry=1)$ | $P(Hogs=1,$  $Poultry=0)$ | $P(Hogs=1,$  $Poultry=1)$ |
| --- | --- | --- | --- | --- |
| $\beta_{B^{low}}-\beta_{W^{low}}$ | -.04007*** | .02273*** | -.01003*** | .027376*** |
| $\beta_{H^{low}}-\beta_{W^{low}}$ | -.09675*** | .00841*** | .013304*** | .07503*** |
| $\beta_{B^{med}}-\beta_{W^{med}}$ | -.0658*** | .02198*** | -.00408*** | .04793*** |
| $\beta_{H^{med}}-\beta_{W^{med}}$ | -.10364*** | .01624*** | .007149*** | .08025*** |
| $\beta_{B^{high}}-\beta_{W^{high}}$ | -.05088*** | -.0021 | .014737*** | .03825*** |
| $\beta_{H^{high}}-\beta_{W^{high}}$ | -.07429*** | .012984*** | .00764*** | .05366*** |

*** p<0.01, ** p<0.05, * p<0.1

Four predicted join probabilities from bivariate probit models: $P(Hogs=0,Poultry=0)$, $P(Hogs=0,Poultry=1)$, $P(Hogs=1,Poultry=0)$), and $P(Hogs=1,Poultry=1)$. In the bivariate probit model, we define outcome as 1 if the CAFO exposure is positive and 0 otherwise. The independent variables are race by income dummies. This table examines whether the coefficients of the minorities are larger than white.

# **SI References**

1. Trusts, Pew Charitable, and Johns Hopkins. "Putting meat on the table: Industrial farm animal production in America." A Report of the Pew Commission on Industrial Farm Animal Production, A Project of The Pew Charitable Trusts and Johns Hopkins Bloomberg School of Public Health (2008).
2. Christenson, Elizabeth C., and Marc L. Serre. "Integrating remote sensing with nutrient management plans to calculate nitrogen parameters for swine CAFOs at the sprayfield and sub-watershed scales." Science of the Total Environment 580 (2017): 865-872.
3. Wing, Steve, Dana Cole, and Gary Grant. "Environmental injustice in North Carolina's hog industry." *Environmental health perspectives* 108.3 (2000): 225-231.
4. Cole, Dana, Lori Todd, and Steve Wing. "Concentrated swine feeding operations and public health: a review of occupational and community health effects." Environmental health perspectives 108.8 (2000): 685-699.
5. Hribar, Carrie. "Understanding concentrated animal feeding operations and their impact on communities." (2010).
6. Horton, Rachel Avery, et al. "Malodor as a trigger of stress and negative mood in neighbors of industrial hog operations." American journal of public health 99.S3 (2009): S610-S615.
7. Sherlock, Robert R., et al. "Ammonia, methane, and nitrous oxide emission from pig slurry applied to a pasture in New Zealand." Journal of Environmental Quality 31.5 (2002): 1491-1501.
8. Schinasi, Leah, et al. "Air pollution, lung function, and physical symptoms in communities near concentrated swine feeding operations." Epidemiology 22.2 (2011): 208-215.
9. Aneja, Viney P., William H. Schlesinger, and Jan Willem Erisman. "Effects of agriculture upon the air quality and climate: research, policy, and regulations." (2009): 4234-4240.
10. Wing, Steve, Rachel Avery Horton, and Kathryn M. Rose. "Air pollution from industrial swine operations and blood pressure of neighboring residents." *Environmental health perspectives* 121.1 (2013): 92-96.
11. Von Essen, Susanna G., and Brent W. Auvermann. "Health effects from breathing air near CAFOs for feeder cattle or hogs." Journal of agromedicine 10.4 (2005): 55-64.
12. Mallin, Michael A., et al. "Industrial swine and poultry production causes chronic nutrient and fecal microbial stream pollution." Water, Air, & Soil Pollution 226 (2015): 1-13.
13. Ritz, C. W., B. D. Fairchild, and M. P. Lacy. "Implications of ammonia production and emissions from commercial poultry facilities: A review." Journal of applied poultry research 13.4 (2004): 684-692.
14. Pan, Leilei, and Simon X. Yang. "A new intelligent electronic nose system for measuring and analysing livestock and poultry farm odours." Environmental monitoring and assessment 135.1 (2007): 399-408.
15. Aneja, Viney P., William H. Schlesinger, and Jan Willem Erisman. "Effects of agriculture upon the air quality and climate: research, policy, and regulations." (2009): 4234-4240.
16. Dunlop, Mark W., Patrick J. Blackall, and Richard M. Stuetz. "Odour emissions from poultry litter–A review litter properties, odour formation and odorant emissions from porous materials." *Journal of environmental management* 177 (2016): 306-319.
17. Sorg, Lisa. “Unregulated, enormous poultry farms and their millions of birds again lie in hurricane's path”. NC Policy Watch (2019).

URL: <http://pulse.ncpolicywatch.org/2019/09/04/unregulated-enormous-poultry-farms-and-their-millions-of-birds-again-lie-in-hurricanes-path/#sthash.qApJHWyb.dpbs>

1. Kravchenko, Julia, et al. "Mortality and Health Outcomes in North Carolina Communities Located in Close Proximity to Hog Concentrated Animal Feeding Operations." *North Carolina medical journal* 79.5 (2018): 278-288.
2. Wing, Steve, and Susanne Wolf. "Intensive livestock operations, health, and quality of life among eastern North Carolina residents." *Environmental health perspectives* 108.3 (2000): 233-238.
3. Wing, Steve, and Jill Johnston. "Industrial hog operations in north carolina disproportionately impact african-americans, hispanics and american indians." *The University of North Carolina at Chapel Hill* (2014).
4. Chakraborty, Jayajit, et al. "Social and spatial inequities in exposure to flood risk in Miami, Florida." Natural Hazards Review 15.3 (2014): 04014006.
5. Brown, Phil. "Race, class, and environmental health: a review and systematization of the literature." *Environmental Research* 69.1 (1995): 15-30.
6. Wing, Steve, et al. "Community based collaboration for environmental justice: south-east Halifax environmental reawakening." *Environment and Urbanization* 8.2 (1996): 129-140.
7. Edwards, Bob, and Anthony E. Ladd. "Environmental justice, swine production and farm loss in North Carolina." *Sociological Spectrum* 20.3 (2000): 263-290.
8. NC Attorney General, Smithfield agreement (2000) Retrieved from NCSU Smithfield Project.193

URL: <https://projects.ncsu.edu/cals/waste_mgt/smithfield_projects/agreement.pdf>

1. Gonsenhauser, Rachel, et al. "Digitizing a statewide map of community water system service areas." (2020): 56-61.
2. Imai, Kosuke, and Kabir Khanna. "Improving ecological inference by predicting individual ethnicity from voter registration records." *Political Analysis* 24.2 (2016): 263-272.
3. USDA/NASS, 2022 North Carolina Agricultural Statistics (2022).

URL: <https://www.nass.usda.gov/Statistics_by_State/North_Carolina/Publications/Annual_Statistical_Bulletin/AgStat/NCAgStatBook.pdf>

1. Raff, Zach, and Andrew Meyer. "CAFOs and surface water quality: evidence from Wisconsin." American Journal of Agricultural Economics 104.1 (2022): 161-189.

1. The 5km exposure figures are in the manuscript. [↑](#footnote-ref-1)
